# Supplementary material for: Phylogenetic relationships of Neogene hamsters (Mammalia, Rodentia, Cricetinae) revealed under Bayesian inference and maximum parsimony
Source: PeerJ. 2024 Nov 15;12:e18440. doi: 10.7717/peerj.18440 (PMC11572387; doi:10.7717/peerj.18440)
Supplement: Supplemental Information 1 [file peerj-12-18440-s001.docx]

**Supplemental file S1 for**

Phylogenetic relationships of Neogene hamsters (Mammalia, Rodentia, Cricetinae) revealed under Bayesian inference and maximum parsimony

Moritz Dirnberger^1^, Pablo Peláez-Campomanes^2^, Raquel López-Antoñanzas^1^

^1^ISEM, Univ Montpellier, CNRS, IRD, Montpellier, France

^2^Departamento de Paleobiología, Museo Nacional de Ciencias Naturales-CSIC, Madrid, Spain

**Additional information about studied taxa and material**

**Extinct taxa**

53 valid species, that are clearly associated to one of the seven genera included in this study, are found in the literature. Of these 53 species, 41 could be coded and included in the phylogenetic analysis.

***Apocricetus*** Freudenthal, Mein & Martín Suárez, 1998

All of the 5 total species are included in the analysis:

***Apocricetus alberti*** Freudenthal, Mein & Martín Suárez, 1998

Occurrences: 32 (in Spain)

Type locality: Crevillente 6, Spain

Holotype: m1, CR6 111, Department Aardwetenschappen, University Utrecht (figured in de Bruijn et al. (1975), pl. 3, fig. 4, as *Cricetus* cf. *kormosi*)

Age range [Max–Min (Mean)]: 6.56–4.2 Ma (5.38 Ma)

Oldest occurrences: Arquillo 1, La Gloria 5, Las Casiones , Las Casiones superior, Masada del Valle 7, Purcal 23, Purcal 24A, Purcal 25, Purcal 25A, Valdecebro 3, Valdecebro 6, Villastar (van Dam et al., 2023)

Youngest occurrence: Alcoy-N (van Dam et al., 2023)

Anatomical references: (de Bruijn et al., 1975; Freudenthal, Lacomba & Martín Suárez, 1991; Freudenthal, Mein & Martín Suárez, 1998; García-Alix et al., 2008; Minwer-Barakat et al., 2009; Mansino et al., 2014; Piñero & Agustí, 2019)

***Apocricetus angustidens*** (Depéret, 1890)

Occurrences: 14 (in France [10] and Spain [4])

Type locality: Serrat d'en Vacquer, France

Holotype: mandible, Pp 75, Musée Guimet d'Histoire Naturelle, Lyon (Donnezan coll.) (figured in Depéret (1890-1895), pl. 4, figs. 26, 26a)

Age range: 4.27–3.03 Ma (3.65 Ma)

Oldest occurrences: Le Soler, Mont-Hélène, Nîmes, Sète, Serrat d'en Vacquer, Villeneuve de la Raho: MN 15 (Fejfar, 1970; Freudenthal, Mein & Martín Suárez, 1998)

Youngest occurrences: Puimoisson II, Lo Fournas 13 (Guerin, Mein & Truc, 1970; Biquand, Dubar & Sémah, 1990): MN 16a (van Dam et al., 2023)

Problematic localities: Port-la-Nouvelle, Castelnou 1: mix of different ages (Freudenthal, Mein & Martín Suárez, 1998)

Observed material: Faculty of Sciences, University Claude Bernard, Lyon (FSL): casts of type material. Bavarian State Collection for Palaeontology and Geology, Munich (SNSB-BSPG): m1, m2, m3, M1, M2, M3 from Nîmes, BSPG 1967 XIV 19–24

Anatomical references: (Hugueney & Mein, 1966; Aguilar, Calvet & Michaux, 1986; Martín Suárez, 1988; Freudenthal, Mein & Martín Suárez, 1998)

Remark: According to Freudenthal et al. (1998), there is *Apocricetus angustidens* in the Spanish locality Vilafant. This locality was related to MN 14 (Gibert, Agustí & Moyà-Solà, 1980), however on the basis that the species is *A. barrierei* (see also Agustí, 1982). As the species identification is not clear, the locality is not included here.

***Apocricetus barrierei*** (Mein & Michaux, 1970)

Occurrences: 41 (in Spain [31] and France [10])

Type locality: Chabrier, France

Holotype: M1 FSL 65356 (figured in Mein and Michaux (1970), pl. 1, fig. 1, as *Cricetus barrierei*)

Age range: 5.47–3.04 Ma (4.255 Ma)

Oldest occurrences: Celleneuve, Chabrier, Hautimagne, Vendargues: MN 14

Youngest occurrence: Huéscar 3 (van Dam et al., 2023)

Problematic localities: Castelnou 1, 3: mix of different ages (Freudenthal, Mein & Martín Suárez, 1998); Almudena 1D: age unknown (Freudenthal, Mein & Martín Suárez, 1998; Ruiz-Sánchez et al., 2014)

Observed material: FSL: cast of holotype & paratypes m1 FSL 65357, m3 FSL 65358 & FSL 65359 M3; cast of M3 and m2 from Hautimagne

Anatomical references: (Mein & Michaux, 1970; Ruiz Bustos et al., 1984; Martín Suárez, 1988; Aguilar et al., 1991; Freudenthal, Mein & Martín Suárez, 1998; Ambert, Aguilar & Michaux, 1998; García-Alix et al., 2008; Ruiz-Sánchez et al., 2014; Mansino et al., 2015; Piñero et al., 2017; Piñero & Agustí, 2020)

Remark: FSL 65359 is from Hautimagne according to Mein and Michaux (1970) but from Chabrier (Type locality) according to the specimen label.

***Apocricetus darderi*** Torres-Roig, Agustí, Bover & Alcover 2019

Occurrence/type locality: Na Burgesa-1, Spain

Holotype: mandible, IMEDEA 94726, Institut Mediterrani d’Estudis Avançats, Esporles (figured in Torres-Roig et al. (2019), figs. 5(A), 7(A), 7(B))

Age: 5.333–3.6 Ma (4.467 Ma): Zanclean (Torres-Roig et al., 2019)

Anatomical reference: (Torres-Roig et al., 2019)

***Apocricetus plinii*** (Freudenthal, Lacomba & Martin Suárez, 1991)

Occurrences: 5 (in Spain [4] and France [1])

Type locality: Crevillente 15, Spain

Holotype: M1, RGM 402 10, Naturalis Biodiversity Center, Leiden (figured in Freudenthal, Lacomba and Martin Suárez, (1991), pl. 4, fig. 1, as *Neocricetodon plinii*)

Age range: 7.67–6.27 Ma (6.97 Ma)

Oldest occurrence: Crevillente 5A (van Dam et al., 2023)

Youngest occurrence: Otura 3 (van Dam et al., 2023)

Problematic locality: Castelnou 1: mix of different ages (Freudenthal, Mein & Martín Suárez, 1998)

Anatomical references: (Freudenthal, Lacomba & Martín Suárez, 1991; Freudenthal, Mein & Martín Suárez, 1998)

***Collimys*** Daxner-Höck, 1972

Of the 7 total species, 2 were not included in the analysis: *Collimys caucasicus* Tesakov, 2017; *Collimys primus* Daxner-Höck, 1972. The 5 species, that are included are:

***Collimys dobosi*** Hír, 2005

Occurrences: 5 (in Hungary [4] and Germany [1])

Type locality: Felsötárkány 3/2, Hungary

Holotype: M1, No. 2003.79, Municipal Museum of Pásztó, Nógrád County Museums (figured in Hír (2005), fig. 1)

Age range: 12.2–11.3 Ma (11.75 Ma)

Oldest occurrence: Felsotárkány 2/3 (Hír, 2006; Hír et al., 2016, 2017)

Youngest occurrence: Hillenlohe (Prieto & Rummel, 2009b, 2016)

Observed material: SNSB-BSPG: 81 isolated molars from Hillenlohe, BSPG 1979 XVIII 1–81

Anatomical references: (Hír, 2004, 2005, 2006; Prieto & Rummel, 2009b; Hír & Kókay, 2010)

***Collimys gudrunae*** Prieto and Rummel, 2009b

Occurrence/type locality: Petersbuch 31, Germany

Holotype: M1, NMA 2007/1/2017, Naturmuseum Augsburg (figured in Prieto and Rummel (2009a), fig. 4 b1, b2)

Age: 13.9–13.8 Ma (13.85 Ma) (Prieto & Rummel, 2009a, 2016)

Anatomical reference: (Prieto & Rummel, 2009a)

***Collimys hiri*** Prieto and Rummel, 2009a

Occurrences: 2 (in Germany)

Type locality: Hammerschmiede 1, Germany

Holotype: m1, BSPG 1973-XIX-182, Bavarian State Collection for Palaeontology and Geology (figured in Prieto and Rummel (2009b), fig. 2a)

Age range: 11.6–11.3 Ma (11.45 Ma)

Oldest occurrence: Hammerschmiede 1 (Prieto & Rummel, 2009b, 2016)

Youngest occurrence: Hammerschmiede 3 (Prieto & Rummel, 2009b, 2016)

Observed material: SNSB-BSPG: type material from Hammerschmiede 1, m1, m2, m3, M1, M2, M3, BSPG 1973 XIX 182, 183, 184, 185, 265, 266; 58 isolated molars from Hammerschmiede 3, BSPG 1980 XXVIII 1–58

Anatomical reference: (Prieto & Rummel, 2009b)

Remark: The dating of the Hammerschmiede locality is based on the levels 4 and 5. Hammerschmiede 1 can be correlated to Hammerschmiede 5 (Kirscher et al., 2016). As a more precise dating of Hammerschmiede 1 and 3 is not available, the range for the locality given in Hír et al. (2016) and Prieto and Rummel (2016) is taken.

***Collimys longidens*** Kälin and Engesser, 2001

Occurrence/type locality: Nebelbergweg, Switzerland

Holotype: m1, Nbw 92, Natural History Museum Basel (NMB) (figured in Kälin and Engesser (2001), fig. 37b)

Age: 11.9–11.3 Ma (11.6 Ma)

Anatomical reference: (Kälin & Engesser, 2001)

Remark: According to Kälin and Engesser (2001), the locality Nebelbergweg is related to early MN 9 but according to Hír and Kókay (2010), it can also be late MN 7/8. The relative age of Nebelbergweg compared to Hammerschmiede is uncertain (Prieto & Rummel, 2009b; Prieto et al., 2014). Because of similarities of both faunas a similar or, following Prieto and Rummel (2016), a slightly older age of Nebelbergweg is assumed.

***Collimys transversus*** Heissig, 1995

Occurrence/type locality: Steinheim am Albuch, Germany

Holotype: M1, Sth. 106 H1, NMB (figured in Heissig (1995), pl. 1, fig. 7 and in Kälin and Engesser (2001), fig. 36e)

Age: 13.9–13.8 (13.85 Ma)

Anatomical references: (Heissig, 1995; Kälin & Engesser, 2001)

Remark: *Collimys transversus* is also reported from Belchatow A, Poland (MN 9) (Garapich, 2002). The material is however low in number and is referred to as *Collimys* sp. by other authors (Prieto et al., 2014).

***Cricetulodon*** Hartenberger, 1965

Of the 7 total species, 1 was not included in the analysis: *Cricetulodon cretensis* (de Bruijn and Meulenkamp, 1972) (see de Bruijn et al., 2012). The 6 species, that are included are:

***Cricetulodon bugesiensis*** Freudenthal, Mein & Martín Suárez, 1998

Occurrences: 4 (in France)

Type locality: Soblay, France

Holotype: m1, FSL 65897, FSL (figured in Freudenthal, Mein and Martín Suárez (1998), pl. 1, fig. 1)

Age range: 9.9–8.75 Ma (9.325 Ma)

Oldest occurrences: Douvre: MN 10 (Freudenthal, Mein & Martín Suárez, 1998; Mein, 1999)

Youngest occurrences: Dionay, Lo Fournas 16-M: MN 10 (Freudenthal, Mein & Martín Suárez, 1998; Mein, 1999; Aguilar et al., 2007)

Observed Material: FSL: holotype m1 FSL 65897, isolated molars from Soblay

Anatomical reference: (Freudenthal, Mein & Martín Suárez, 1998)

Remark: Aguilar, Lazzari, et al. (2007) assumed MN 11 and an age of ca. 9.5 Ma for the locality Lo Fournas 16-M, which is considerably older than usual assumptions for the range of this zone, with a maximum of 8.9 or 8.75 Ma (Hilgen et al., 2012; van Dam et al., 2023). We assume an association to MN 10, equal to the other French localities containing *Cricetulodon bugesiensis* (Mein, 1999).

***Cricetulodon complicidens*** Topachevsky and Skorik, 1992

Occurrence/type locality: Gritsev, Ukraine

Holotype: Maxilla, No. 46, IZAN, Schmalhausen Institute of Zoology of National Academy of Sciences of Ukraine (figured in Topachevsky and Skorik (1992), fig. 4a)

Age: 9.78–9.6 Ma (9.69 Ma) (Topachevsky & Skorik, 1992)

Anatomical reference: (Topachevsky & Skorik, 1992)

Remark: Gritsev is Bessarabien in age, younger than Buzhor 1 and Kalfa and with reversed palaeomagnetism (Pevzner & Vangengeim, 1993; Vangengeim, Lungu & Tesakov, 2006), therefore it is probably correlated with C4Ar.2r or C4Ar.3r: lower border: 9.78 Ma (see Vasiliev et al., 2011; Ogg, 2020), the upper border follows the upper border of the Bessarabian: 9.6 Ma (Palcu et al., 2021).

***Cricetulodon hartenbergeri*** Freudenthal, 1967

Occurrences: 24 (in Spain [22] and Turkey [2])

Type locality: Pedregueras 2C, Spain

Holotype: m1, PEC 585, Institut de Paleontologia, Sabadell (figured in Freudenthal (1967), pl. 1, fig. 14, as *Rotundomys hartenbergeri* and in Erten, Şen & Gormus (2014), fig. 11, 4)

Age range: 10.6–9.49 Ma (10.045 Ma)

Oldest localitiy: Pedregueras 2A (van Dam et al., 2014)

Youngest occurrence: Puente Minero 10 (van Dam et al., 2006)

Observed Material: FSL: isolated molars from Pedregueras 2C. SNSB-BSPG: 2 m1, 2 m2, 2 m3, 2 M1, 2 M2 (broken), 2 M3 from Pedregueras 2C, BSPG 1966 III 91–102

Anatomical references: (Freudenthal, 1967; Agustí et al., 1979; Şen, 1991; Freudenthal, Mein & Martín Suárez, 1998; Pineda-Muñoz et al., 2011; Erten, Şen & Gormus, 2014)

Remark: *Cricetulodon hartenbergeri* was reported from Hostalets de Pierola Inferior (see Alba et al., 2006), which would be an even older location than Pedregueras 2A. The material was however recently assigned to another species, *Democricetodon nemoralis*, which is why this locality is not included here (see SOM S1 in Alba et al., 2022).

***Cricetulodon lucentensis*** Freudenthal, Lacomba & Martín Suárez, 1991

Occurrences: 3 (in Spain)

Type locality: Crevillente 17, Spain

Holotype: m1, RGM 404 67, Naturalis Biodiversity Center, Leiden (figured in Freudenthal, Lacomba and Martín Suárez (1991), pl. 5, fig. 1, as *Neocricetodon lucentensis*)

Age range: 7.17–6.89 Ma (7.03 Ma)

Oldest occurrence: Crevillente 5 (van Dam et al., 2023)

Youngest occurrence: Crevillente 17 (van Dam et al., 2023)

Anatomical references: (Freudenthal, Lacomba & Martín Suárez, 1991; Freudenthal, Mein & Martín Suárez, 1998)

***Cricetulodon meini*** Agustí, 1986

Occurrence/type locality: Casa del Acero, Spain

Holotype: M1, FCA-237, Institut de Paleontologia, Sabadell (figured in Agustí (1986), pl. 2, fig. 8, as *Kowalskia meini*)

Age: 7.58–7.53 Ma (7.555 Ma) (van Dam et al., 2023)

Anatomical references: (Agustí, 1986; Freudenthal, Mein & Martín Suárez, 1998)

Remark: The material from Maramena, Greece described as *Allocricetus* cf. *ehiki* (by Daxner-Höck, 1992), shows similarities to *Cricetulodon meini* (Cuenca Bescós, 2003). With an age of around 5.5 Ma (Hordijk & de Bruijn, 2009) this would be a considerably younger occurrence but due to the uncertainties regarding the taxonomic identification, it is not considered here.

***Cricetulodon sabadellensis*** Hartenberger, 1966

Occurrences: 12 (in Spain)

Type locality: Can Llobateres, Spain

Holotype: maxilla, CL 1392, Institut de Paleontologia, Sabadell (figured in Hartenberger (1965), pl. 10, fig. c)

Age range: 9.98–9.12 Ma (9.55 Ma)

Oldest occurrences: Can Casablanques 1B, Can Coromines 2, Can Pallars de Llobateres (Garcés et al., 1996; Checa Soler & Rius Font, 2003; Casanovas-Vilar et al., 2016)

Youngest occurrence: Torrent de Febulines M (Freudenthal, Mein & Martín Suárez, 1998; Casanovas-Vilar et al., 2014)

Observed specimens: University Montpellier: type material from Can Llobateres

Anatomical references: (Hartenberger, 1965; Freudenthal, Mein & Martín Suárez, 1998)

Remarks: Can Coromines 2 is associated to chron C5n.1r (Agustí et al., 1997), Can Pallars de Llobateres belongs to the *Cricetulodon hartenbergeri* – *Progonomys hispanicus* interval subzone (Casanovas-Vilar et al., 2016). The lower boundaries are shared (9.98 Ma). Can Casablanques 1B is not precisely dated but can be assumed to fall in the same range.
According to Casanovas-Vilar et al. (2016) the *Cricetulodon* species in Autopista de Rubí-Terrassa 8 and 3B, Spain are *Cricetulodon* cf. *hartenbergeri* and *Cricetulodon hartenbergeri*. But according to López-Antoñanzas, Peláez-Campomanes and Álvarez-Sierra (2014), it is *C. sabadellensis*, which would make Autopista de Rubí-Terrassa 8 the oldest occurrence but due to the uncertainties regarding the taxonomic identification, it is not considered here.

***Hattomys*** Freudenthal 1985

All of the 3 total species are included in the analysis:

***Hattomys beetsi*** Freudenthal, 1985

Occurrences: 5 (in Italy)

Type locality: Biancone 1, Italy

Holotype: mandible, RGM 263 775, Naturalis Biodiversity Center, Leiden (figured in Freudenthal (1985), pl. 1, fig. 1)

Age range: 7.02–4.19 Ma (5.605Ma)

Oldest occurrence: Biancone 1 (Freudenthal, 1985; Kälin, 1999; Savorelli, 2013): MN 13

Youngest occurrence: Trefossi 1 or Chiro 19 (Freudenthal, 1985; Savorelli, 2013): MN 13/14

Anatomical references: (Freudenthal, 1985; Savorelli, 2013)

Remark: For all localities of *Hattomys*, an age of MN 13/14 is used (Kälin, 1999; Freudenthal & Martín-Suárez, 2006; Freudenthal & Martín Suárez, 2010).

***Hattomys gargantua*** Freudenthal, 1985

Occurrences: 8 (in Italy)

Type locality: Chiro 2N, Italy: MN 13/14? (Kälin, 1999)

Holotype: cranium with maxilla, RGM 263 55, Naturalis Biodiversity Center, Leiden (figured in Freudenthal (1985), pl. 3, fig. 3 and pl. 5, fig. 10–11)

Age range: 7.02–4.19 Ma (5.605Ma)

Observed material: SNSB-BSPG: mandible with m1, m2, m3 from Monte Gargano, Fissure GP XI, BSPG 2004 IX 4

Anatomical references: (Freudenthal, 1985; Savorelli, 2013)

***Hattomys nazarii*** Freudenthal, 1985

Occurrences: 7 (in Italy)

Type locality: Nazario 2B, Italy: MN 13/14? (Kälin, 1999)

Holotype: m1, RGM 263 574, Naturalis Biodiversity Center, Leiden (figured in Freudenthal (1985), pl. 1, fig. 18)

Age range: 7.02–4.19 Ma (5.605 Ma)

Anatomical references: (Freudenthal, 1985; Savorelli, 2013)

***Neocricetodon*** Schaub, 1934

Sinitsa and Delinschi (2016) list 18 species as *Neocricetodon*. We treat their ‘*Neocricetodon*’ *polgardiensis* (Freudenthal and Kordos, 1989) as *Pseudocricetus* (see Freudenthal, Mein & Martín Suárez, 1998) and include *N. shalaensis* (Qiu and Li, 2016). If *N. lavocati* (Hugueney and Mein, 1965) and *N. seseae* Aguilar, Calvet and Michaux, 1995 are also counted as *Neocricetodon* (see Freudenthal, Mein & Martín Suárez, 1998), as well as *N. mesolophidos* (Wu and Flynn, 2017) (see Xie, Zhang & Li, 2021, 2023), that sums up to a total number of 21 species, of which 7 were not included in the analysis: *N. lavocati*; *N. lii* (Zheng, 1993)*; N. mesolophidos*; *N. schaubi* (Kretzoi, 1951); *N. seseae*; *N. shalaensis*; *N. yinanensis* (Zheng, 1984b). The 14 species included in the analysis are:

***Neocricetodon ambarrensis*** Freudenthal, Mein and Martín-Suárez, 1998

Occurrences: 5 (in France [3] and Spain [2])

Type locality: Ambérieu 2C, France

Holotype: m1, FSL 65907 (figured in Freudenthal, Mein and Martín-Suárez (1998), pl. 3, fig. 1)

Age range: 9.9–8.75 Ma (9.325 Ma)

Oldest occurrence: Ambérieu 1: MN 10 (Freudenthal, Mein & Martín Suárez, 1998; Mein, 1999)

Youngest occurrence: Ambérieu 2A: MN 10 (Freudenthal, Mein & Martín Suárez, 1998; Mein, 1999)

Anatomical references: (Freudenthal, Mein & Martín Suárez, 1998; Sinitsa & Delinschi, 2016)

***Neocricetodon browni*** (Daxner-Höck, 1992)

Occurrences: 4 (in Greece [2] and Moldova [2])

Type locality: Maramena, Greece

Holotype: M1, MAA1 1343, Department Aardwetenschappen, University Utrecht (figured in Daxner-Höck (1992), fig. 18/3, as *Kowalskia browni*)

Age range: 7.68–5.3 Ma (6.49Ma)

Oldest occurrence: Chimishlija (Cimislia) (Lungu & Delinschi, 2008): lower border of MN 12

Youngest occurrence: Maramena (Daxner-Höck, 1992; Hordijk & de Bruijn, 2009)

Anatomical references: (Daxner-Höck, 1992; Sinitsa & Delinschi, 2016; Vasileiadou & Sylvestrou, 2022)

Remark: The material from Maramena is mentioned as ‘*Kowalskia fahlbuschi*’ in van Kolfschoten and van der Meulen (1986) and in de Bruijn (1989).

***Neocricetodon fahlbuschi*** (Bachmayer and Wilson, 1970)

Occurrences: 3 (in Austria, Hungary, and Spain)

Type locality: Kohfidisch, Austria

Holotype: Maxilla, No. 1970/1393, Museum of Natural History, Vienna (NHM) (figured in Bachmayer and Wilson (1970), pl. 11, fig. 57 and in Bachmayer and Wilson (1980), pl. 2, fig. 9, as *Kowalskia fahlbuschi*)

Age range: 9.105–7.6 Ma (8.353 Ma)

Oldest occurrence: Sümeg (Kordos, 1987)

Youngest occurrence: Ronda Oest de Sabadell A1 (Casanovas-Vilar et al., 2016)

Observed material: SNSB-BSPG: 2 m2, m3, M2 from Kohfidisch, BSPG 1972 XXII 1–4

Anatomical references: (Bachmayer & Wilson, 1970, 1978, 1980; Daxner-Höck, 1972, 1992; Kordos, 1987; Freudenthal, Mein & Martín Suárez, 1998; Daxner-Höck & Höck, 2015; Sinitsa & Delinschi, 2016)

Remark: The type material is numbered KO 137 1-157 according to Freudenthal, Mein and Martín Suárez (1998). Different ages of the locality Sümeg are given in several papers: MN 10 (e.g., Kordos, 1987), MN 11 (e.g., Hír & Kókay, 2010) or MN 12 (e.g., Fejfar et al., 1997). Bernor et al. (2003) give an absolute age of 9.7–9.0 Ma. Here, Angelone and Čermák (2015) are followed, who gave an overview of the discussions in the past and placed the locality between MN 10 and MN 11. Following their fig. 1, the age is within chron C4An or C4r (9.105–8.125 Ma, following Ogg (2020)).

***Neocricetodon grangeri*** (Young, 1927)

Occurrences: 13 (in China)

Type locality: Chia yu Tsun, Yushe-YS161, China (following Wu & Flynn, 2017)

Holotype: mandibles, cranium, postcranial fragments, Yu She 173, Uppsala University (Lagrelius coll.) (figured in Daxner-Höck et al. (1996), fig. 16.1 and in Wu and Flynn (2017), fig. 10.2)

Age range: 6.727–4.3 Ma (5.514Ma) (Wu & Flynn, 2017)

Oldest occurrences: Yushe-YS139, 142 (Tedford, Qiu & Ye, 2013; Flynn & Wu, 2017)

Youngest occurrences: Yushe-YS4, 97 (Opdyke, Huang & Tedford, 2013; Wu & Flynn, 2017)

Anatomical references: (Daxner-Höck et al., 1996; Sinitsa & Delinschi, 2016; Wu & Flynn, 2017)

Remark: According to Opdyke et al. (2013), fig. 4.8, the locality Yushe-YS97 is in the Mazegou Formation correlated with C2An.3n: 3.596–3.33 Ma (Ogg, 2020). This age is considerably younger than the remaining localities of *N. grangeri*. Therefore, Tedford, Qiu and Ye (2013), fig. 3.4 and Wu and Flynn (2017), fig. 10.6 are followed, according to whom Yushe-YS97 is close in age to Yushe-YS4. That way, the age of Yushe-YS97, 4.3 Ma, as mentioned in Wu and Flynn (2017), is congruent with the age of Yushe-YS4, as determined by Opdyke et al. (2013), correlated with the base of C3n.1n: 4.3 Ma (Ogg, 2020).

***Neocricetodon hanae*** (Qiu, 1995)

Occurrences: 5 (in China)

Type locality: Lufeng-shihuiba, China

Holotype: M1, V10843, Institute of Vertebrate Paleontology and Paleoanthropology of the Chinese Academy of Sciences, Beijing (IVPP) (figured in Qiu (1995), pl. 1, fig. 1)

Age range: 7.15–6.2 Ma (6.675 Ma)

Ocurrences: Leilao 9903–9906 (Xijun & Zhuding, 2002; Dong & Qi, 2013): 7.15–7.10 Ma

Lufeng-shihuiba (Qiu, 1995; Dong & Qi, 2013): ca. 6.9–6.2 Ma

Anatomical references: (Qiu, 1995; Sinitsa & Delinschi, 2016)

***Neocricetodon intermedius*** (Fejfar, 1970)

Occurrence/type locality: Ivanovce, Slovakia

Holotype: mandible, No. 652376, Súúg, OF ‘Geologische Zentralanstalt’, Prague (figured in Fejfar (1970), fig. 1a, as *Kowalskia intermedia*)

Age: 4.95–3.18 Ma (4.065 Ma) MN 15

Observed material: SNSB-BSPG: cast of 9 isolated molars and a maxilla of ‘collection Prague’ from Ivanovce, 73272, 65125, 65128, 652382, -13, -92, -50, -88, -77, -93

Anatomical references: (Fejfar, 1970; Sinitsa & Delinschi, 2016)

Remarks: De Bruijn (1989) and Koufos (2006) mention ‘*Kowalskia intermedia*’ in Lefkon, Greece. There is however no other reference mentioning this occurrence and between Lefkon (MN 10/11) and the type locality Ivanovce (MN 15) is such a large gap, that Lefkon is not included, here.
The locality Ivanovce is mentioned in several papers, a more precise age of the locality than MN 15 was however not found (Fejfar, 1970, 2001; Kordos, 1987; Sabol, Joniak & Holec, 2004; Fejfar & Sabol, 2004; Fejfar et al., 2016). The boundaries of MN 15 follow Hordijk and de Bruijn (2009) and van Dam et al. (2023).

***Neocricetodon magnus*** (Fahlbusch, 1969)

Occurrences: 6 (in Hungary [4], Italy [1] and Poland [1])

Type locality: Podlesice, Poland

Holotype: m1, MF/823/1, Institute of Systematic and Experimental Zoology, Polish Academy of Sciences, Cracow (figured in Fahlbusch (1969), pl. 17, fig. 1, as *Kowalskia magna*)

Age range: 5.47–3.18 Ma (4.325 Ma)

Oldest occurrence: Osztramos 1b, c, e: MN 14 (Jánossy & Kordos, 1977)

Youngest occurrence: Osztramos 10: MN 15 (Jánossy & Kordos, 1977)

Anatomical references: (Fahlbusch, 1969; Jánossy, 1972; Kordos, 1987; Pradel, 1988; Colombero, Pavia & Carnevale, 2014)

Remark: The occurrence of *N. magnus* in the latest Pliocene localities Zalesiaki 1B and Zamkowa Dolna Cave B is based on a single m1 and on a single m3, respectively (Pradel, 1988). Due to the limited material, these occurrences are not considered here.

***Neocricetodon moldavicus*** (Lungu, 1981)

Occurrences: 2 (in Moldova)

Type locality: Kalfa, Moldova

Holotype: mandible, TSU Caf-2425, Department of Geography, Tiraspol State University, Kishinev (figured in Lungu (1981), pl. 15, figs. 1a, b as ‘*Kowalskia moldavica*’ and in Sinitsa and Delinschi (2016), figs. 2.2, 4.2, 4.1)

Age range: 9.94–9.78 Ma (9.86 Ma)

Occurrences: Buzhor 1, Kalfa (Lungu, 1981; Sinitsa & Delinschi, 2016)

Observed material: FSL: cast of holotype m1–3 TSU Caf-2425 / M1–3

Anatomical references: (Lungu, 1981; Sinitsa & Delinschi, 2016)

Remarks: Buzhor 1, Kalfa are associated with the Bessarabian, probably correlated with C5n1n: 9.94–9.78 Ma (see Vasiliev et al., 2011; Ogg, 2020)
*Neocricetodon moldavicus* is mentioned from two additional localities in Moldova, Hirova (Girovo) and Veveriţa 1 (Veveritsa 1) (Lungu & Rzebik-Kowalska, 2011). This material does however not resemble *N. moldavicus* or any other species of *Neocricetodon*, according to Sinitsa and Delinschi (2016).

***Neocricetodon nestori*** (Engesser, 1989)

Occurrence/type locality: Baccinello V3

Holotype: M2, Bac. 242, NMB (figured in Engesser (1989), fig. 16A, as *Kowalskia nestori*)

Age: 6.7–6.4 Ma (6.55 Ma) (Engesser, 1989; Rook, 2016)

Anatomical references: (Engesser, 1989; Sinitsa & Delinschi, 2016)

Remark: The type locality is given as ‘Podere Santa Croce 1’ (Engesser, 1989; Kälin, 1999), sometimes just the faunal horizon ‘Baccinello V3’ is given (Rook, Harrison & Engesser, 1996; Colombero, Pavia & Carnevale, 2014). The age range follows Rook (2016), where only the horizon is given.

***Neocricetodon occidentalis*** (Aguilar, 1982)

Occurrences: 14 (in Spain)

Type locality: Crevillente 2

Holotype: M1, CR2 63, Department Aardwetenschappen, University Utrecht (figured in de Bruijn et al. (1975), pl. 4, fig. 10, as *Kowalskia fahlbuschi*)

Age range: 8.51–7.62 Ma (8.065 Ma)

Oldest occurrence: Masada Ruea 2 (van Dam et al., 2023)

Youngest occurrence: Concud Barranco de las Calaveras (van Dam et al., 2023)

Anatomical references: (de Bruijn et al., 1975; van de Weerd, 1976; Freudenthal, Lacomba & Martín Suárez, 1991; Freudenthal, Mein & Martín Suárez, 1998; Sinitsa & Delinschi, 2016)

Remark: Freudenthal, Mein and Martín Suárez (1998), also mentioned *Neocricetodon occidentalis* in the considerably older locality Masia del Barbo 2B but expressed reservations due to a limited number of specimens.

***Neocricetodon polonicus*** (Fahlbusch, 1969)

Occurrences: 8 (in Hungary [4], Ukraine [2], France [1] and Poland [1])

Type locality: Podlesice, Poland

Holotype: mandible, MF/822/1, Institute of Systematic and Experimental Zoology, Polish Academy of Sciences, Cracow (figured in Fahlbusch (1969), pl. 16, fig. 1a–c, as *Kowalskia polonica*)

Age range: 5.47–3.18 Ma (4.325 Ma)

Oldest occurrences: Hauterives (Freudenthal, Mein & Martín Suárez, 1998): MN 14, Osztramos 1b, c, e (Jánossy, 1972; Jánossy & Kordos, 1977; Kordos, 1987): MN 14, Podlesice (Fahlbusch, 1969; Nadachowski, 1989; Freudenthal, Mein & Martín Suárez, 1998; Fejfar et al., 2011): MN 14

Youngest occurrence: Osztramos 10: MN 15 (Jánossy & Kordos, 1977)

Observed material: SNSB-BSPG: m1–m3, M1–M3 from Podlesice, BSPG 1976 I 2–7

Anatomical references: (Fahlbusch, 1969; Jánossy, 1972; Kordos, 1987; Pradel, 1988; Freudenthal, Mein & Martín Suárez, 1998; Sinitsa & Delinschi, 2016)

Remarks: The occurrence of *N. polonicus* in the latest Pliocene to Pleistocene localities Zamkowa Dolna Cave B and Rebielice Królewskie 1A is based on one m2 and m3 and on a single m2, respectively (Pradel, 1988). Due to the limited material, these occurrences are not considered here.
There are also 6 additional occurrences in China of *N. neimengensis* and *N. zhengi*, that are synonyms of *N. polonicus* according to Sinitsa and Delinschi (2016). They are not considered here. If they are included, Ertemte 2 might be the oldest occurrence (MN 13) (Wu, 1991).

***Neocricetodon progressus*** (Topachevsky and Skorik, 1992)

Occurrences: 3 (in Ukraine)

Type locality: Novo-Elizavetovka 2, Ukraine (see Sinitsa, 2012)

Holotype: Maxilla, No. 47, IZAN, Schmalhausen Institute of Zoology of National Academy of Sciences of Ukraine (figured in Topachevsky and Skorik (1992), fig. 19, as *Kowalskia progressa*)

Age range: 8.5–7.65 Ma (8.075 Ma)

Oldest occurrence: Palievo (Sinitsa, 2012; Sinitsa & Nesin, 2018)

Youngest occurrences: Maloye, Novo-Elizavetovka 2 (Nesin & Storch, 2004; Sinitsa, 2012): upper MN 11

Anatomical references: (Topachevsky & Skorik, 1992; Sinitsa, 2012; Sinitsa & Delinschi, 2016)

***Neocricetodon similis*** (Wu, 1991)

Occurrences: 3 (in China)

Type locality: Ertemte 2, China

Holotype: M1, V8723.50, IVPP (figured in Wu (1991), pl. 2, fig. 22, as *Kowalskia similis*)

Age range: 6.5–5.3 Ma (6.2 Ma)

Oldest occurrence: Ertemte 2 (Wu, 1991; Qiu, Wang & Li, 2013; Qiu & Li, 2016)

Youngest occurrence: Harr Obo 2 (Wu, 1991; Qiu, Wang & Li, 2013; Qiu & Li, 2016)

Anatomical references: (Wu, 1991; Sinitsa & Delinschi, 2016)

Remark: Qiu and Li (2016) ‘arbitrarily’ assign five molars from Balunhalagen, China and four molars from Bilutu, China to *Neocricetodon similis* (‘*Kowalskia similis*’), due to a close geographic position to Ertemte and Harr Obo. Especially Balunhalagen (up to 11 Ma) is however considerably older than Ertemte and Harr Obo and due to poor material and an arbitrary assignment (Qiu & Li, 2016), these localities are not considered here.

***Neocricetodon skofleki*** (Kordos, 1987)

Occurrences: 8 (in France [4], Austria [2], Hungary [1], and Slovakia [1])

Type locality: Tardosbánya, Hungary

Holotype: mandible, V.86.2, Paleontological Department, Hungarian Natural History Museum, Budapest (figured in Kordos (1987), pl. 1, fig. 1, as *Karstocricetus skofleki*)

Age range: 8.75–6.9 Ma (8.2 Ma)

Oldest occurrence: Amberieu 3: MN11 (Freudenthal, Mein & Martín Suárez, 1998; Mein, 1999)

Youngest occurrences: Tardosbánya, Šalgovce: MN 12 (Kordos, 1987; Sabol et al., 2021)

Observed material: FSL: cast of M1–3, m1–3 from Tardosbánya

Anatomical references: (Daxner-Höck, 1972; Kordos, 1987; Freudenthal, Mein & Martín Suárez, 1998; Daxner-Höck & Höck, 2015; Sinitsa & Delinschi, 2016)

Remark: The material of Dionay and Ambérieu 2C, is only associated to *N. skofleki* with doubt in Freudenthal, Mein and Martín Suárez (1998), while the material of Ambérieu 1 and 2A is not mentioned at all.

***Pseudocricetus*** Topachevsky and Skorik, 1992

Of the 4 total species, 1 was not included in the analysis: *Pseudocricetus antiquus* Topachevsky and Skorik, 1992. The 3 species, that are included are:

***Pseudocricetus kormosi*** (Schaub, 1930)

Occurrences: 5 (in Ukraine [4] and Hungary [1])

Type locality: Polgardi 2, Hungary

Lectotype: mandible, Ob. 4185, Palaeovertebrate Collection of the Hungarian Geological Institute (figured in Freudenthal and Kordos (1989), fig. 8, 1, as *Cricetus kormosi*)

Age range: 7.02–5.6 Ma (6.31 Ma)

Occurrences: Polgardi 2 (Freudenthal & Kordos, 1989), Andreevka (Nesin & Storch, 2004; Sinitsa, 2010), Novoukrainka 1, Orekhova, (Topachevsky & Skorik, 1992; Nesin & Storch, 2004; Sinitsa, 2010), Odessa Pontian Lectostratotype (Topachevsky & Skorik, 1992; Nesin & Storch, 2004)

Observed Material: FSL: cast of type material Ob. 4185 M1–3 & M1–2, M1; originals from NMB & Paleontological Department, Hungarian Natural History Museum, Budapest. SNSB-BSPG: cast of type material, 3 maxilla fragments with M1, M2 and 3 mandible fragments with m1, m2, m3 of the Hungarian Natural History Museum, Budapest, BSPG 1979 I 154

Anatomical references: (de Bruijn et al., 1975; Freudenthal & Kordos, 1989; Topachevsky & Skorik, 1992; Sinitsa, 2010)

Remark: Following Nesin and Storch (2004) all the Ukrainian localities belong to MN 13 and the upper Maeotian/Pontian. Therefore, the lower border of MN 13 of van Dam et al. (2023), and the upper border of the Pontian is taken (following Krijgsman et al., 2010; Vasiliev et al., 2011). The Hungarian locality Polgardi 2 (MN 13, Pontian) is assumed to fall in this range as well (Freudenthal & Kordos, 1989).

***Pseudocricetus orienteuropaeus*** Topachevsky and Skorik, 1992

Occurrences: 7 (in Ukraine [6] and Moldova [1])

Type locality: Protopovka 3, Ukraine (see Sinitsa, 2010)

Holotype: mandible, No. 50, IZAN, Schmalhausen Institute of Zoology of National Academy of Sciences of Ukraine

Age range: 7.68–6.04 Ma (6.86 Ma)

Occurrences: Belka 2; Egorovka 1, 2; Kubanka 2; Novo-Elizavetovka 3; Protopopovka 3 (Nesin & Storch, 2004; Sinitsa, 2010; Rosina & Sinitsa, 2014; Sinitsa & Nesin, 2018), Chimishlija (Cimislia) (Lungu & Delinschi, 2008)

Anatomical references: (Topachevsky & Skorik, 1992; Sinitsa, 2010)

Remark: All the localities belong to MN 12 and the Maeotian. Therefore, the lower border of MN 12 of van Dam et al. (2023), and the upper border of the Maeotian is taken (following Krijgsman et al., 2010; Vasiliev et al., 2011). As there is disagreement about the border of MN12 to MN 13 in the Maeotian, the here used absolute ages overlap.

***Pseudocricetus polgardiensis*** (Freudenthal and Kordos, 1989)

Occurrences: 2 (in Hungary)

Type locality: Polgardi 4, Hungary

Holotype: mandible, V. 14033 (Vt. 126), Palaeovertebrate Collection of the Hungarian Geological Institute (figured in Freudenthal and Kordos (1989), figs. 1, 1; 5)

Age range: 7.02–5.6 Ma (6.31 Ma)

Occurrences: Polgardi 4 (Freudenthal & Kordos, 1989), Polgardi 3 (Mihály, 1975; Freudenthal & Kordos, 1989; Kordos, 2022)

Anatomical reference: (Freudenthal & Kordos, 1989)

Remark: The age is assumed to be MN 13/Pontian (Freudenthal & Kordos, 1989). The age of Polgardi 2 is followed.

***Rotundomys*** Mein, 1965

Of the 6 total species, 1 was not included in the analysis: *Rotundomys mundi* Calvo, Elizaga, López-Martínez, Robles & Usera, 1979. The 5 species, that are included are:

***Rotundomys bressanus*** Mein, 1975

Occurrences: 19 (in Spain [13], France [5], and Santa Margarida [1])

Type locality: Soblay, France

Holotype: M1, FSL 65443

Age range: 9.9–8 Ma (8.95 Ma)

Oldest occurrence: Soblay (Mein, 1999): MN 10

Youngest occurrences: Bernardière (Mein, 1999): Early MN 11

Observed material: FSL: cast of holotype and paratypes

Anatomical references: (Mein, 1975; Topachevsky & Skorik, 1992; Freudenthal, Mein & Martín Suárez, 1998; Fejfar et al., 2011; López-Antoñanzas, Peláez-Campomanes & Álvarez-Sierra, 2014)

Remark: A locality ‘Santa Margarida’ is mentioned with an occurrence of *R. bressanus* with no further comment in Freudenthal, Mein and Martín Suárez (1998). There is a locality with that name from Portugal mentioned in Antunes and Mein (1989), with an age of ca. 6 Ma, which is younger than all other localities of *R. bressanus*. In NOW there is a locality ‘Santa Margarida’ in Spain, associated with MN 5, which is too old for *R. bressanus*. For neither of the two localities, *R. bressanus* is mentioned in the respective references.

***Rotundomys freiriensis*** Antunes and Mein, 1979

Occurrences: 4 (in Portugal [2] and Spain [2])

Type locality: Freiria do Rio Manor, Portugal

Holotype: M1, Department of Earth Sciences, NOVA School of Science and Technology, NOVA University Lisbon (figured in Antunes and Mein (1979), pl. 1, fig. a, i)

Age range: 9.87–9.62 Ma (9.745 Ma)

Occurrences: Asseiceira (Antunes et al., 1992), Freiria do Rio Manor (Antunes & Mein, 1979), Autopista de Rubí-Terrassa 7C, 11 (Casanovas-Vilar et al., 2016)

Observed material: FSL: casts of holotype / paratypes

Anatomical references: (Antunes & Mein, 1979; Antunes et al., 1992; Freudenthal, Mein & Martín Suárez, 1998; López-Antoñanzas, Peláez-Campomanes & Álvarez-Sierra, 2014)

Remark: The localities Autopista de Rubí-Terrassa 7C and 11 are associated to the *Cricetulodon sabadellensis* + *Progonomys hispanicus* concurrent range subzone (= 9.73–9.65 Ma) of Casanovas-Vilar et al. (2016), which falls within the range of the J1 subzone (= 9.87–9.62 Ma) of van Dam et al. (2006), that Asseiceira and Freiria do Rio Manor are correlated to (Mein, 2000).

***Rotundomys intimus*** López-Antoñanzas, Peláez-Campomanes and Álvarez-Sierra, 2014

Occurrences: 4 (in Spain)

Type locality: Cerro de los Batallones 5, Spain

Holotype: mandible, BAT5’10-07, National Museum of Natural Sciences, Madrid (figured in López-Antoñanzas, Peláez-Campomanes and Álvarez-Sierra (2014), fig. 3A)

Age range: 9.71–8.75 Ma (9.23 Ma)

Oldest occurrence: Cerro de los Batallones 10 (López-Antoñanzas et al., 2010; López-Antoñanzas, Peláez-Campomanes & Álvarez-Sierra, 2014; Martín-Perea et al., 2021)

Youngest occurrence: Cerro de los Batallones 3 (López-Antoñanzas et al., 2010; López-Antoñanzas, Peláez-Campomanes & Álvarez-Sierra, 2014; Martín-Perea et al., 2021)

Anatomical reference: (López-Antoñanzas, Peláez-Campomanes & Álvarez-Sierra, 2014)

Remark: The Cerro de los Batallones system is placed in the local subzones J2 or J3 (López-Antoñanzas et al., 2010), so this range is taken from van Dam et al. (2006).

***Rotundomys montisrotundi*** (Schaub, 1944)

Occurrences: 12 (in Spain [7] and France [5])

Type locality: Montredon, France

Holotype: m1, A Mo 849, NMB (figured in Mein (1965), fig. 1a and in Fejfar et al. (2011), fig. 2(19a))

Age range: 9.9–8.75 Ma (9.325 Ma)

Occurrences: Ampudia 3, Camí de Can Tarumbot 2, 3, Can Casablanques, Can Casablanques 2, Chabeuil - Les Bourbons, La Tarumba 1, Lo Fournas 6, 7, 1993, Montredon, Racor (López Martinez, García Moreno & Álvarez-Sierra, 1986; Mein, 1999; Guerra-Merchán, Ramallo & Ruiz Bustos, 2001; Casanovas-Vilar et al., 2016): MN 10

Anatomical references: (Mein, 1965; Aguilar, 1982; Freudenthal, Mein & Martín Suárez, 1998; Guerra-Merchán, Ramallo & Ruiz Bustos, 2001; Aguilar, Michaux & Lazzari, 2007; Fejfar et al., 2011; López-Antoñanzas, Peláez-Campomanes & Álvarez-Sierra, 2014)

***Rotundomys sabatieri*** Aguilar, Michaux and Lazzari, 2007

Occurrences: 2 (in France)

Type locality: Lo Fournas 16-M, France

Holotype: m1, Fou 16-M no 395, Faculty of Science, University of Montpellier (figured in Aguilar, Michaux and Lazzari (2007), pl. 2, fig. 2)

Age range: 9.9–8.75 Ma (9.325 Ma)

Oldest occurrence: Lo Fournas 6 (Aguilar et al., 2007; Aguilar, Michaux & Lazzari, 2007)

Youngest occurrence: Lo Fournas 16-M (Aguilar et al., 2007; Aguilar, Michaux & Lazzari, 2007)

Anatomical references: (Aguilar, Michaux & Lazzari, 2007; López-Antoñanzas, Peláez-Campomanes & Álvarez-Sierra, 2014)

Remark: Both localities are considered MN 10, which fits with the age given for Lo Fournas 16-M (± 9.5 Ma) in Aguilar et al. (2007).

**Extant taxa**

Following Musser and Carleton (2005) and incorporating the results of Lebedev et al. (2018), there are 9 extant genera with 18 extant species. Two of these genera with their single extant species are included in this study. Type localities and the distributions of the taxa follow Musser and Carleton (2005) and references therein.

***Cricetus*** Leske, 1779

The only extant species of the genus is included in the analysis:

***Cricetus cricetus*** (Linnaeus, 1758)

Type locality: Germany

Distribution: Central Europe, Western Siberia, Northern Kazakhstan, North-Western China

Anatomical references: (Fahlbusch, 1976; Pradel, 1985; Hír, 1997)

Observed material: SNSB-BSPG: 95 skulls with mandibles from Rhine Hesse, Germany (‘Raum Alsheim, Wintersheim, Dorn Dürkheim’), BSPG 1991 IV 1–95

***Nothocricetulus*** Lebedev, Bannikova, Neumann, Ushakova, Ivanova and Surov, 2018

Based on molecular data, Lebedev et al. (2018) erected a new genus, *Nothocricetulus*, for the former ‘*Cricetulus*’ *migratorius* (see also Neumann et al., 2006). This only species of the genus is included in the analysis:

***Nothocricetulus migratorius*** (Pallas, 1773)

Type locality: Western Kazakhstan, lower Ural River

Distribution: Greece, Romania, Bulgaria, Southern European Russia, Kazakhstan, Southern Mongolia, Northern China, Turkey, Israel, Jordan, Lebanon, Iraq, Iran, Afghanistan, Northern India

Anatomical references: (Pradel, 1981; Topachevsky & Skorik, 1992; Hír, 1993; Peshev, Peshev & Popov, 2004; Kryštufek & Vohralík, 2009; Nedyalkov, 2016; Lebedev et al., 2018; Xie, Zhang & Li, 2023)

Observed material: SNSB-BSPG: 21 mandibles, 3 maxilla fragments from the surroundings of Kabul, BSPG 1977 VI

**References**

Aguilar J-P. 1982. Contributions à l’étude des micromammifères du gisement Miocène supérieur de Montredon (Hérault). 2- Les rongeurs. *Palaeovertebrata* 12:81–117.

Aguilar J-P, Calvet M, Michaux J. 1986. Description des rongeurs Pliocènes de la faune du Mont-Hélène (Pyrénées-Orientales, France), nouveau jalon entre les faunes de Perpignan (Serrat-d’En-Vaquer) et de Sète. *Palaeovertebrata* 16:127–144.

Aguilar J-P, Calvet M, Michaux J. 1995. Les rongeurs du gisement karstique miocène supérieur de Caltelnou 1 (Pyrénées-Orientales, France). *Geobios* 28:501–510.

Aguilar J-P, Lazzari V, Michaux J, Sabatier M, Calvet M. 2007. Lo Fournas 16-M (Upper Miocene) and Lo Fournas 16-P (middle Pliocene), news karstic localities to Baixas, Southern France Part. I - Description and geodynamic implications. *Géologie de la France*:55–62.

Aguilar J-P, Michaux J, Bachelet B, Calvet M, Faillat J-P. 1991. Les nouvelles faunes de rongeurs proches de la limite mio-pliocène en Roussillon. Implications biostratigraphiques et biogéographiques. *Palaeovertebrata* 20:147–174.

Aguilar J-P, Michaux J, Lazzari V. 2007. Lo Fournas 16-M (Upper Miocene) and Lo Fournas 16-P (middle Pliocene), news karstic localities to Baixas, Southern France Part. II - New rodens, faunal list, and comment on the use of karstic faunas in biochronology. *Geologie de la France*:63–81.

Agustí J. 1982. Bizonación del neogeno continental de Cataluña mediante roedores (Mammalia). *Acta geologica hispanica* 17:21–26.

Agustí J. 1986. Nouvelles espèces de Cricetidés vicariantes dans le Turolien moyen de Fortuna (Prov. Murcia, Espagne). *Geobios* 19:5–16.

Agustí J, Cabrera L, Garcés M, Parés JM. 1997. The Vallesian mammal succession in the Vallès-Penedès basin (northeast Spain): Paleomagnetic calibration and correlation with global events. *Palaeogeography, palaeoclimatology, palaeoecology* 133:149–180.

Agustí J, Gibert J, Moyà S, Cabrera L. 1979. Roedores e insectívoros (Mammalia) del Mioceno superior de la Seu d’Urgell (Cataluña, España). *Acta Geòlogica Hispànica* 14:362–369.

Alba DM, Moyà-Solà S, Casanovas-Vilar I, Galindo J, Robles JM, Rotgers C, Furió M, Angelone C, Köhler M, Garcés M, Others. 2006. Los vertebrados fósiles del Abocador de Can Mata (els Hostalets de Pierola, l’Anoia, Cataluña), una sucesión de localidades del Aragoniense superior (MN6 y MN7+ 8) de la cuenca del Vallès-Penedès. Campañas 2002-2003, 2004 y 2005. *Estudios*  62:295–312.

Alba DM, Robles JM, Casanovas-Vilar I, Beamud E, Bernor RL, Cirilli O, DeMiguel D, Galindo J, Llopart I, Pons-Monjo G, Sánchez IM, Vinuesa V, Garcés M. 2022. A revised (earliest Vallesian) age for the hominoid-bearing locality of Can Mata 1 based on new magnetostratigraphic and biostratigraphic data from Abocador de Can Mata (Vallès-Penedès Basin, NE Iberian Peninsula). *Journal of human evolution* 170:103237.

Ambert P, Aguilar J-P, Michaux J. 1998. Évolution géodynamique messino-pliocène en Languedoc central: le paléo-réseau hydrographique de l’Orb et de l’Hérault (sud de la France). *Geodinamica Acta* 11:139–146.

Angelone C, Čermák S. 2015. Two new species of *Prolagus* (Lagomorpha, Mammalia) from the Late Miocene of Hungary: taxonomy, biochronology, and palaeobiogeography. *Paläontologische Zeitschrift* 89:1023–1038.

Antunes MT, Mein P. 1979. Le gisement de Freiria de Rio Maior, Portugal, et sa faune de Mammiferes; Nouvelle espece de *Rotundomys*, consequences stratigraphiques. *Geobios* 12:913–919.

Antunes MT, Mein P. 1989. Small mammals of late Miocene from Alvalade basin (Portugal); comparison with faunas from Spain and Maghreb. *Bollettino della Società Paleontologica Italiana* 28:161–170.

Antunes MT, Soulié-Märsche I, Mein P, Pais J. 1992. Le gisement de Asseiceira, Portugal (Miocène supérieur) Données complémentaires sur Freiria de Rio Maior Données complémentaires sur Freiria de Rio Maior. *Ciências da Terra (UNL)* 11:219–253.

Bachmayer F, Wilson RW. 1970. Small Mammals (Insectivora, Chiroptera, Lagomorpha, Rodentia) from the Kohfidisch Fissures of Burgenland, Austria. *Annalen des Naturhistorischen Museums in Wien* 74:533–587.

Bachmayer F, Wilson RW. 1978. A second Contribution to the Small Mammal Fauna of Kohfidisch, Austria / Zweiter Beitrag zur Kleinsäugerfauna von Kohfidisch (Burgenland, Österreich). *Annalen des Naturhistorischen Museums in Wien* 81:129–161.

Bachmayer F, Wilson RW. 1980. A third contribution to the fossil small mammal fauna of Kohfidisch (Burgenland), Austria. *Annalen des Naturhistorischen Museums in Wien* 83:351–386.

Bernor RL, Scott RS, Fortelius M, Şen Ş. 2003. Equidae (Perissodactyla). In: Fortelius M, Kappelman J, Şen Ş, Bernor RL eds. *The Geology and Paleontology of the Miocene Sinap Formation, Turkey*. New York City: Columbia University Press, 220–281.

Biquand D, Dubar M, Sémah F. 1990. Paleomagnetic correlation of the Mediterranean upper Neogene biochronology and Villafranchian vertebrate sites of the Massif Central, France. *Quaternary Research* 33:241–252.

de Bruijn H. 1989. Smaller mammals from the upper Miocene and lower Pliocene of the Strimon basin, Greece. I : Rodentia and Lagomorpha. *Bollettino della Società Paleontologica Italiana* 28:189–195.

de Bruijn H, Doukas CS, van den Hoek Ostende LW, Zachariasse WJ. 2012. New finds of rodents and insectivores from the Upper Miocene at Plakias (Crete, Greece). *Swiss Journal of Palaeontology* 131:61–75.

de Bruijn H, Mein P, Montenat C, van de Weerd AA. 1975. Correlations entre les gisements de rongeurs et les formations marines du Miocène terminal d’Espagne méridionale (prov. de Alicante et Murcia). *Proceedings of the Koninklijke Nederlandse Akademie van Wetenschappen. Series B* 78:282–283.

Calvo JP, Elizaga E, López Martínez N, Robles F, Usera J. 1979. El Mioceno superior continental del Prebético Externo: Evolución del Estrecho Nordbético. *Bol. Inst. Geol. Mi Espana* 89:407–426.

Casanovas-Vilar I, Garcés M, van Dam JA, García-Paredes I, Robles JM, Alba DM, Others. 2016. An updated biostratigraphy for the late Aragonian and Vallesian of the Vallès-Penedès Basin (Catalonia). *Geologica Acta* 14:195–217.

Casanovas-Vilar I, van den Hoek Ostende LW, Furió M, Madern PA. 2014. The range and extent of the Vallesian Crisis (Late Miocene): new prospects based on the micromammal record from the Vallès-Penedès basin (Catalonia, Spain). *Journal of Iberian Geology* 40:29–48.

Checa Soler L, Rius Font L. 2003. Intervenció paleontològica a l’ EDAR Sabadell-Riu Ripoll (Can Llobateres, Sabadell, Vallès Occidental). *Tribuna d’Arqueologia* 1999–2000:7–22.

Colombero S, Pavia G, Carnevale G. 2014. Messinian rodents from Moncucco Torinese, NW Italy: palaeobiodiversity and biochronology. *Geodiversitas* 36:421–475.

Cuenca Bescós G. 2003. *Allocricetus* (Cricetidae, Rodentia, Mammalia) from the Pleistocene. A phylogenetical approach. *Coloquios de Paleontología* 1:95–113.

van Dam JA, Abdul Aziz H, Álvarez-Sierra MÁ, Hilgen FJ, van den Hoek Ostende LW, Lourens LJ, Mein P, van der Meulen AJ, Pelaez-Campomanes P. 2006. Long-period astronomical forcing of mammal turnover. *Nature* 443:687–691.

van Dam JA, Krijgsman W, Abels HA, Álvarez-Sierra MÁ, García-Paredes I, López-Guerrero P, Peláez-Campomanes P, Ventra D. 2014. Updated chronology for Middle to Late Miocene mammal sites of the Daroca area (Calatayud-Montalbán Basin, Spain). *Geobios* 47:325–334.

van Dam JA, Mein P, Garcés M, van Balen RT, Furió M, Alcalá L. 2023. A new rodent chronology for the late Neogene of Spain. *Geobios* 76:53–74.

Daxner-Höck G. 1972. Cricetinae aus dem Alt-Pliozän vom Eichkogel bei Mödling (Niederösterreich) und von Vösendorf bei Wien. *Paläontologische Zeitschrift* 46:133–150.

Daxner-Höck G. 1992. Die Cricetinae aus dem Obermiozän von Maramena (Mazedonien, Nordgriechenland). *Paläontologische Zeitschrift* 66:331–367.

Daxner-Höck G, Fahlbusch V, Kordos L, Wu W. 1996. The Late Neogene cricetid genera *Neocricetodon* and *Kowalskia*. In: Bernor RL, Fahlbusch V, Mittmann HW eds. *The Evolution of Western Eurasian Neogene Mammal Faunas*. New York City: Columbia University Press, 220–226.

Daxner-Höck G, Höck E. 2015. *Rodentia neogenica*. Vienna: Verlag der Österreichischen Akademie der Wissenschaften.

Depéret C. 1890-1895. Les animaux Pliocènes du Roussillon. *Mémoires de la Société géologique de France Paléontologie*:1–194.

Dong W, Qi G-Q. 2013. Chapter 11. Hominoid- Producing Localities and Biostratigraphy in Yunnan. In: Wang X, Flynn LJ, Fortelius M eds. *Fossil Mammals of Asia*. New York: Columbia University Press, 293–313.

Engesser B. 1989. The late Tertiary small mammals of the Maremma region (Tuscany, Italy). 2nd part: Muridae and Cricetidae (Rodentia, Mammalia). *Bollettino della Società Paleontologica Italiana* 28:227–252.

Erten H, Şen Ş, Gormus M. 2014. Middle and Late Miocene Cricetidae (Rodentia, Mammalia) from Denizli Basin (Southwestern Turkey) and a New Species of Megacricetodon. *Journal of Paleontology* 88:504–518.

Fahlbusch V. 1969. Pliozäne und Pleistozäne Cricetinae (Rodentia, Mammalia) aus Polen. *Acta Zoologica Cracoviensa* 14:99–138.

Fahlbusch V. 1976. *Cricetus major* WOLDŘICH (Mammalia, Rodentia) aus der mittelpleistozänen Spaltenfüllung Petersbuch 1. *Mitteilungen der Bayerischen Staatssammlung für Paläontologie und historische Geologie* 16:71–81.

Fejfar O. 1970. Die plio-pleistozänen Wirbeltierfaunen von Hajnacka und Ivanovce (Slowakei, CSSR) VI. Cricetidae (Rodentia, Mammalia). *Mitteilungen der Bayerischen Staatssammlung für Paläontologie und Historische Geologie* 10:277–296.

Fejfar O. 2001. The Arvicolids from Arondelli-Triversa: a new look. *Bollettino della Società Paleontologica Italiana* 40:185–193.

Fejfar O, Heinrich W-D, Kordos L, Maul LC. 2011. Microtoid cricetids and the early history of arvicolids (Mammalia, Rodentia). *Palaeontologia Electronica* 14:1–38.

Fejfar O, Heinrich W-D, Pevzner MA, Vangengeim EA. 1997. Late Cenozoic sequences of mammalian sites in Eurasia: an updated correlation. *Palaeogeography, palaeoclimatology, palaeoecology* 133:259–288.

Fejfar O, Heintz É, Ďurišová A, Sabol M. 2016. Pliocene vertebrates from Ivanovce and Hajnáčka (Slovakia). X. Cervidae. *Neues Jahrbuch für Geologie und Paläontologie - Abhandlungen* 281:1–33.

Fejfar O, Sabol M. 2004. Pliocene Carnivores (Carnivora, Mammalia) from Ivanovce and Hajnáčka (Slovakia). *Courier Forschungsinstitut Senckenberg* 246:15–53.

Flynn LJ, Wu W-Y. 2017. Small Mammal Exploration in Yushe Basin, Shanxi Province. In: J. Flynn L, Wu W-Y eds. *Late Cenozoic Yushe Basin, Shanxi Province, China: Geology and Fossil Mammals: Volume II: Small Mammal Fossils of Yushe Basin*. Vertebrate Paleobiology and Paleoanthropology . Dordrecht: Springer Netherlands, 1–10.

Freudenthal M. 1967. On the mammalian fauna of the *Hipparion*-beds in the Calatayud-Teruel Basin (prov. Zaragoza, Spain). Part 3: *Democricetodon* and *Rotundomys* (Rodentia). *Proceedings of the Koninklijke Nederlandse Akademie van Wetenschappen. Series B* 70:298–315.

Freudenthal M. 1985. Cricetidae (Rodentia) from the Neogene of Gargano (Prov. of Foggia, Italy). *Scripta geologica* 77:29–76.

Freudenthal M, Kordos L. 1989. *Cricetus polgardiensis* sp. nov. and *Cricetus kormosi* Schaub, 1930 from the Late Miocene Polgárdi localities (Hungary). *Scripta geologica* 89:71–100.

Freudenthal M, Lacomba JI, Martín Suárez E. 1991. The Cricetidae (Mammalia, Rodentia) from the Late Miocene of Crevillente (prov. Alicante, Spain). *Scripta geologica* 96:9–46.

Freudenthal M, Martín Suárez E. 2010. The age of immigration of the vertebrate faunas found at Gargano (Apulia, Italy) and Scontrone (l’Aquila, Italy). *Comptes Rendus Palevol* 9:95–100.

Freudenthal M, Martín-Suárez E. 2006. Gliridae (Rodentia, Mammalia) from the Late Miocene Fissure Filling Biancone 1 (Gargano, Province of Foggia, Italy). *Palaeontologia electronica*  9:1–23.

Freudenthal M, Mein P, Martín Suárez E. 1998. Revision of Late Miocene and Pliocene Cricetinae (Rodentia, Mammalia) from Spain and France. *Treballs del Museu de Geologia de Barcelona* 7:11–93.

Garapich A. 2002. An overview of Miocene rodents from Bełchatów (Poland). *Folia Zoologica* 51:59–66.

Garcés M, Agustí J, Cabrera L, Parés JM. 1996. Magnetostratigraphy of the Vallesian (late Miocene) in the Vallès-Penedès Basin (northeast Spain). *Earth and planetary science letters* 142:381–396.

García-Alix A, Minwer-Barakat R, Martín Suárez E, Freudenthal M. 2008. Cricetidae and Gliridae (Rodentia, Mammalia) from the Miocene and Pliocene of southern Spain. *Scripta Geologica* 136:1–38.

Gibert J, Agustí J, Moyà-Solà S. 1980. Nuevos datos sobre la bioestratigrafía del Ampurdán. *Boletín Geológico y Minero* 91:705–712.

Guerin C, Mein P, Truc G. 1970. Nouveaux mammifères et mollusques continentaux d’âge pliocène terminal au toit du plateau de Valensole (Alpes de Haute Provence). *Comptes Rendus des Séances de l’Académie des Sciences de Paris, D* 271:2094–2097.

Guerra-Merchán A, Ramallo D, Ruiz Bustos A. 2001. New data on the Upper Miocene micromammals of the Betic Cordillera and their interest for marine-continental correlations. *Geobios* 34:85–90.

Hartenberger JL. 1965. Les Cricetidae (Rodentia) de Can Llobateres (Néogène d’Espagne). *Bulletin de la Société Géologique de France* S7-VII:487–498.

Heissig K. 1995. Die Entwicklung der großen *Democricetodon*-Arten und die Gattung *Collimys* (Cricetidae, Mamm.) im späten Mittelmiozän. *Mitteilungen der Bayerischen Staatssammlung für Paläontologie und historische Geologie* 35:87–108.

Hilgen FJ, Lourens LJ, van Dam JA, Beu AG, Boyes AF, Cooper RA, Krijgsman W, Ogg JG, Piller WE, Wilson DS. 2012. Chapter 29 - The Neogene Period. In: Gradstein FM, Ogg JG, Schmitz MD, Ogg GM eds. *The Geologic Time Scale*. Boston: Elsevier, 923–978.

Hír J. 1993. *Cricetulus migratorius* (PALLAS 1773) (Rodentia, Mammalia) population from the Toros Mountains (Turkey) (With a special reference to the relation of *Cricetulus* and *Allocricetus* genera). *Folia Historico Naturalia Musei Matraensis* 18:17–34.

Hír J. 1997. A Comparative study on the dental morphology of the Early Pleistocene *Cricetus praeglacialis* SCHAUB, 1930 and recent Hungarian *Cricetus cricetus* L. *Folia Historico Naturalia Musei Matraensis* 22:51–79.

Hír J. 2004. The Middle Miocene (Late Astaracian, MN7-8) rodent fauna of Felsötárkány 3/2 (Hungary). *Acta Paleontologica Romaniae* 4:125–136.

Hír J. 2005. *Collimys dobosi* n. sp. (Cricetidae, Mammalia) from the Late Astaracian (MN 8) vertebrate fauna of Felsőtárkány 3/2 (NorthernHungary). *Fragmenta Palaeontologica Hungarica* 23:5–18.

Hír J. 2006. Late Astaracian (Late Sarmatian) Lagomorphs and Rodents from Felsotarkany-Felnemet (Northern Hungary). *Beiträge zur Paläontologie* 30:155–173.

Hír J, Kókay J. 2010. A systematic study of the middle-late Miocene rodents and lagomorphs (Mammalia) of Felsőtárkány 3/8 and 3/10 (Northern Hungary) with stratigraphical relations. *Geodiversitas* 32:307–329.

Hír J, Venczel M, Codrea V, Angelone C, van den Hoek Ostende LW, Kirscher U, Prieto J. 2016. Badenian and Sarmatian s.str. from the Carpathian area: Overview and ongoing research on Hungarian and Romanian small vertebrate evolution. *Comptes rendus. Palevol* 15:863–875.

Hír J, Venczel M, Codrea V, Rössner GE, Angelone C, van den Hoek Ostende LW, Rosina VV, Kirscher U, Prieto J. 2017. Badenian and Sarmatian s.str. from the Carpathian area: Taxonomical notes concerning the Hungarian and Romanian small vertebrates and report on the ruminants from the Felsőtárkány Basin. *Comptes rendus. Palevol* 16:312–332.

Hordijk K, de Bruijn H. 2009. The succession of rodent faunas from the Mio/Pliocene lacustrine deposits of the Florina-Ptolemais-Servia Basin (Greece). *Hellenic Journal of Geosciences* 44:21–103.

Hugueney M, Mein P. 1965. Lagomorphes et rongeurs du Néogène de Lissieu (Rhône). *Travaux et Documents des Laboratoires de Géologie de Lyon* 12:109–124.

Hugueney M, Mein P. 1966. Les Rongeurs pliocènes du Roussillon, dans les collections lyonnaises. *Travaux et Documents des Laboratoires de Géologie de Lyon Année* 13:243–266.

Jánossy D. 1972. Middle Pliocene Microvertebrate Fauna from the Osztramos Loc. 1. (Northern Hungary). *Annales Historico-Naturales Musei Nationalis Hungarici* 64:27–52.

Jánossy D, Kordos L. 1977. The faunistical and karst-morphological review of paleontological localities for vertebrates at Osztramos (Northern Hungary). *Fragmenta Mineralogica et Palaentologica* 8:39–72.

Kälin D. 1999. Tribe Cricetini. In: Rössner GE, Kurt H eds. *The Miocene Land Mammals of Europe*. Munich: Verlag Dr. Friedrich Pfeil, 373–387.

Kälin D, Engesser B. 2001. Die jungmiozäne Säugetierfauna vom Nebelbergweg bei Nunningen (Kanton Solothurn, Schweiz). *Schweizerische Paläontologische Abhandlungen* 121:1–61.

Kirscher U, Prieto J, Bachtadse V, Aziz HA, Doppler G, Hagmaier M, Böhme M. 2016. A biochronologic tie-point for the base of the Tortonian stage in European terrestrial settings: Magnetostratigraphy of the topmost Upper Freshwater Molasse sediments of the North Alpine Foreland Basin in Bavaria (Germany). *Newsletters on stratigraphy* 49:445–467.

Kordos L. 1987. *Karstocricetus skofleki* gen. n., sp. n. and the evolution of the Late Neogene Cricetidae in the Carpathian Basin. *Fragmenta Mineralogica et Palaeontologica* 13:65–88.

Kordos L. 2022. Sedimentologic, Taphonomic Processes and Paleontological Values of Hungarian Caves. In: Veress M, Leél-Őssy S eds. *Cave and Karst Systems of Hungary*. Cham: Springer International Publishing, 487–512.

Koufos GD. 2006. The Neogene mammal localities of Greece: faunas, chronology and biostratigraphy. *Hellenic Journal of Geosciences* 41:183–214.

Krijgsman W, Stoica M, Vasiliev I, Popov VV. 2010. Rise and fall of the Paratethys Sea during the Messinian Salinity Crisis. *Earth and planetary science letters* 290:183–191.

Kryštufek B, Vohralík V. 2009. *Mammals of Turkey and Cyprus: Rodentia II: Cricetinae, Muridae, Spalacidae, Calomyscidae, Capromyidae, Hystricidae, Castoridae*. Koper: Znanstveno-raziskovalno središče Republike Slovenije.

Lebedev VS, Bannikova AA, Neumann K, Ushakova MV, Ivanova NV, Surov AV. 2018. Molecular phylogenetics and taxonomy of dwarf hamsters *Cricetulus* Milne-Edwards, 1867 (Cricetidae, Rodentia): description of a new genus and reinstatement of another. *Zootaxa* 4387:331–349.

Leske NG. 1779. *Algemeine Natur- und Tiergeschichte*. Leipzig: Siegfried Lebrecht Crusius.

Linnaeus C. 1758. *Systema naturae per regna tria naturae, secundum classis, ordines, genera, species cum characteribus, differentiis, synonymis, locis*. Stockholm: Laurentii Salvii.

López Martinez N, García Moreno E, Álvarez-Sierra MÁ. 1986. Paleontología y Bioestratigrafía (micromamíferos) del Mioceno Medio y Superior del sector central de la Cuenca del Duero. *Studia Geológica Salmanticensia* 22:191–212.

López-Antoñanzas R, Peláez-Campomanes P, Álvarez-Sierra MÁ. 2014. New species of *Rotundomys* (Cricetinae) from the Late Miocene of Spain and its bearing on the phylogeny of *Cricetulodon* and *Rotundomys*. *PloS one* 9:e112704.

López-Antoñanzas R, Peláez-Campomanes P, Álvarez-Sierra MÁ, García-Paredes I. 2010. New species of *Hispanomys* (Rodentia, Cricetodontinae) from the Upper Miocene of Batallones (Madrid, Spain). *Zoological journal of the Linnean Society* 160:725–747.

Lungu AN. 1981. *The Hipparion fauna of the Middle Sarmatian of Moldavia (Insectivora, Lagomorpha, Rodentia)*. Chișinău: Shtiintsa Press.

Lungu AN, Delinschi A. 2008. Les particularites des orictocénoses de la faune de *Hipparion* du site Cimişlia. *Acta Palaeontologica Romaniae* 6:187–193.

Lungu AN, Rzebik-Kowalska B. 2011. *Faunal assemblages, stratigraphy and taphonomy of the Late Miocene localities in the Republic of Moldova*. Kraków: Institute of Systematics and Evolution of Animals, Polish Academy of Sciences.

Mansino S, Fierro I, Montoya P, Ruiz-Sánchez FJ. 2015. Micromammal faunas from the Mio-Pliocene boundary in the Alcoy Basin (SE Spain): biostratigraphical and palaeoecological inferences. *Bulletin of Geosciences* 90:555–576.

Mansino S, Ruiz-Sánchez FJ, Freudenthal M, Montoya P. 2014. A new approach to the Late Miocene-Early Pliocene forms of the genus *Apocricetus*. *Apocricetus alberti* (Rodentia, Mammalia) from Venta del Moro (Cabriel Basin, Spain). *Proceedings of the Geologists’ Association* 125:392–405.

Martín Suárez E. 1988. Sucesiones de micromamíferos en la depresión Guadix-Baza (Granada, España). PhD Thesis. Universidad de Granada.

Martín-Perea DM, Morales J, Cantero E, Courtenay LA, Hernández Fernández M, Domingo MS. 2021. Taphonomic analysis of Batallones-10, a Late Miocene drought-induced mammalian assemblage (Madrid basin, Spain) within the Cerro de los Batallones complex. *Palaeogeography, palaeoclimatology, palaeoecology* 578:110576.

Mein P. 1965. *Rotundomys*, nouveau genre de Cricetidae (Mammalia, Rodentia) de la faune néogène de Montredon (Herault). *Bulletin de la Société Géologique de France* S7-VII:421–425.

Mein P. 1975. Une forme de transition entre deux familles de rongeurs. *Actes du Colloque international du Centre National de la Recherche Scientifique* 218:759–763.

Mein P. 1999. The Late Miocene small mammal succession from France, with emphasis on the Rhône Valley localities. In: Agustí J, Rook L, Andrews P eds. *Hominoid Evolution and Climatic Change in Europe*. Cambridge: Cambridge University Press, 140–164.

Mein P. 2000. La biochronologie des Mammifères néogènes d’Europe L’échelle MN, son application à la successiou des faunes du Portugal. *Ciências de Terra (UNL)* 14:335–342.

Mein P, Michaux J. 1970. Un nouveau stade dans l’évolution des rongeurs pliocènes de l’Europe sud-occidentale. *Comptes Rendus des Séances de l’Académie des Sciences de Paris, D* 270:2780–2783.

van der Meulen AJ, van Kolfschoten T. 1986. Review of the Late Turolian to Early Biharian mammal faunas from Greece and Turkey. *Memorie della Societa Geologica Italiana* 31:201–211.

Mihály S. 1975. A new occurrence of Pikermi-type Vertebrata in the big quarry of Polgárdi-Ipartelepek, Transdanubia, Hungary. *Discuss Palaeont* 22:89–93.

Minwer-Barakat R, García-Alix A, Agustí J, Suárez EM, Freudenthal M. 2009. The micromammal fauna from Negratín-1 (Guadix Basin, southern Spain): new evidence of African-Iberian mammal exchanges during the Late Miocene. *Journal of Paleontology* 83:854–879.

Musser GG, Carleton MD. 2005. Superfamily Muroidea. In: Wilson DE, Reeder DM eds. *Mammal Species of the World: A Taxonomic and Geographic Reference*. Baltimore: Johns Hopkins University Press, 894–1531.

Nadachowski A. 1989. Gryzonie - Rodentia. *Folia Quaternaria* 59–60:151–176.

Nedyalkov N. 2016. Distribution and current status of *Cricetulus migratorius* (Mammalia: Cricetidae) in Bulgaria, with comments on its status in the Balkans. *Turkish journal of zoology* 40:925–932.

Nesin VA, Storch G. 2004. Neogene Murinae of Ukraine (Mammalia, Rodentia). *Senckenbergiana Lethaea* 84:351–365.

Neumann K, Michaux J, Lebedev VS, Yigit N, Colak E, Ivanova N, Poltoraus A, Surov AV, Markov G, Maak S, Neumann S, Gattermann R. 2006. Molecular phylogeny of the Cricetinae subfamily based on the mitochondrial cytochrome b and 12S rRNA genes and the nuclear vWF gene. *Molecular phylogenetics and evolution* 39:135–148.

Ogg JG. 2020. Chapter 5 - Geomagnetic Polarity Time Scale. In: Gradstein FM, Ogg JG, Schmitz MD, Ogg GM eds. *Geologic Time Scale 2020*. Boston: Elsevier, 159–192.

Opdyke ND, Huang K, Tedford RH. 2013. The Paleomagnetism and Magnetic Stratigraphy of the Late Cenozoic Sediments of the Yushe Basin, Shanxi Province, China. In: Tedford RH, Qiu Z-X, Flynn LJ eds. *Late Cenozoic Yushe Basin, Shanxi Province, China: Geology and Fossil Mammals: Volume I:History, Geology, and Magnetostratigraphy*. Vertebrate Paleobiology and Paleoanthropology . Dordrecht: Springer Netherlands, 69–78.

Palcu DV, Patina IS, Șandric I, Lazarev S, Vasiliev I, Stoica M, Krijgsman W. 2021. Late Miocene megalake regressions in Eurasia. *Scientific reports* 11:11471.

Pallas PS. 1773. *Reise durch verschiedene Provinzen des Russischen Reichs*. St. Petersburg: Kaiserliche Akademie der Wissenschaften.

Peshev TH, Peshev DT, Popov VV. 2004. *Fauna Bulgarica 27: Mammalia*. Sofia: Marin Drinov.

Pevzner MA, Vangengeim EA. 1993. Magnetochronological age assignments of Middle and Late Sarmatian mammals of the Eastern Paratethys. *Newsletters on Stratigraphy* 29:63–75.

Pineda-Muñoz S, Casanovas-Vilar I, Alba DM, Carmona R, Rifà E. 2011. Cricetinae (Rodentia) from site B40/ S5C (Vallès-Penedès Basin, NE Iberian Peninsula). Biostratigraphic implications. In: Pérez-García A, Gascó F, Gasulla JM, Escaso F eds. *Viajando a mundos pretéritos*. Morella: Ayuntamiento de Morella, 289–296.

Piñero P, Agustí J. 2019. The rodent succession in the Sifón de Librilla section (Fortuna Basin, SE Spain): implications for the Mio-Pliocene boundary in the Mediterranean terrestrial record. *Historical biology* 31:279–321.

Piñero P, Agustí J. 2020. Rodents from Botardo-D and the Miocene-Pliocene transition in the Guadix-Baza Basin (Granada, Spain). *Palaeobiodiversity and Palaeoenvironments* 100:903–920.

Piñero P, Agustí J, Oms O, Blain H-A, Laplana C, Ros-Montoya S, Martínez-Navarro B. 2017. Rodents from Baza-1 (Guadix-Baza Basin, southeast Spain): filling the gap of the early Pliocene succession in the Betics. *Journal of Vertebrate Paleontology* 37:e1338294.

Pradel A. 1981. Biometrical Remarks on the Hamster *Cricetulus migratorius* (Pallas 1773) (Rodentia, Mammalia) from Krak des Chevaliers (Syria). *Acta Zool. Cracov.* 25:271–292.

Pradel A. 1985. Morphology of the hamster *Cricetus cricetus* (Linnaeus, 1758) from Poland with some remarks on the evolution of this species. *Acta Zool. Cracov.* 29:29–52.

Pradel A. 1988. Fossil hamsters (Cricetinae, Rodentia) from the Pliocene and Quaternary of Poland. *Acta Zool Crakov* 13:235–296.

Prieto J, Angelone C, Casanovas-Vilar I, Gross M, Hír J, van den Hoek Ostende LW, Maul LC, Vasilyan D. 2014. The small mammals from Gratkorn: an overview. *Palaeobiodiversity and Palaeoenvironments* 94:135–162.

Prieto J, Rummel M. 2009a. The genus *Collimys* Daxner-Höck, 1972 (Rodentia, Cricetidae) in the Middle Miocene fissure fillings of the Frankian Alb (Germany). *Zitteliana* A 48/49:75–88.

Prieto J, Rummel M. 2009b. Evolution of the genus *Collimys* Daxner-Höck, 1972 (Rodentia, Cricetidae) – a key to Middle to Late Miocene biostratigraphy in Central Europe. *Neues Jahrbuch für Geologie und Paläontologie - Abhandlungen* 252:237–247.

Prieto J, Rummel M. 2016. Some considerations on small mammal evolution in Southern Germany, with emphasis on Late Burdigalian–Earliest Tortonian (Miocene) cricetid rodents. *Comptes Rendus Palevol* 15:837–854.

Qiu Z. 1995. A New Cricetid From the Lufeng Hominoid Locality, Late Miocene of China. *Vertebrata Palasiatica* 33:61–74.

Qiu Z, Li Q. 2016. Neogene rodents from central Nei Mongol. *Palaeontologia Sinica, New Series C* 30:1–684.

Qiu Z, Wang X, Li Q. 2013. Chapter 5. Neogene faunal succession and biochronology of central Nei Mongol (Inner Mongolia). In: Wang X, Flynn LJ, Fortelius M eds. *Fossil Mammals of Asia*. New York: Columbia University Press, 155–186.

Rook L. 2016. Geopalaeontological setting, chronology and palaeoenvironmental evolution of the Baccinello-Cinigiano Basin continental successions (Late Miocene, Italy). *Comptes Rendus Palevol* 15:825–836.

Rook L, Harrison T, Engesser B. 1996. The taxonomic status and biochronological implications of new finds of *Oreopithecus* from Baccinello (Tuscany, Italy). *Journal of human evolution* 30:3–27.

Rosina VV, Sinitsa MV. 2014. Bats (Chiroptera, Mammalia) from the Turolian of the Ukraine: phylogenetic and biostratigraphic considerations. *Neues Jahrbuch für Geologie und Paläontologie - Abhandlungen* 272:147–166.

Ruiz Bustos A, Sesé C, Dabrio C, Peña JA, Padial J. 1984. Geología y fauna de micromamíferos del nuevo yacimiento del Plioceno inferior de Gorafe-A (depresión de Guadix-Baza, Granada). *Estudios Geológicos* 40:231–242.

Ruiz-Sánchez FJ, Freudenthal M, Mansino S, Crespo VD, Montoya P. 2014. *Apocricetus barrierei* (Rodentia, Mammalia) from La Bullana 2B and La Bullana 3 (Cabriel Basin, Valencia, Spain). Revision of the Late Miocene–Early Pliocene forms of the genus *Apocricetus*. *Paläontologische Zeitschrift* 88:85–98.

Sabol M, Joniak P, Bilgin M, Bonilla-Solomón I, Cailleaux F, Čerňanský A, Malíková V, Šedivá M, Tóth C. 2021. Updated Miocene mammal biochronology of Slovakia. Geologica Carpathica: the journal of Geological Institute of Slovak Academy of Sciences 72:425–443.

Sabol M, Joniak P, Holec P. 2004. Succession(-s) of mammalian assemblages during the Neogene - a case study from the Slovak part of the Western Carpathians. *Geology* 31:65–84.

Savorelli A. 2013. New data on the Cricetidae from the Miocene “Terre Rosse” of Gargano (Apulia, Italy). *Geobios* 46:77–88.

Schaub S. 1930. Quartäre und jungtertiäre Hamster. *Abhandlungen der Schweizerischen paläontologischen Gesellschaft* 49:1–49.

Schaub S. 1934. Über einige fossile Simplicidentaten aus China und der Mongolei. *Abhandlungen der Schweizerischen paläontologischen Gesellschaft* 54:1–40.

Schaub S. 1944. Cricetodontiden der spanischen Halbinsel. *Eclogae Geologicae Helvetiae* 37:453–457.

Şen Ş. 1991. Stratigraphie, faunes de mammifères et magnétostratigraphie du Néogène de Sinap Tepe, Province d’Ankara, Turquie. *Bulletin du Muséum National d’Histoire naturelle* 12:243–277.

Sinitsa MV. 2010. Cricetids (Мammalia, Rodentia) from the Upper Miocene of Egorovka locality. *Vestnik zoologii* 44:209–225.

Sinitsa MV. 2012. Cricetids (Mammalia, Rodentia) from the Late Miocene Locality Palievo, Southern Ukraine. *Vestnik zoologii* 46:137–147.

Sinitsa MV, Delinschi A. 2016. The earliest member of *Neocricetodon* (Rodentia: Cricetidae): a redescription of *N. moldavicus* from Eastern Europe, and its bearing on the evolution of the genus. *Journal of Paleontology* 90:771–784.

Sinitsa MV, Nesin VA. 2018. Systematics and phylogeny of *Vasseuromys* (Mammalia, Rodentia, Gliridae) with a description of a new species from the late Miocene of eastern Europe. *Palaeontology* 61:679–701.

Tedford RH, Qiu Z-X, Ye J. 2013. Cenozoic Geology of the Yushe Basin. In: Tedford RH, Qiu Z-X, Flynn LJ eds. *Late Cenozoic Yushe Basin, Shanxi Province, China: Geology and Fossil Mammals: Volume I: History, Geology, and Magnetostratigraphy*. Vertebrate Paleobiology and Paleoanthropology . Dordrecht: Springer Netherlands, 35–67.

Topachevsky VA, Skorik AF. 1992. *Neogene and Pleistocene primitive Cricetidae of the south of Eastern Europe*. Kiev: Naukova Dumka Press, Institute of Zoology Kiev.

Torres-Roig E, Agustí J, Bover P, Alcover JA. 2019. A new giant cricetine from the basal Pliocene of Mallorca (Balearic Islands, western Mediterranean): biostratigraphic nexus with continental mammal zones. *Historical biology* 31:559–573.

Vangengeim EA, Lungu AN, Tesakov AS. 2006. Age of the Vallesian lower boundary (Continental Miocene of Europe). *Stratigraphy and geological correlation = Stratigrafiya, geologicheskaya korrelyatsiya* 14:655–667.

Vasileiadou K, Sylvestrou I. 2022. The Fossil Record of Rodents (Mammalia: Rodentia) in Greece. In: Vlachos E ed. *Fossil Vertebrates of Greece Vol. 1: Basal vertebrates, Amphibians, Reptiles, Afrotherians, Glires, and Primates*. Cham: Springer International Publishing, 407–610.

Vasiliev I, Iosifidi AG, Khramov AN, Krijgsman W, Kuiper K, Langereis CG, Popov VV, Stoica M, Tomsha VA, Yudin SV. 2011. Magnetostratigraphy and radio-isotope dating of upper Miocene–lower Pliocene sedimentary successions of the Black Sea Basin (Taman Peninsula, Russia). *Palaeogeography, palaeoclimatology, palaeoecology* 310:163–175.

van de Weerd AA. 1976. Rodent faunas of the Mio-Pliocene continental sediments of the Teruel-Alfambra region, Spain. *Utrecht Micropaleontological Bulletin spec. publ.* 2:1–217.

Wu W. 1991. The Neogene mammalian faunas of Ertemte and Harr Obo in Inner Mongolia (Nei Mongol), China. 9. Hamsters: Cricetinae (Rodentia). *Senckenbergiana Lethaea* 71:257–305.

Wu W, Flynn LJ. 2017. The Hamsters of Yushe Basin. In: Flynn LJ, Wu W eds. *Late Cenozoic Yushe Basin, Shanxi Province, China: Geology and Fossil Mammals: Volume II: Small Mammal Fossils of Yushe Basin*. Vertebrate Paleobiology and Paleoanthropology . Dordrecht: Springer Netherlands, 123–137.

Xie K, Zhang Y, Li Y. 2021. Revision to *Kowalskia* from the Houhecun Fauna and a New Discovery of *Tscherskia* (Cricetidae, Rodentia) from the Youhe Fauna of Weinan, Shaanxi Province, China. *Acta Geologica Sinica - English Edition* 95:1073–1079.

Xie K, Zhang Y, Li Y. 2023. Large-sized fossil hamsters from the late Middle Pleistocene Locality 2 of Shanyangzhai, China, and discussion on the validity of *Cricetinus* and *C. varians* (Rodentia: Cricetidae). *PeerJ* 11:e15604.

Xijun N, Zhuding Q. 2002. The micromammalian fauna from the Leilao, Yuanmou hominoid locality: implications for biochronology and paleoecology. *Journal of human evolution* 42:535–546.

Young C-C. 1927. Fossile Nagetiere aus Nord-China. *Palaeontologia Sinica, Series C* 5:1–82.

Zheng S-H. 1984. A new species of *Kowalskia* (Rodentia, Mammalia) of Yinan. *Vertebrata PalAsiatica* 22:251–260.
