# Supplementary material for: Phylogenetic relationships of Neogene hamsters (Mammalia, Rodentia, Cricetinae) revealed under Bayesian inference and maximum parsimony
Source: PeerJ. 2024 Nov 15;12:e18440. doi: 10.7717/peerj.18440 (PMC11572387; doi:10.7717/peerj.18440)
Supplement: Supplemental Information 5 [file peerj-12-18440-s005.docx]

**Supplemental file S5 for**

Phylogenetic relationships of Neogene hamsters (Mammalia, Rodentia, Cricetinae) revealed under Bayesian inference and maximum parsimony

Moritz Dirnberger^1^, Pablo Peláez-Campomanes^2^, Raquel López-Antoñanzas^1^

^1^ISEM, Univ Montpellier, CNRS, IRD, Montpellier, France

^2^Departamento de Paleobiología, Museo Nacional de Ciencias Naturales-CSIC, Madrid, Spain

**Additional trees based on the pruned and the complete taxon set**

Table of contents

Additional trees with pruned taxon set 2

Bayesian inference non-clock tree 2

Bayesian inference Tk02 tree 2

Maximum parsimony equal weighting tree 2

Additional trees with complete taxon set 2

*Neocricetodon ambarrensis* 3

*Apocricetus darderi* 3

*Pseudocricetus polgardiensis* 3

Figure S5.1. MCC tree of the non-clock Bayesian inference analysis 4

Figure S5.2. MCC tree of the time-calibrated relaxed-clock Tk02 Bayesian inference analysis 5

Figure S5.3. MC tree of the equal weighting maximum parsimony analysis 6

Figure S5.4. MCC tree of the non-clock Bayesian inference analysis including rogue and extant taxa 7

Figure S5.5. MCC tree of the time-calibrated relaxed-clock IGR Bayesian inference analysis including rogue and extant taxa 8

Figure S5.6. MCC tree of the time-calibrated relaxed-clock Tk02 Bayesian inference analysis including rogue and extant taxa 9

Figure S5.7. MC tree of the implied weighting maximum parsimony analysis 10

Figure S5.8. MC tree of the equal weighting maximum parsimony analysis 11

References 12

# Additional trees with pruned taxon set

## Bayesian inference non-clock tree

In the phylogeny inferred by the non-clock Bayesian inference (Fig. S5.1) is similar to the maximum parsimony analyses, as *Collimys* and *Rotundomys* are sister to each other and form a clade with *Cricetulodon* *sabadellensis* and *C. bugesiensis*. In contrast, this clade is however sister ot the *Neocricetodon* clade with the *Pseudocricetus* + *Apocricetus* + *Hattomys* + *C. meini* + *C. lucentensis* clade diverging outside.

## Bayesian inference Tk02 tree

The topology and divergence times based on the Tk02 Bayesian inference analysis (Fig. S5.2) are practically identical to the IGR tree, except for differences in weakly supported relationships within *Neocricetodon*.

## Maximum parsimony equal weighting tree

The topology and divergence times based on the equal weighting maximum parsimony analysis (Fig. S5.3) closely resembles the implied weighting tree. Differences can only be seen in the position of *Neocricetodon browni* and *N. nestori*, that are positioned within *Neocricetodon*, while *N. fahlbuschi* and *N. moldavicus* are resolved outside the genus.

# Additional trees with complete taxon set

The two extant taxa are resolved in all analyses (Figs. S5.4–8) within the clade of *Apocricetus* + *Hattomys* + *Cricetulodon* *meini* and *C*. *lucentensis*, with slight differences in the exact positions. In both clock trees, they form a clade (PP ≥ 0.71) as sister to *Apocricetus s.s.*, while they are split up in the non-clock analyses.

Including the rogue taxa leads to noticeably decreased posterior probabilities (e.g., for *Neocricetodon* in the IGR clock tree, Fig. S5.5: PP = 0.14 vs. 0.52). The topologies of the trees only changed in the case of the IGR clock-tree, with differences within *Neocricetodon* and regarding *Cricetulodon hartenbergeri*, *Cricetulodon sabadellensis* and *Cricetulodon bugesiensis* that form a clade with *Pseudocricetus* (PP = 0.12). The instability of the positions of the rogue taxa becomes also clear in the comparison of the different analyses. *Neocricetodon* *ambarrensis* is recovered at a basal position in the *Neocricetodon* clade in all analyses except for the implied weighting maximum parsimony, where it is not positioned within *Neocricetodon* but more basally. *Apocricetus* *darderi* shows two possible relationships: either as sister to *Hattomys* (Fig. S5.6, Tk02: PP = 0.74; Fig. S5.7, MP-IW: BS < 50; Fig. S5.8, MP-EW: BS < 50) or within *Apocricetus s.s.* as sister to *A. angustidens* + *A. alberti* (Fig. S5.4, Bayesian non-clock: PP = 0.43; Fig. S5.5, IGR: PP = 0.49). Similarly, *Pseudocricetus* *polgardiensis* is either positioned within *Neocricetodon*, as sister to *N. progressus* (Fig. S5.4, Bayesian non-clock: PP = 0.29; Fig. S5.6, Tk02: PP = 0.36; Fig. S5.8, MP-EW: BS < 50) or within *Pseudocricetus* (Fig. S5.5, IGR: PP = 0.36; Fig. S5.8, MP-IW: BS < 50). The posterior probabilities are rather low for all placements, underlining the ‘rogue’ character of the taxa.

## *Neocricetodon ambarrensis*

The basal position of *Neocricetodon ambarrensis* within *Neocricetodon*, as reconstructed by all analyses, can be clearly seen in its medium sized mesoloph(id)s and labial spur of the anterolophule in the M1, as proposed earlier (Freudenthal, Mein & Martín Suárez, 1998; Sinitsa & Delinschi, 2016). Congruent to *N. moldavicus*, the third synapomorphy, the anterior metalophule in the M2, is clearly present, supporting the assignment to *Neocricetodon*.

## *Apocricetus darderi*

The designation of *Apocricetus darderi* as a rogue taxon is a result of its similarities to *Apocricetus s.s.*, e.g., the crest-like anteroconid in the m1 and reduced protolophule in the M2, but also to *Hattomys*, e.g., the gigantism and the structure of combined mesolophid and hypolophulid in the lower molars, as reflected in its varying position in the different trees. Considering the geographical distribution of *Hattomys* in Gargano, Italy and *A. darderi* in Mallorca, Spain it is plausible, to attribute the similarities to a convergent insular evolution from a common ancestor (Torres-Roig et al., 2019).

## *Pseudocricetus polgardiensis*

Freudenthal et al. (1998) mention the association of the former ‘*Cricetus* *polgardiensis*’ Freudenthal and Kordos (1989) to *Pseudocricetus* but without elaboration (see also Qiu & Li, 2016). Other authors favour a relationship to *Neocricetodon* and treat it as such (Daxner-Höck, 1992; Daxner-Höck et al., 1996; Sinitsa & Delinschi, 2016). *Pseudocricetus polgardiensis* shows several similarities to *Neocricetodon*, including the m1 mesolophid and M1 labial spur of the anterolophulid that both reach the molar border and the presence of the metalophule in the M2 (Topachevsky & Skorik, 1992). In the other species of *Pseudocricetus*, including the presumably most basal species *P. antiquus*, the mesolophids in the m1 are strongly reduced and the mesolophs form an anterior metalophule, while the labial spur of the anterolophule forms a labial anterolophulid (Topachevsky & Skorik, 1992). The association of *P*. *polgardiensis* with the other two *Pseudocricetus* species as seen in the IGR clock tree and the implied weighting maximum parsimony tree (Figs. S5.5, S5.8), is probably a consequence of the coding of an absence of the labial anterolophid in the m1, which is at least poorly developed in all *Neocricetodon* species. This character is however difficult to assess, based on the available drawings of the species (Freudenthal & Kordos, 1989). As this structure might thus also be developed in *P. polgardiensis*, a transfer to *Neocricetodon*, as seen in the other trees seems to be the most reasonable.

**
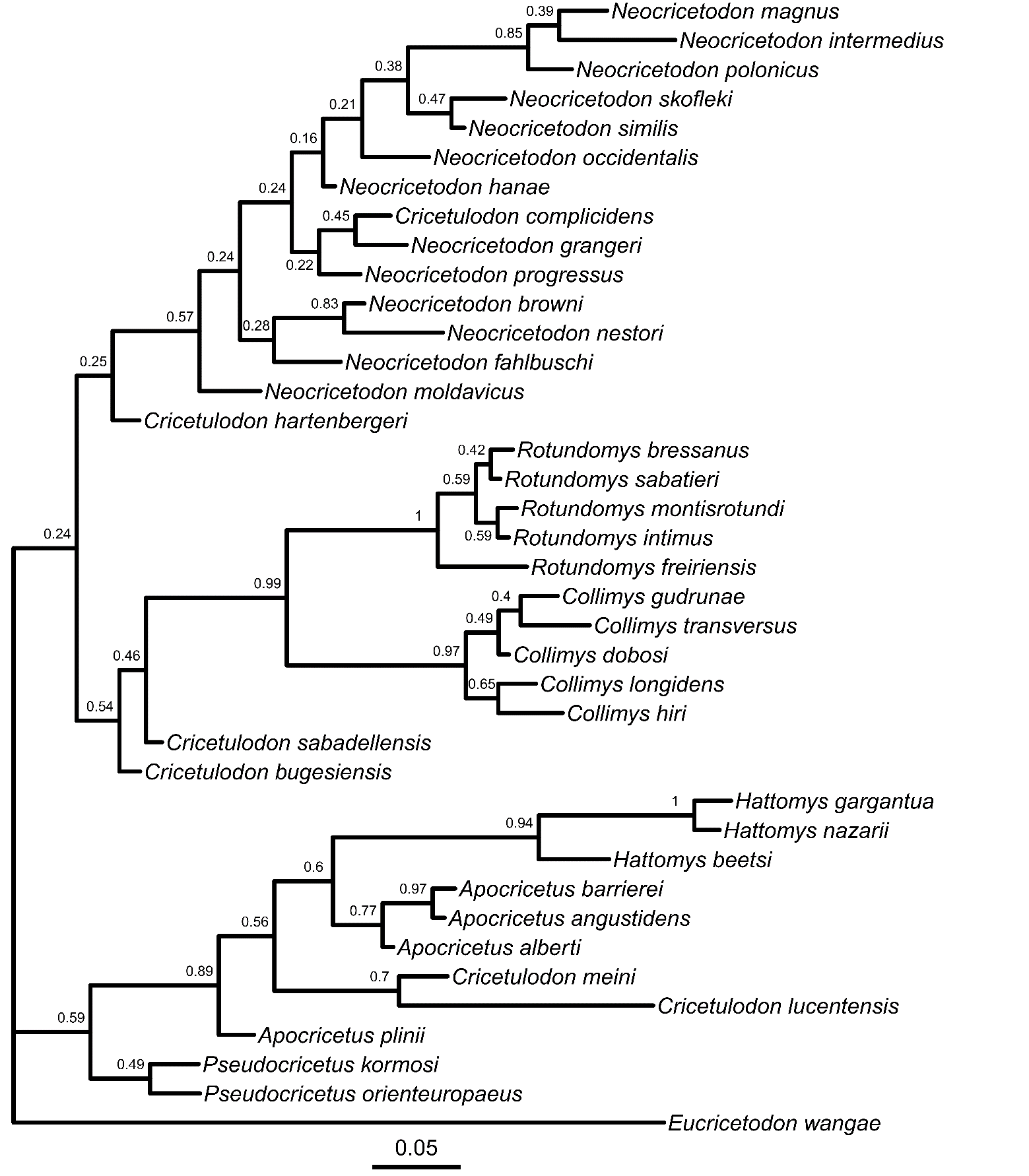
**

Figure S5.1. MCC tree of the non-clock Bayesian inference analysis**.** Numbers at nodes depict the posterior probability. Scale bar represents character state changes per character.


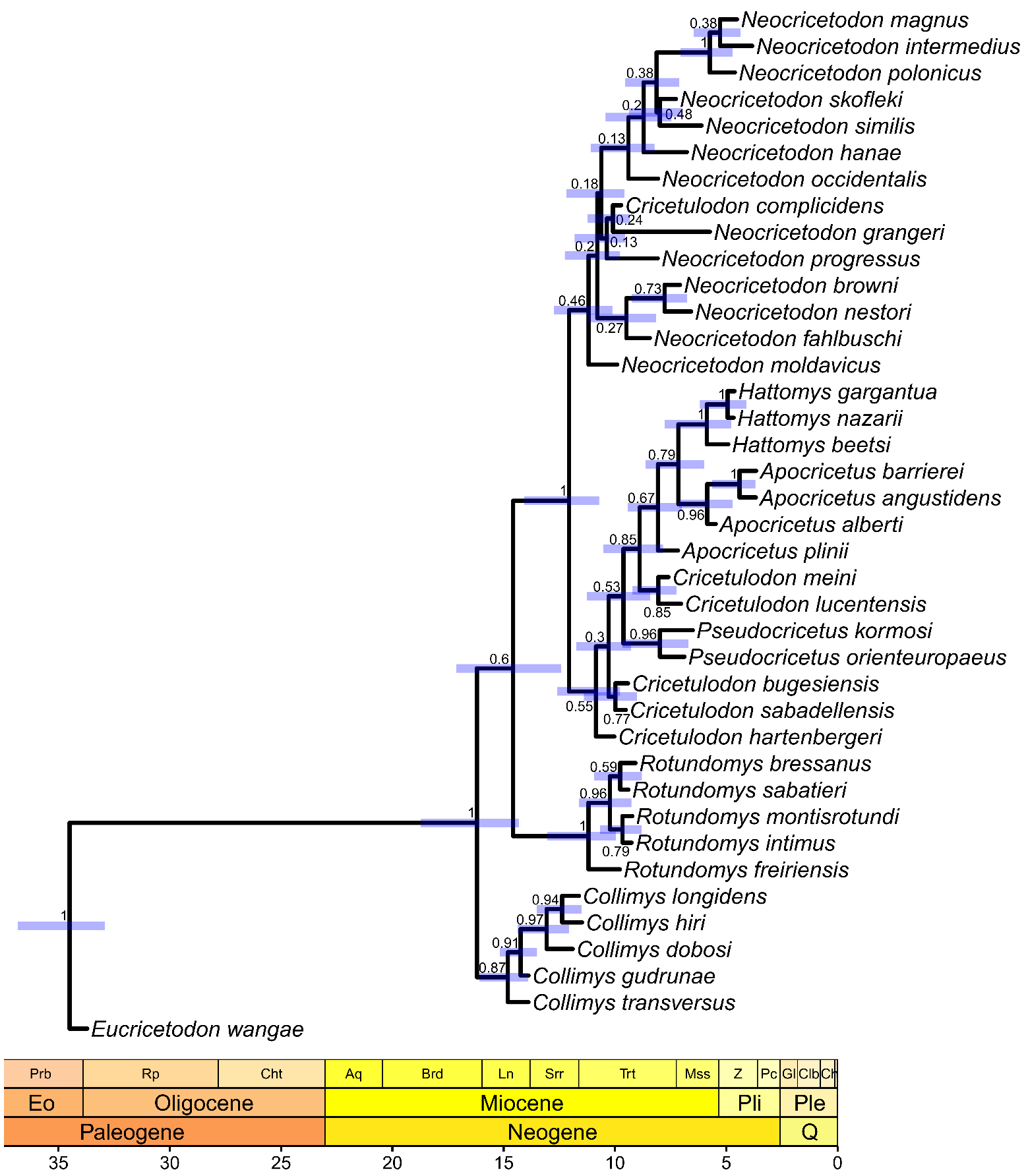


Figure S5.2. MCC tree of the time-calibrated relaxed-clock Tk02 Bayesian inference analysis**.** Numbers at nodes depict the posterior probability, node bars indicate the 95% highest posterior density for divergence times. Scale axis in Ma, chronostratigraphic chart following Cohen, Harper and Gibbard (2022).


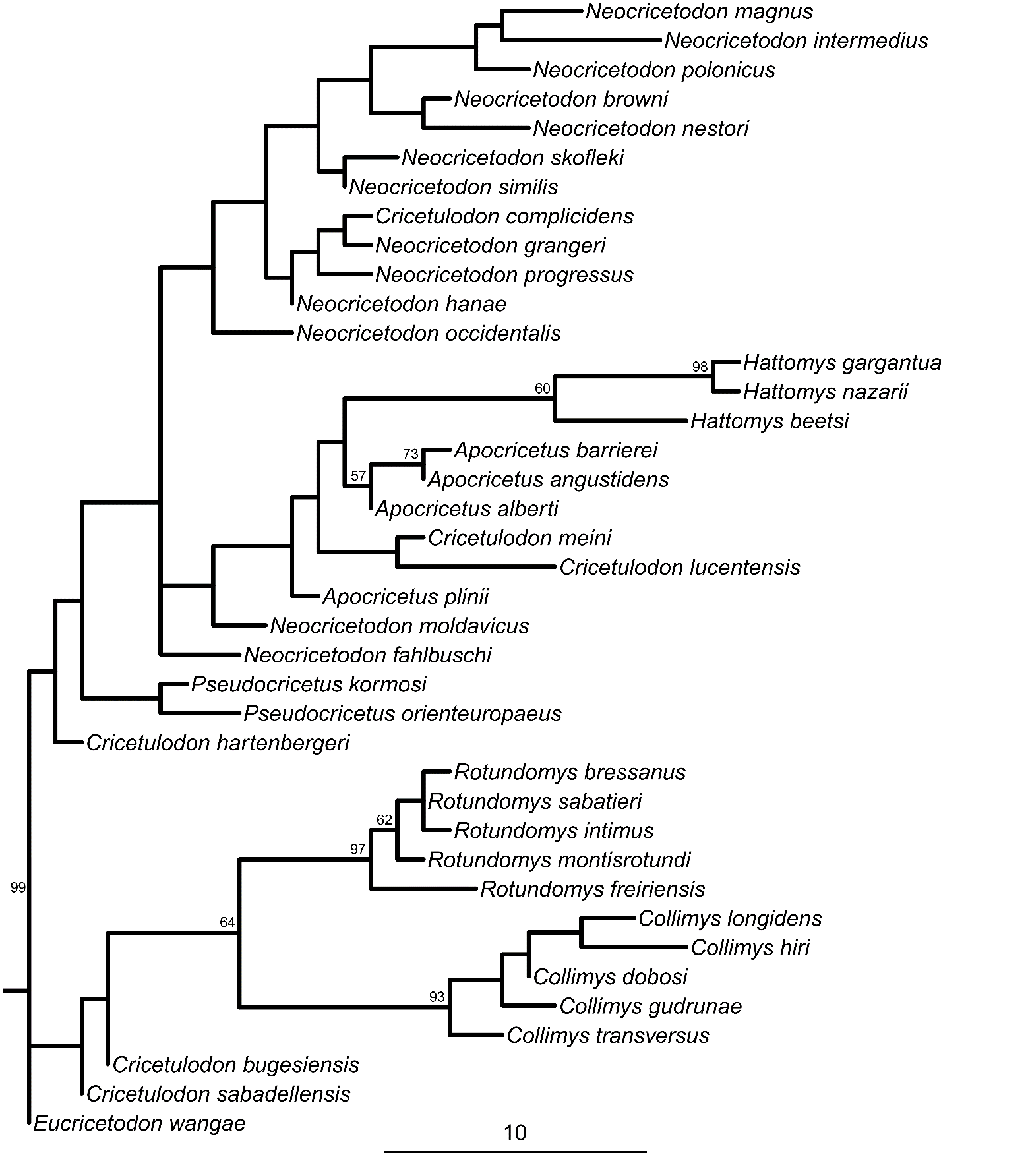


Figure S5.3. MC tree of the equal weighting maximum parsimony analysis. Calculated from 106 most parsimonious trees. Bootstrap values over 50% indicated at respective nodes. Scale bar represents character state changes.


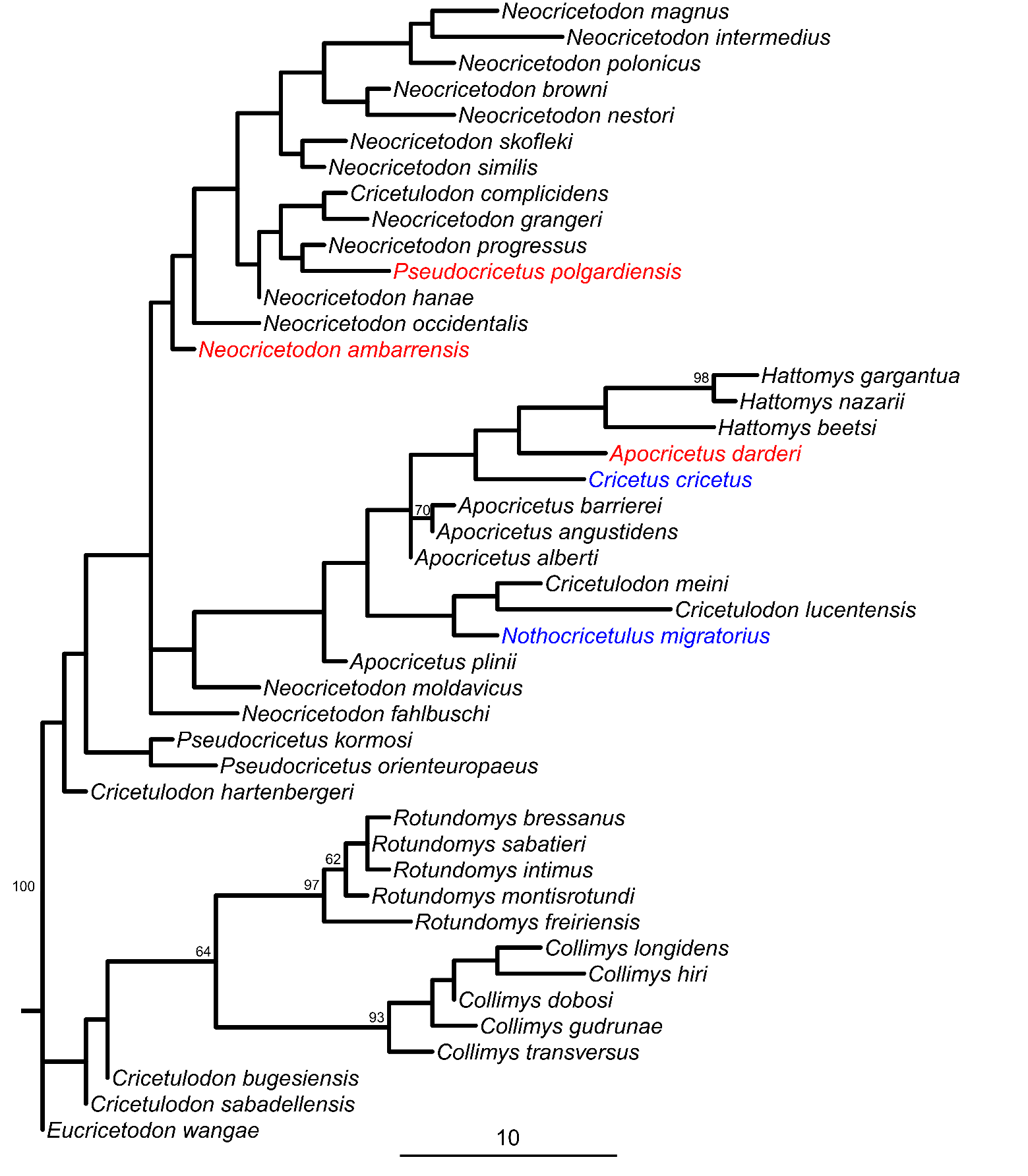


Figure S5.4. MCC tree of the non-clock Bayesian inference analysis including rogue and extant taxa. Extant taxa in blue, rogue taxa in red. Numbers at nodes depict the posterior probability. Scale bar represents character state changes per character.


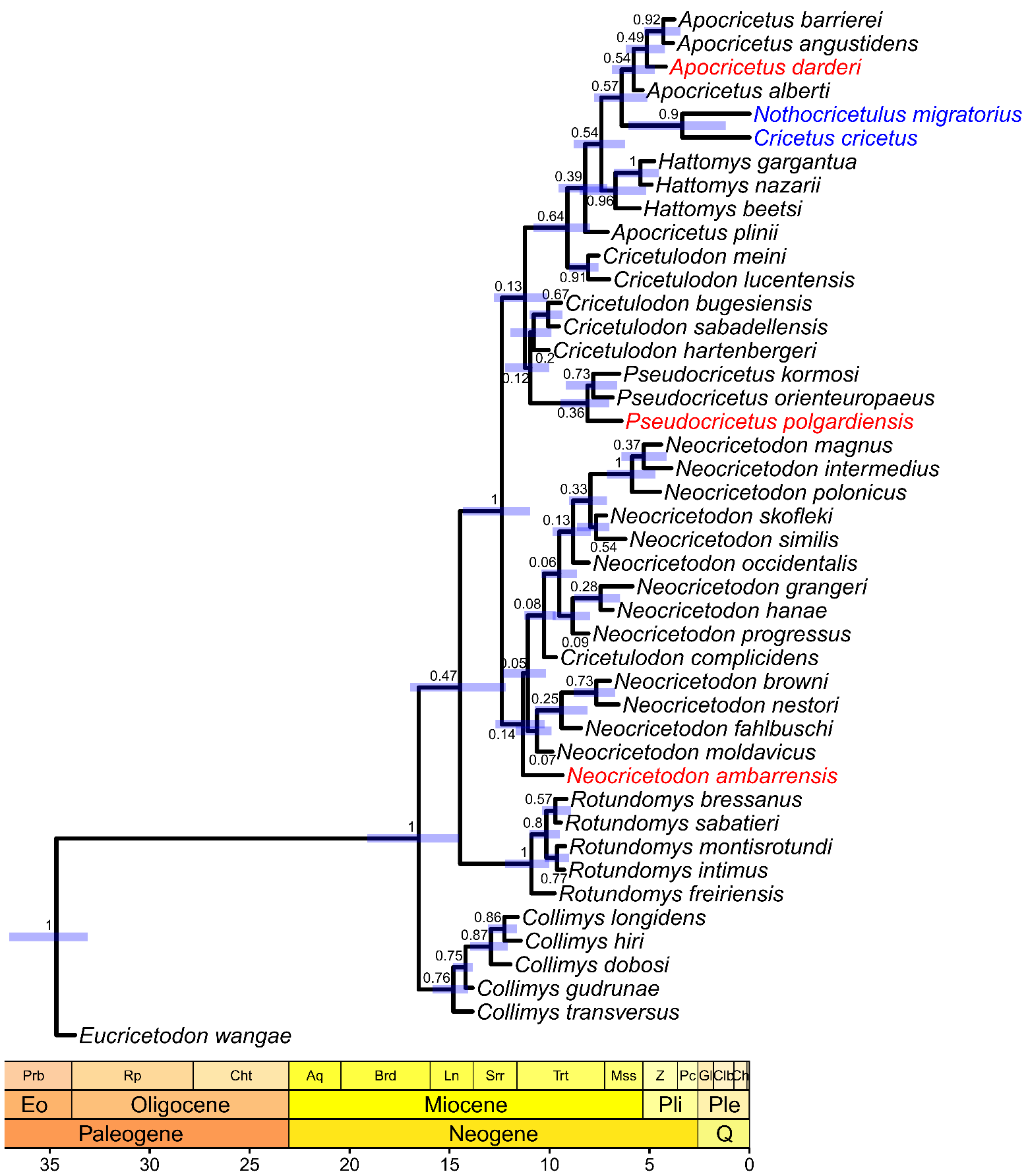


Figure S5.5. MCC tree of the time-calibrated relaxed-clock IGR Bayesian inference analysis including rogue and extant taxa. Extant taxa in blue, rogue taxa in red. Numbers at nodes depict the posterior probability, node bars indicate the 95% highest posterior density for divergence times. Scale axis in Ma, chronostratigraphic chart following Cohen, Harper and Gibbard (2022).


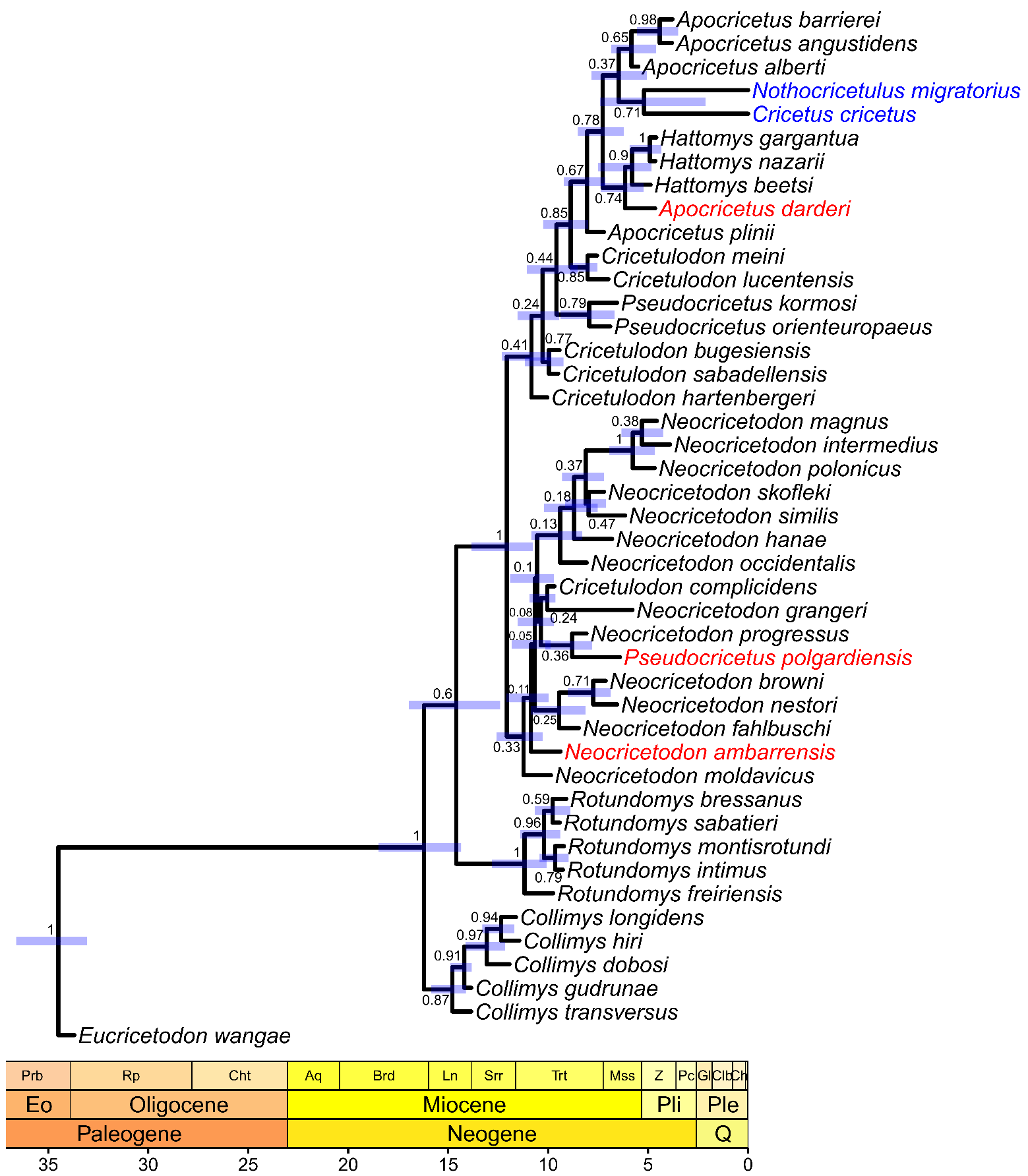


Figure S5.6. MCC tree of the time-calibrated relaxed-clock Tk02 Bayesian inference analysis including rogue and extant taxa. Extant taxa in blue, rogue taxa in red. Numbers at nodes depict the posterior probability, node bars indicate the 95% highest posterior density for divergence times. Scale axis in Ma, chronostratigraphic chart following Cohen, Harper and Gibbard (2022).


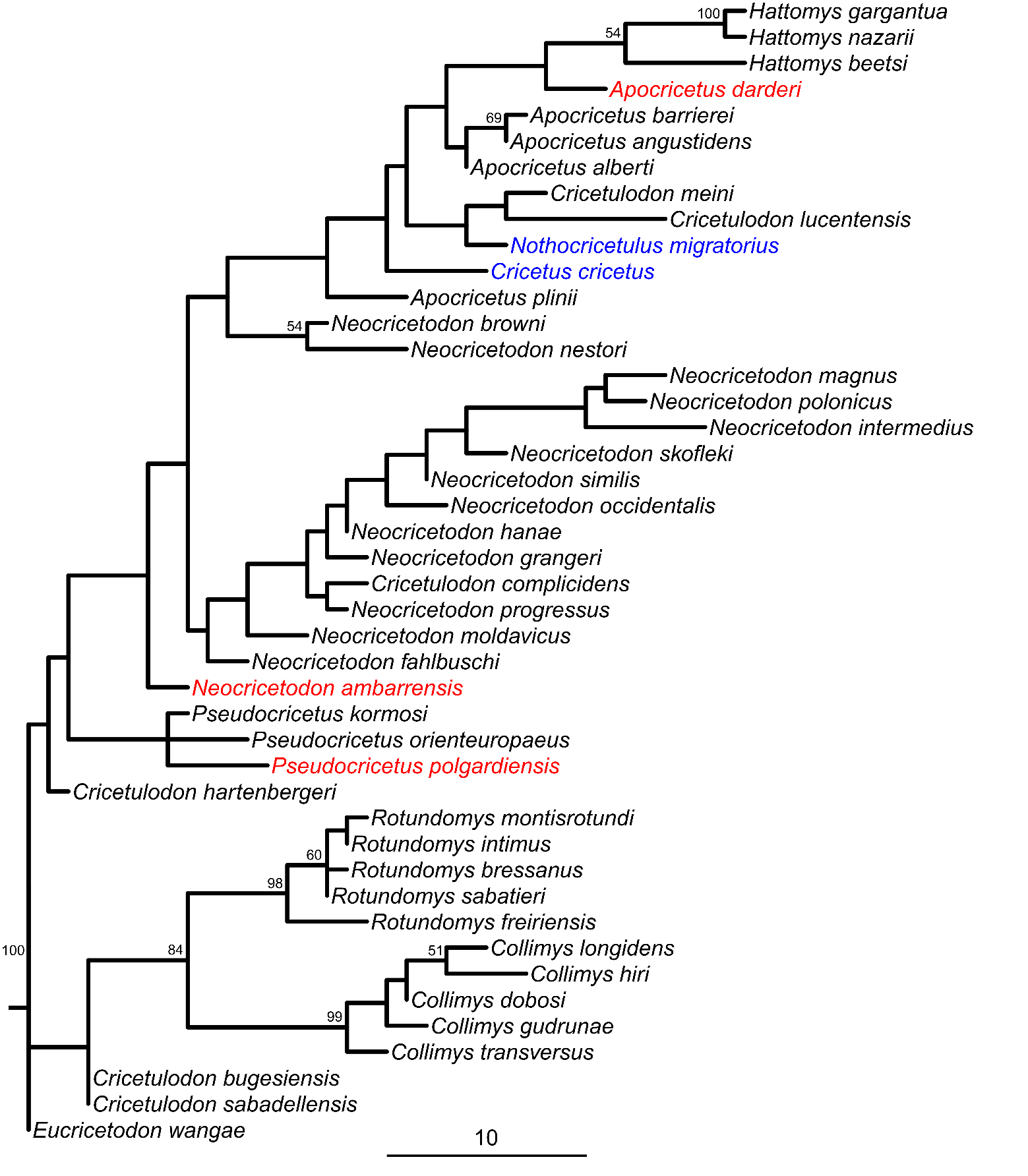


Figure S5.7. MC tree of the implied weighting maximum parsimony analysis including rogue and extant taxa. Calculated from 2 most parsimonious trees. Extant taxa in blue, rogue taxa in red. Bootstrap values over 50% indicated at respective nodes. Scale bar represents character state changes.


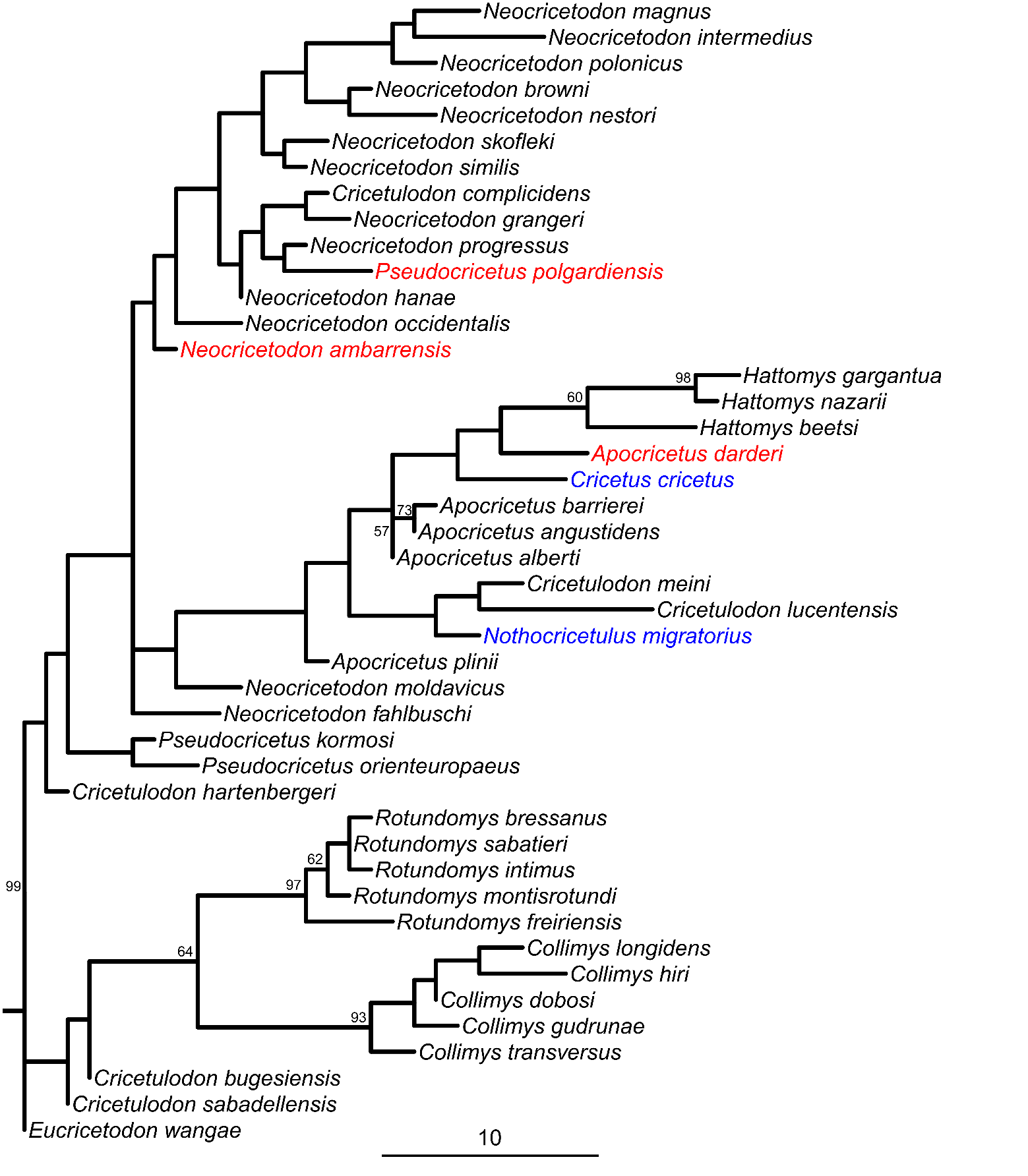


Figure S5.8. MC tree of the equal weighting maximum parsimony analysis including rogue and extant taxa. Calculated from 106 most parsimonious trees. Extant taxa in blue, rogue taxa in red. Bootstrap values over 50% indicated at respective nodes. Scale bar represents character state changes.

# References

Cohen KM, Harper DAT, Gibbard PL. 2022.ICS International Chronostratigraphic Chart 2022/10. *Available at* *www.stratigraphy.org* (accessed January 31, 2023).

Daxner-Höck G. 1992. Die Cricetinae aus dem Obermiozän von Maramena (Mazedonien, Nordgriechenland). *Paläontologische Zeitschrift* 66:331–367.

Daxner-Höck G, Fahlbusch V, Kordos L, Wu W. 1996. The Late Neogene cricetid genera *Neocricetodon* and *Kowalskia*. In: Bernor RL, Fahlbusch V, Mittmann HW eds. *The Evolution of Western Eurasian Neogene Mammal Faunas*. New York City: Columbia University Press, 220–226.

Freudenthal M, Kordos L. 1989. *Cricetus polgardiensis* sp. nov. and *Cricetus kormosi* Schaub, 1930 from the Late Miocene Polgárdi localities (Hungary). *Scripta geologica* 89:71–100.

Freudenthal M, Mein P, Martín Suárez E. 1998. Revision of Late Miocene and Pliocene Cricetinae (Rodentia, Mammalia) from Spain and France. *Treballs del Museu de Geologia de Barcelona* 7:11–93.

Qiu Z, Li Q. 2016. Neogene rodents from central Nei Mongol. *Palaeontologia Sinica, New Series C* 30:1–684.

Sinitsa MV, Delinschi A. 2016. The earliest member of *Neocricetodon* (Rodentia: Cricetidae): a redescription of *N. moldavicus* from Eastern Europe, and its bearing on the evolution of the genus. *Journal of Paleontology* 90:771–784.

Topachevsky VA, Skorik AF. 1992. *Neogene and Pleistocene primitive Cricetidae of the south of Eastern Europe*. Kiev: Naukova Dumka Press, Institute of Zoology Kiev.

Torres-Roig E, Agustí J, Bover P, Alcover JA. 2019. A new giant cricetine from the basal Pliocene of Mallorca (Balearic Islands, western Mediterranean): biostratigraphic nexus with continental mammal zones. *Historical biology* 31:559–573.
