# Supplementary material for: Phylogenetic relationships of Neogene hamsters (Mammalia, Rodentia, Cricetinae) revealed under Bayesian inference and maximum parsimony
Source: PeerJ. 2024 Nov 15;12:e18440. doi: 10.7717/peerj.18440 (PMC11572387; doi:10.7717/peerj.18440)
Supplement: Supplemental Information 8 [file peerj-12-18440-s008.pdf]

Supplemental file S8 for

Phylogenetic relationships of Neogene hamsters (Mammalia, Rodentia, Cricetinae) revealed under Bayesian inference and maximum parsimony

Moritz Dirnberger[1], Pablo Peláez-Campomanes[2], Raquel López-Antoñanzas[1]

[1]ISEM, Univ Montpellier, CNRS, IRD, Montpellier, France

[2]Departamento de Paleobiología, Museo Nacional de Ciencias Naturales-CSIC, Madrid, Spain

Stochastic character mapping on the Bayesian inference IGR tree

The stochastic character mapping was performed on the time-calibrated tree under the IGR clock model with the R package phytools v. 2.1.1 (Revell, 2024). For each character the best-fit model regarding rates of character state change was determined based on the Akaike information criterion. For all of the here shown 22 characters, the equal rates model was preferred, except for characters 37 and 66. For these two cases the symmetrical rates model was chosen. For descriptions of the characters, see Supplemental File S2.

Reference

Revell LJ. 2024. phytools 2.0: an updated R ecosystem for phylogenetic comparative methods (and other things). *PeerJ* 12:e16505.

Ancestral state reconstruction of trait 1: Length of the M1

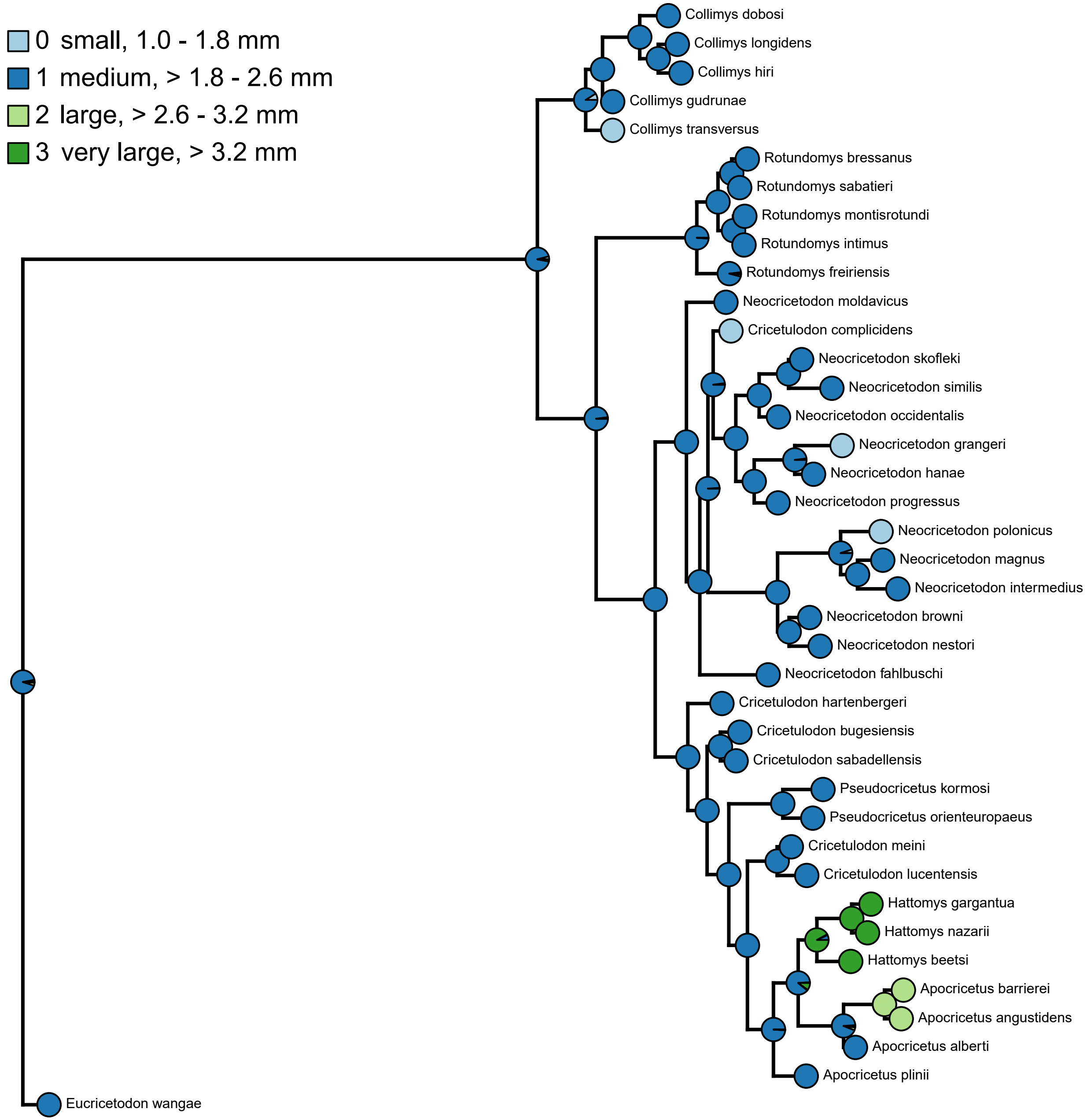

Ancestral state reconstruction of trait 5: Anterocone

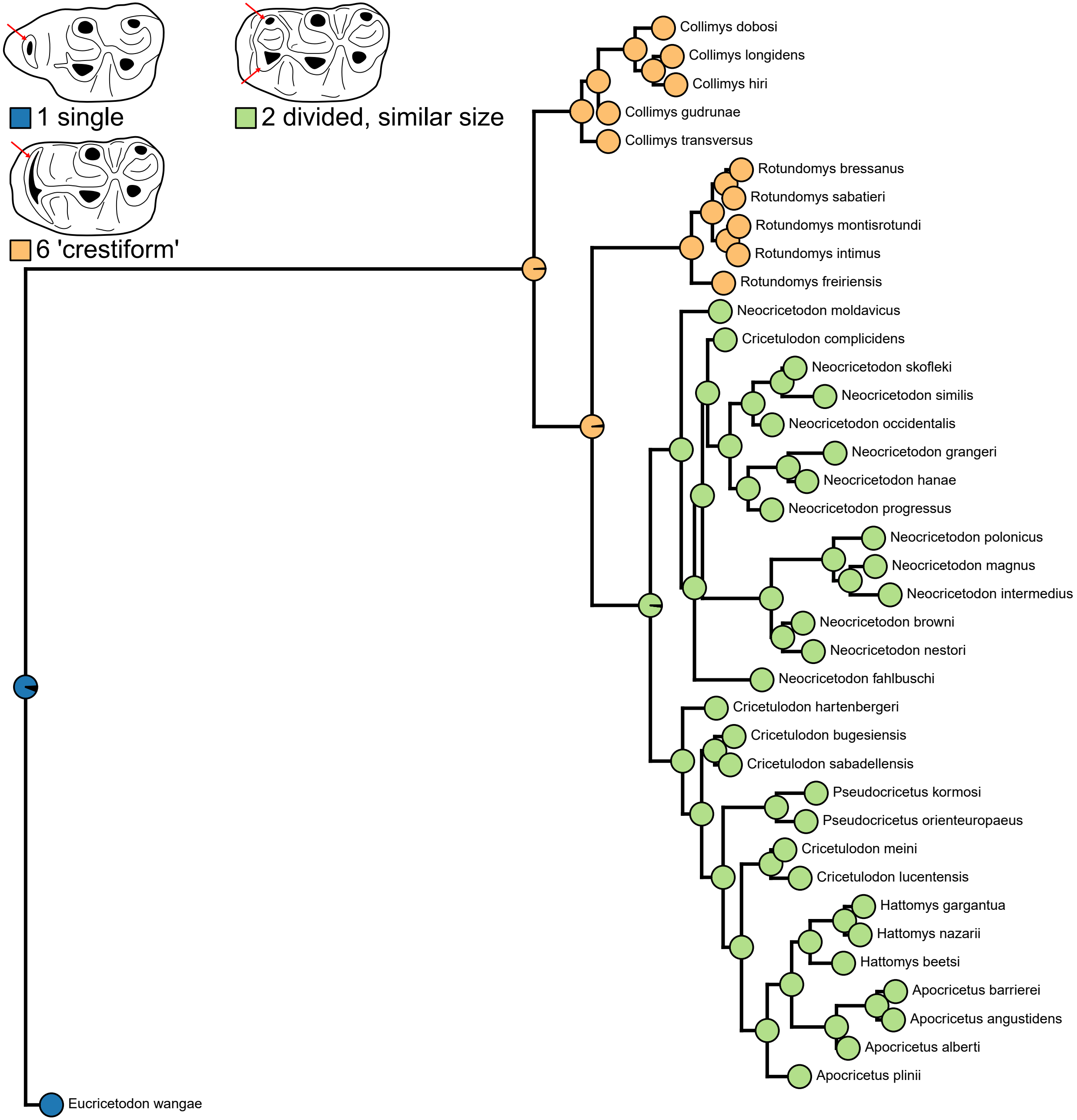

Ancestral state reconstruction of trait 7: M1 protolophule

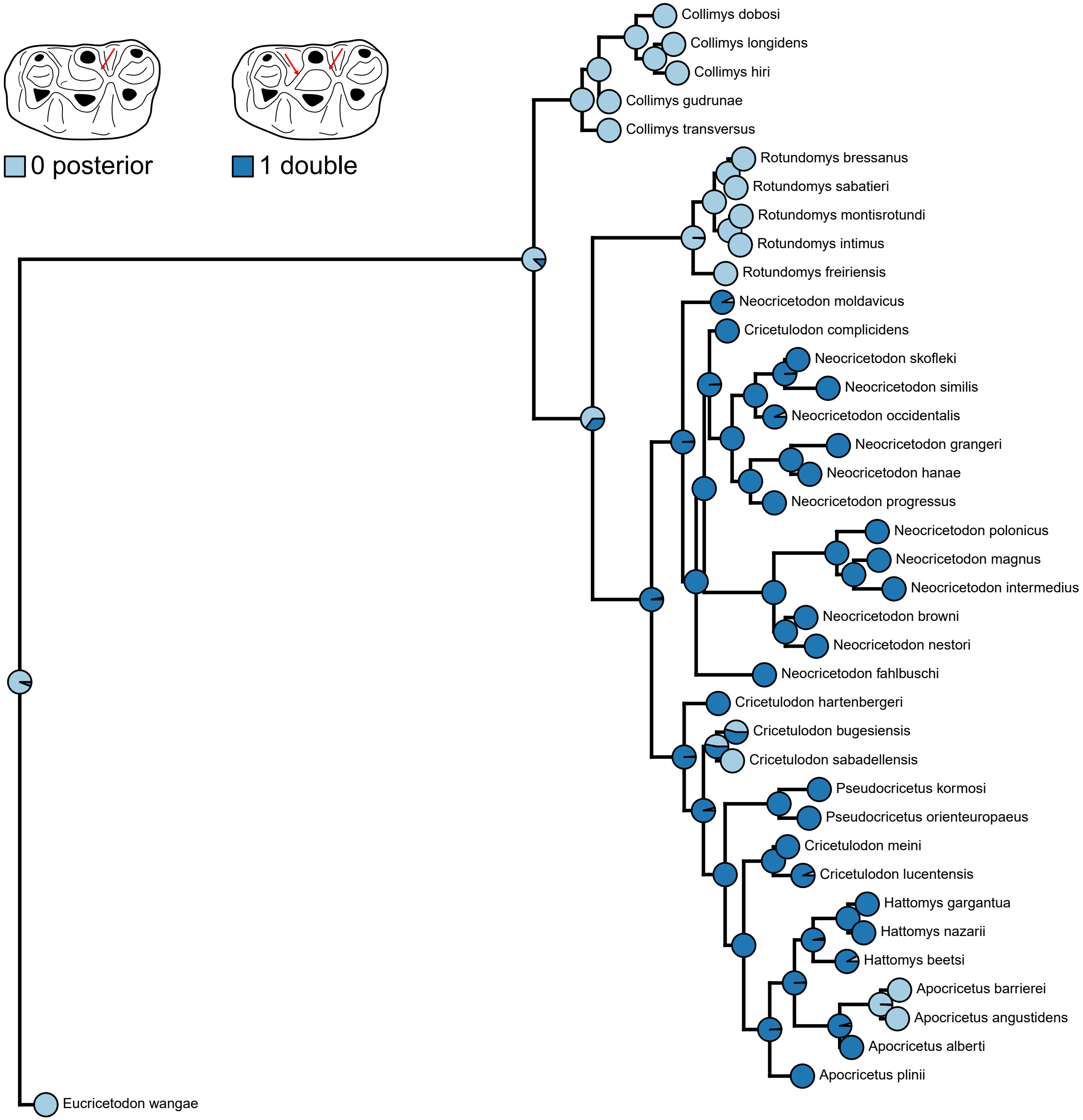

Ancestral state reconstruction of trait 11: M1 lingual anteroloph

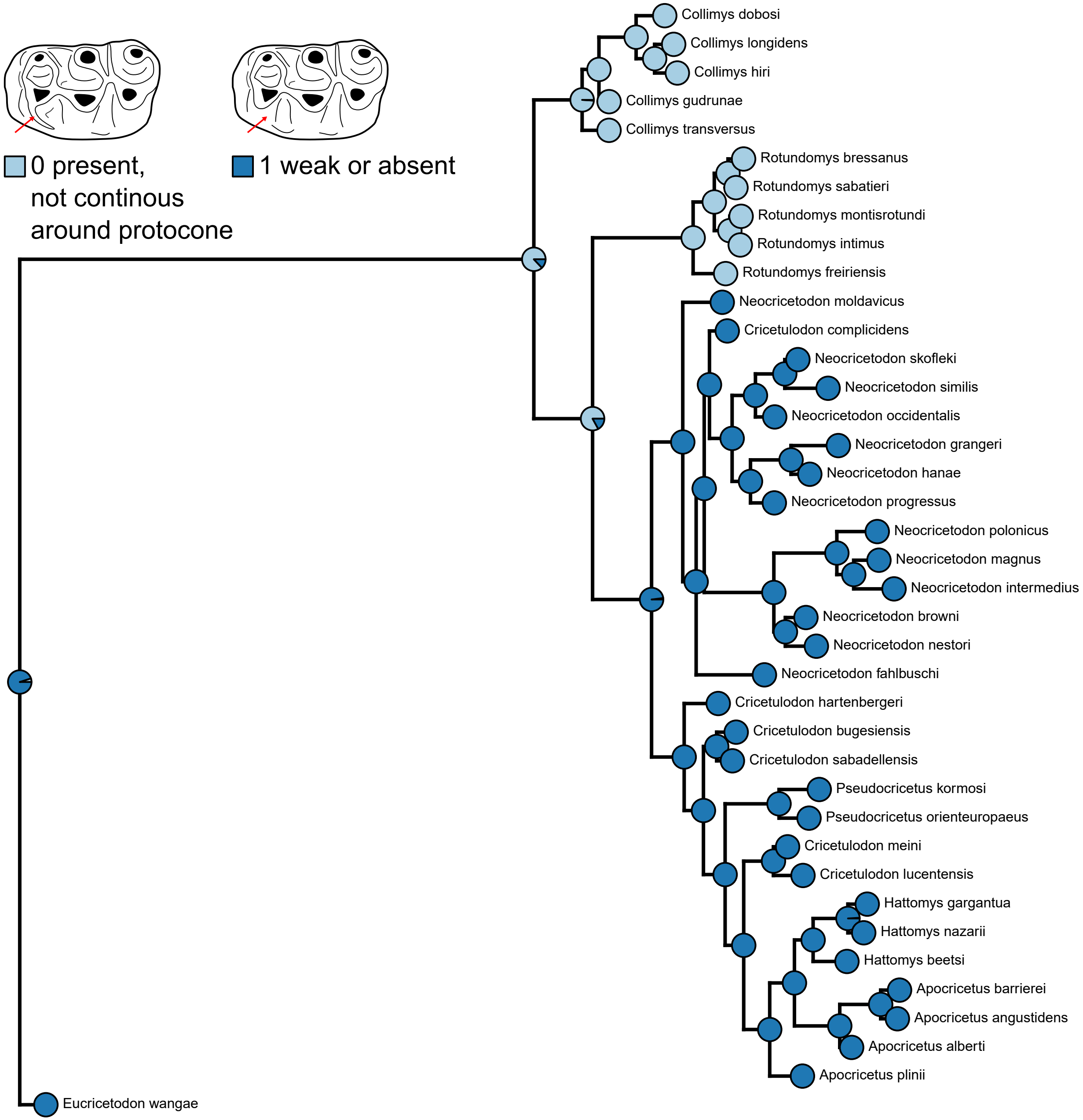

Ancestral state reconstruction of trait 20: M1 mesoloph

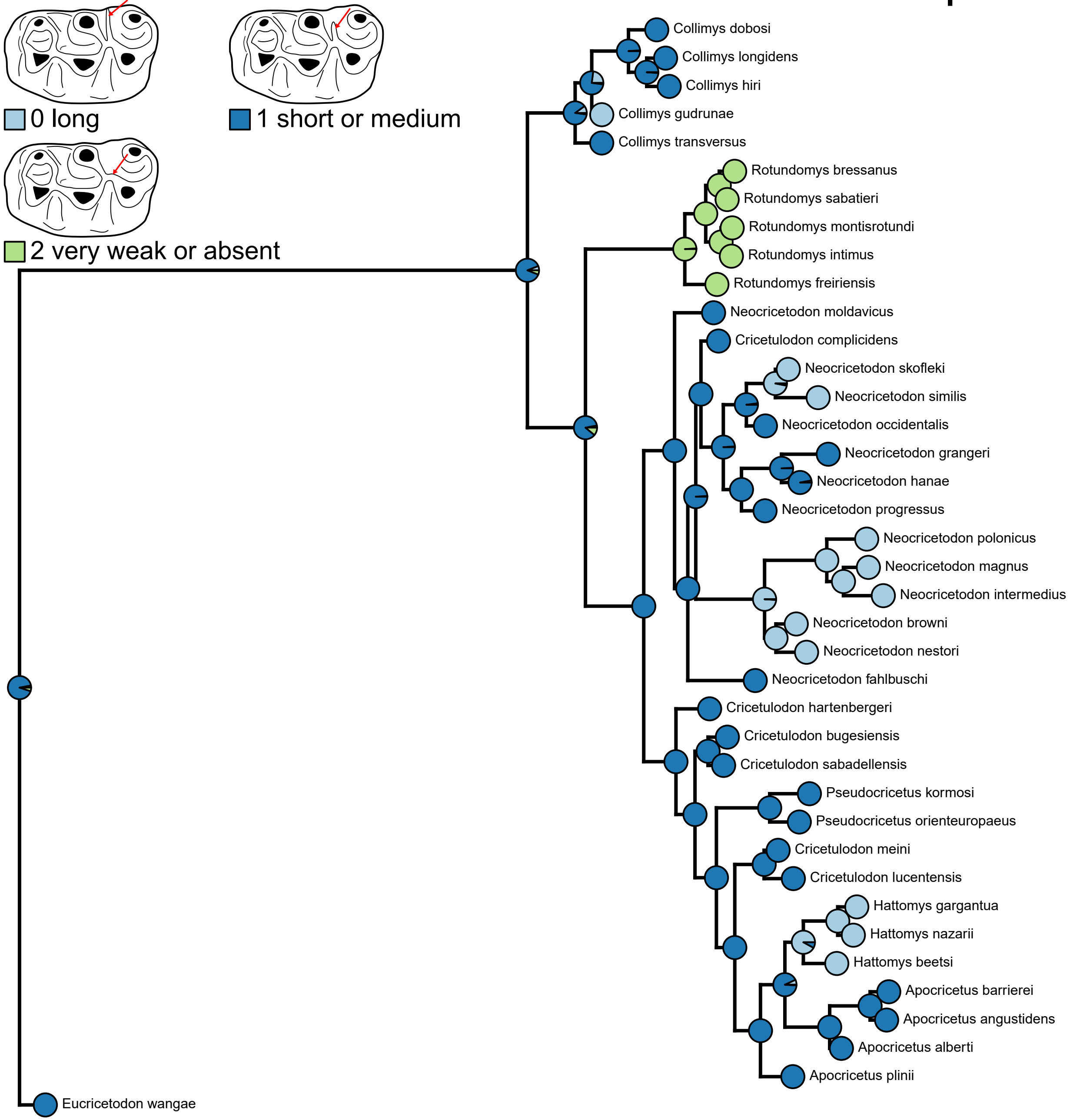

Ancestral state reconstruction of trait 26: M1 labial posteroloph

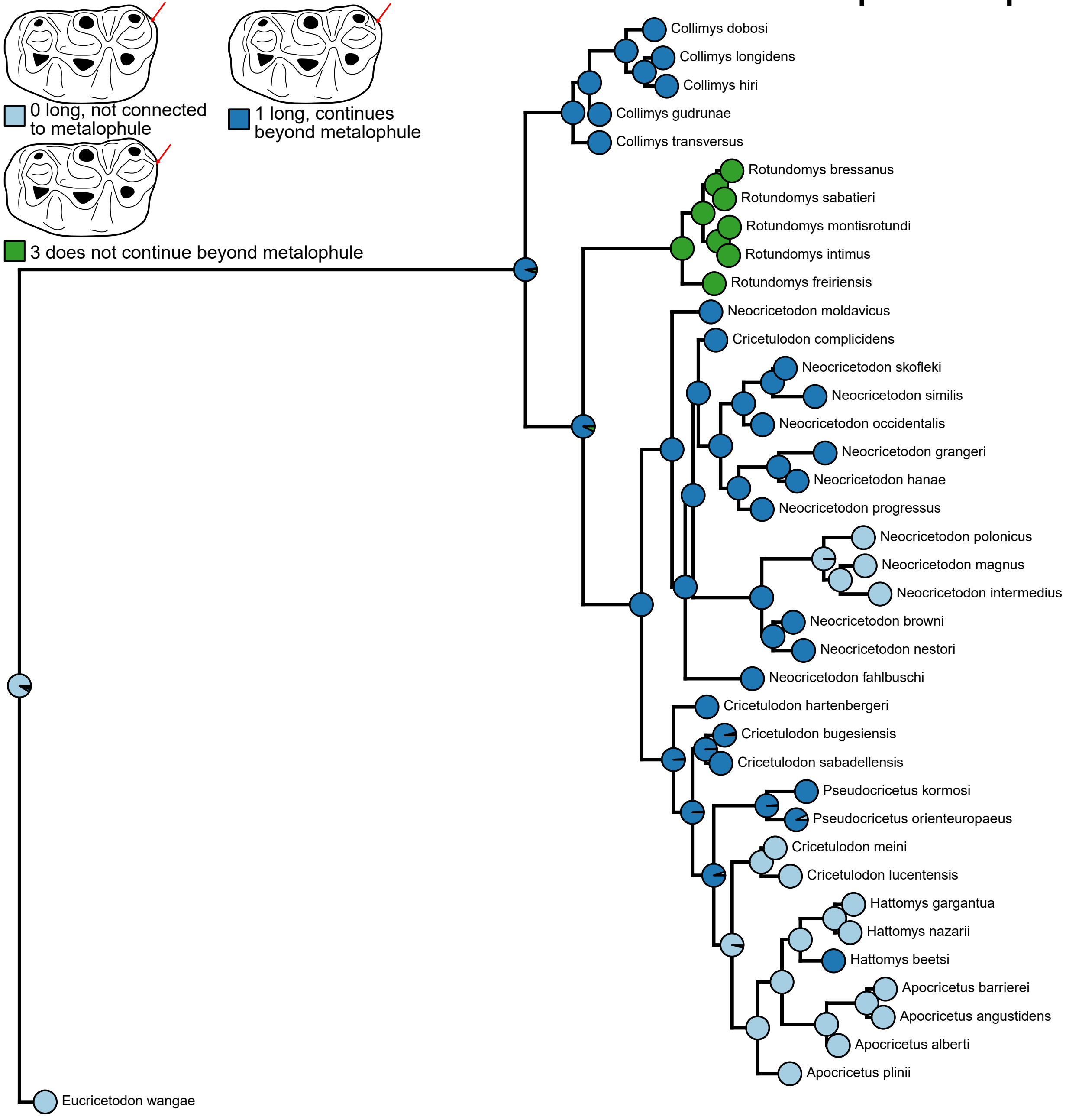

Ancestral state reconstruction of trait 34: M2 protolophule

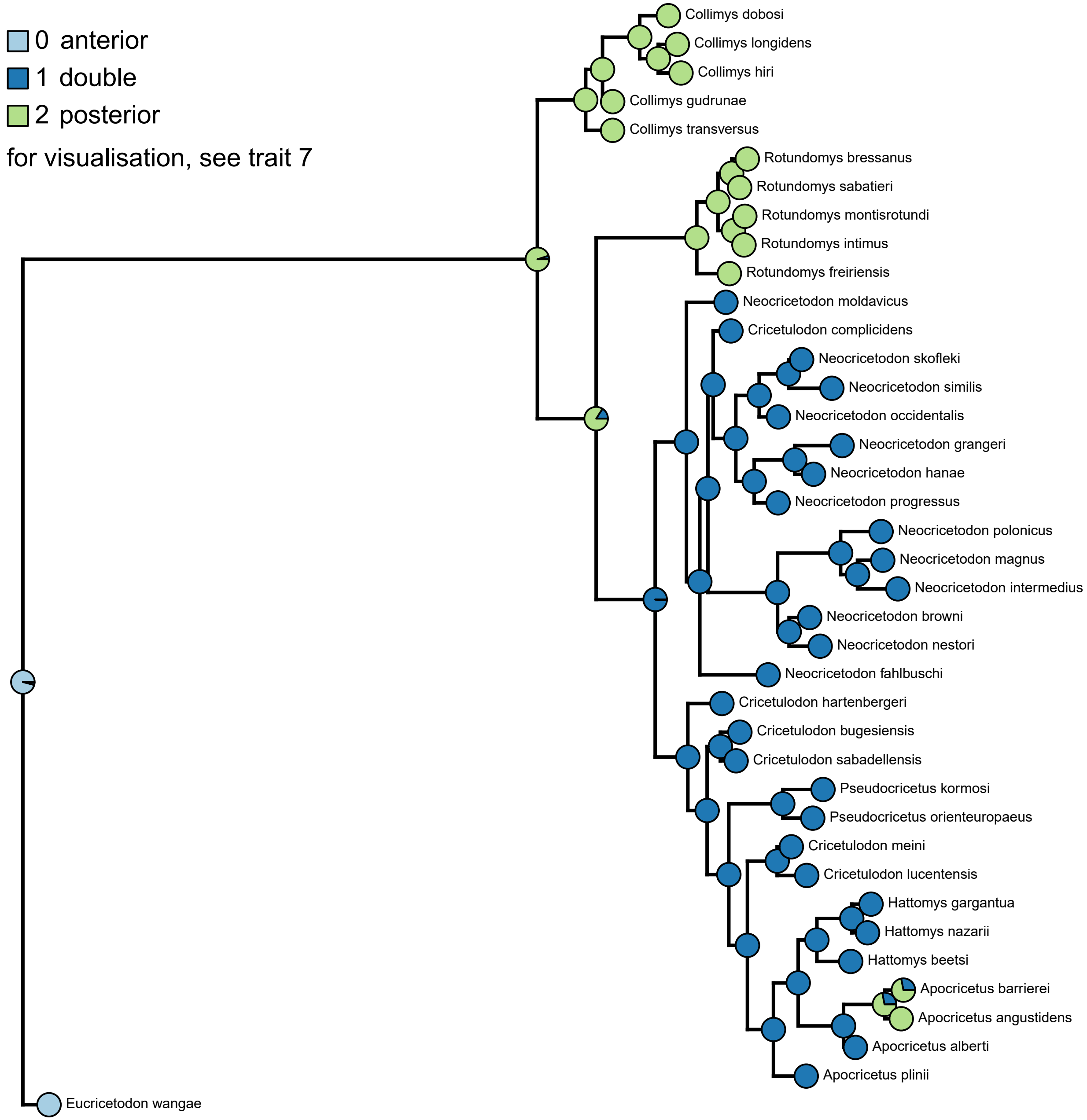

Ancestral state reconstruction of trait 37: M2 mesoloph

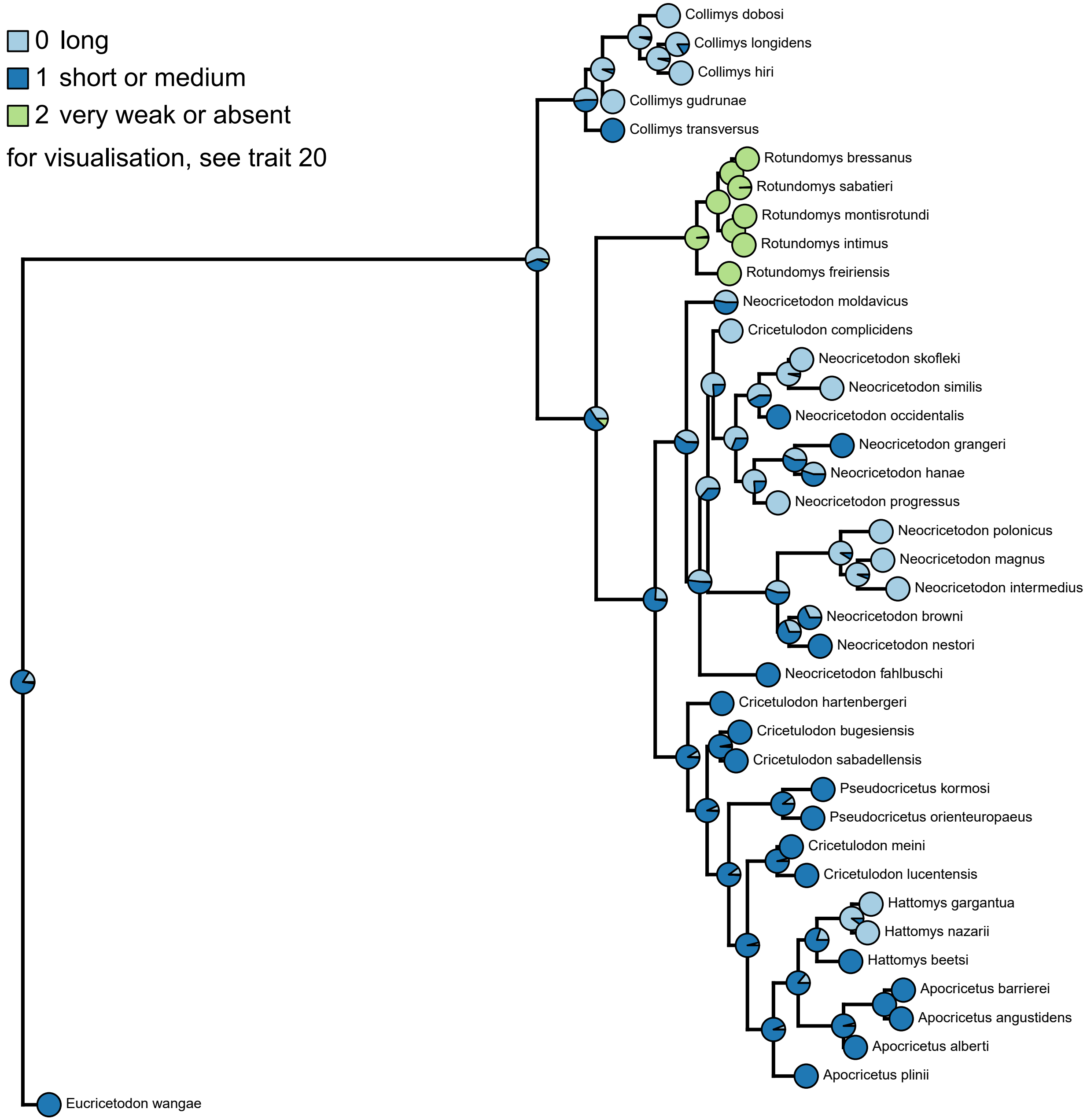

Ancestral state reconstruction of trait 38: M2 metalophule

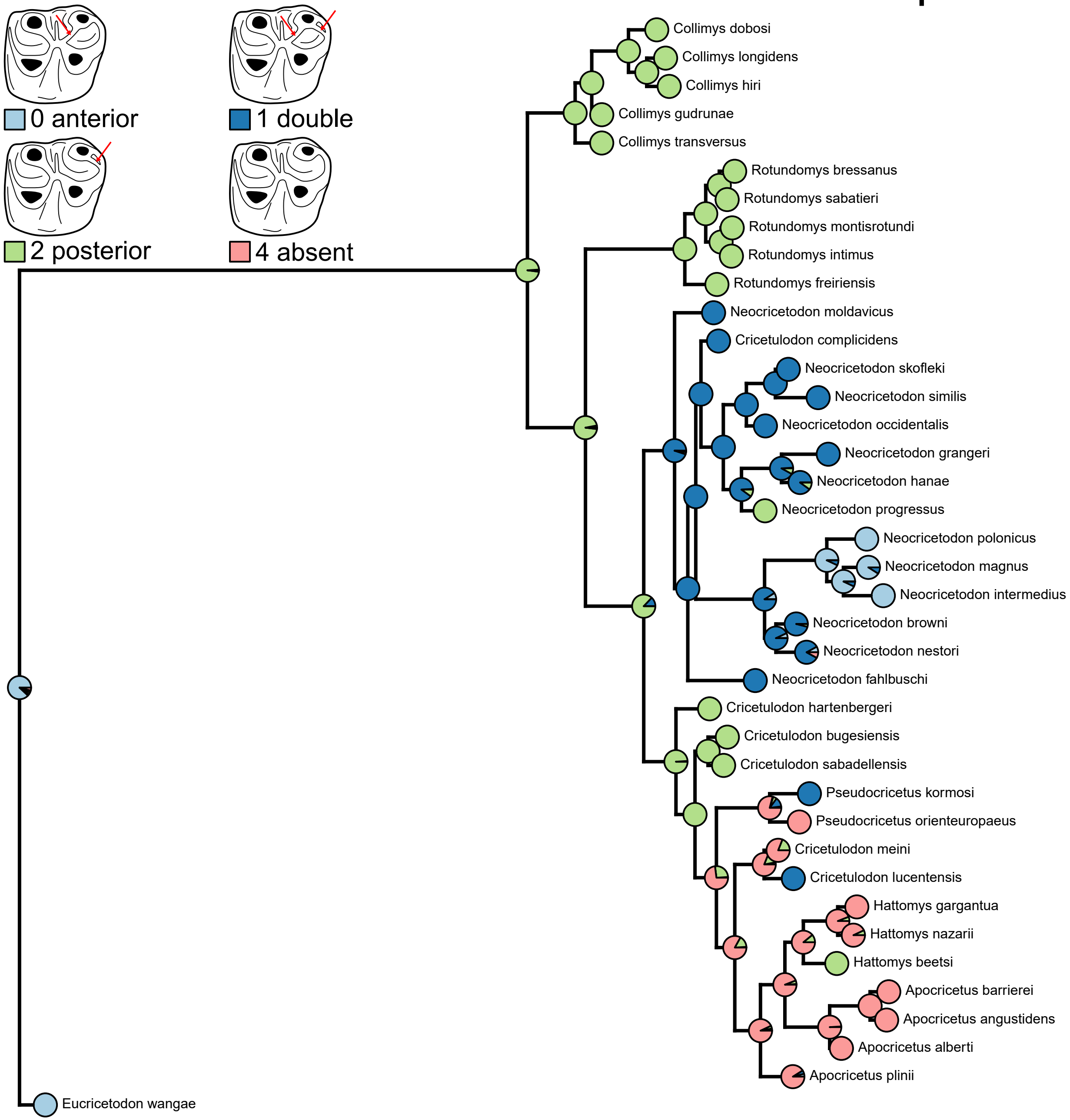

Ancestral state reconstruction of trait 47: M3 anterior protolophule

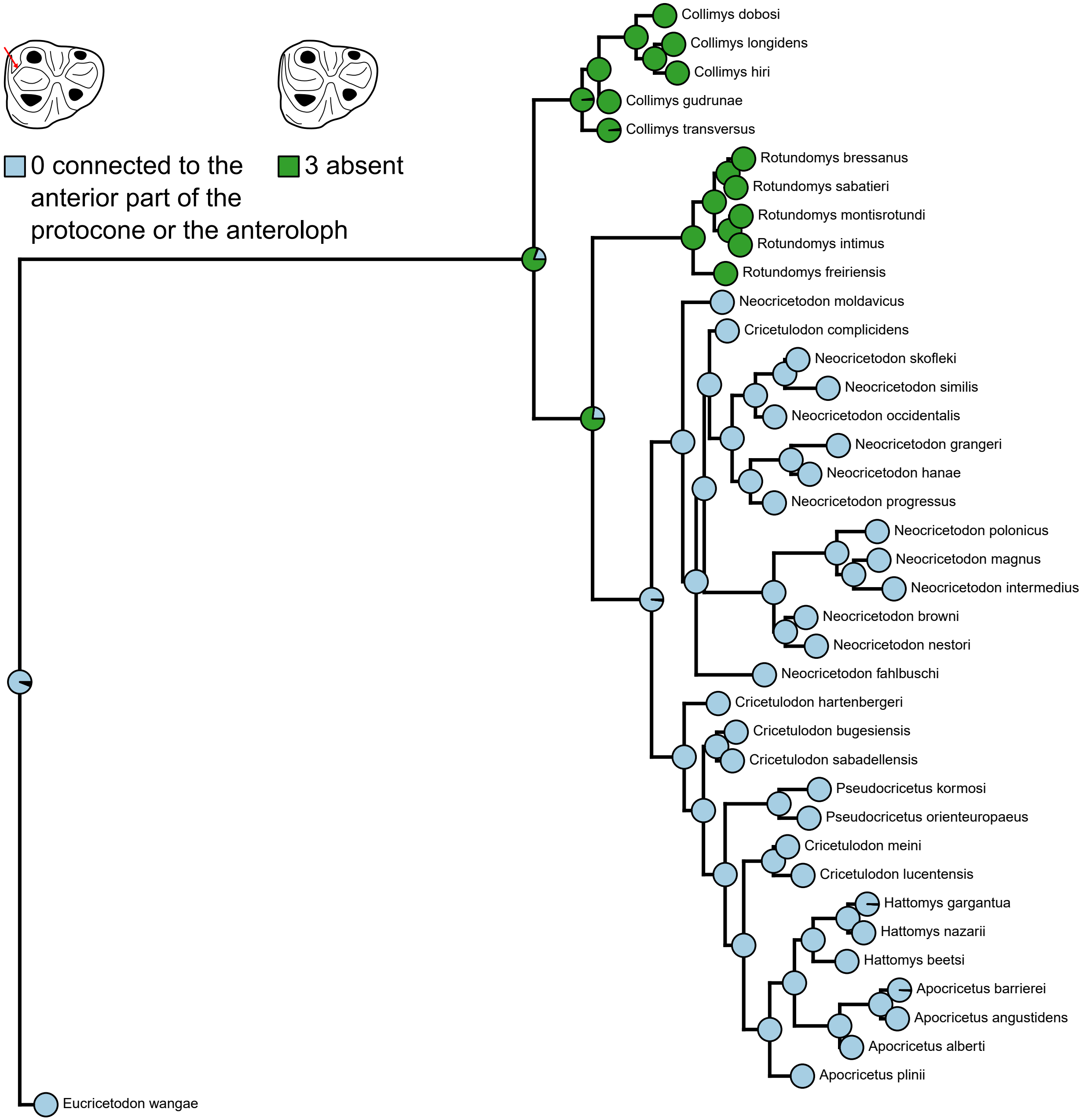

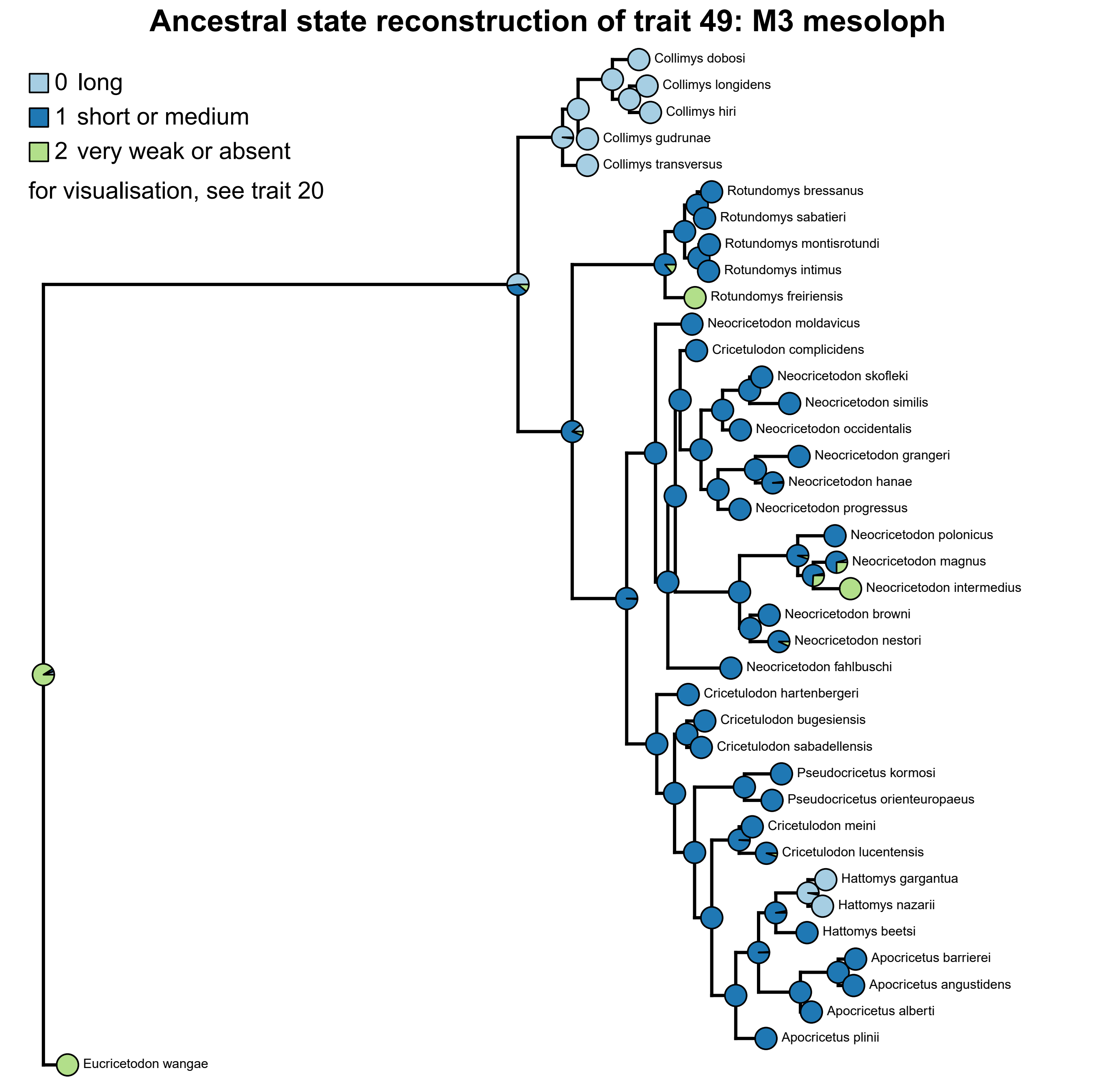
Ancestral state reconstruction of trait 49: M3 mesoloph
0 long
1 short or medium
2 very weak or absent
for visualisation, see trait 20
Collimys dobosi
Collimys longidens
Collimys hiri
Collimys gudrunae
Collimys transversus
Rotundomys bressanus
Rotundomys sabatieri
Rotundomys montisrotundi
Rotundomys intimus
Rotundomys freiriensis
Neocricetodon moldavicus
Cricetulodon complicidens
Neocricetodon skofleki
Neocricetodon similis
Neocricetodon occidentalis
Neocricetodon grangeri
Neocricetodon hanae
Neocricetodon progressus
Neocricetodon polonicus
Neocricetodon magnus
Neocricetodon intermedius
Neocricetodon browni
Neocricetodon nestori
Neocricetodon fahlbuschi
Cricetulodon hartenbergeri
Cricetulodon bugesiensis
Cricetulodon sabadellensis
Pseudocricetus kormosi
Pseudocricetus orienteuropaeus
Cricetulodon meini
Cricetulodon lucentensis
Hattomys gargantua
Hattomys nazarii
Hattomys beetsi
Apocricetus barrierei
Apocricetus angustidens
Apocricetus alberti
Apocricetus plinii
Eucricetodon wangae

Ancestral state reconstruction of trait 56: M3 posteroloph

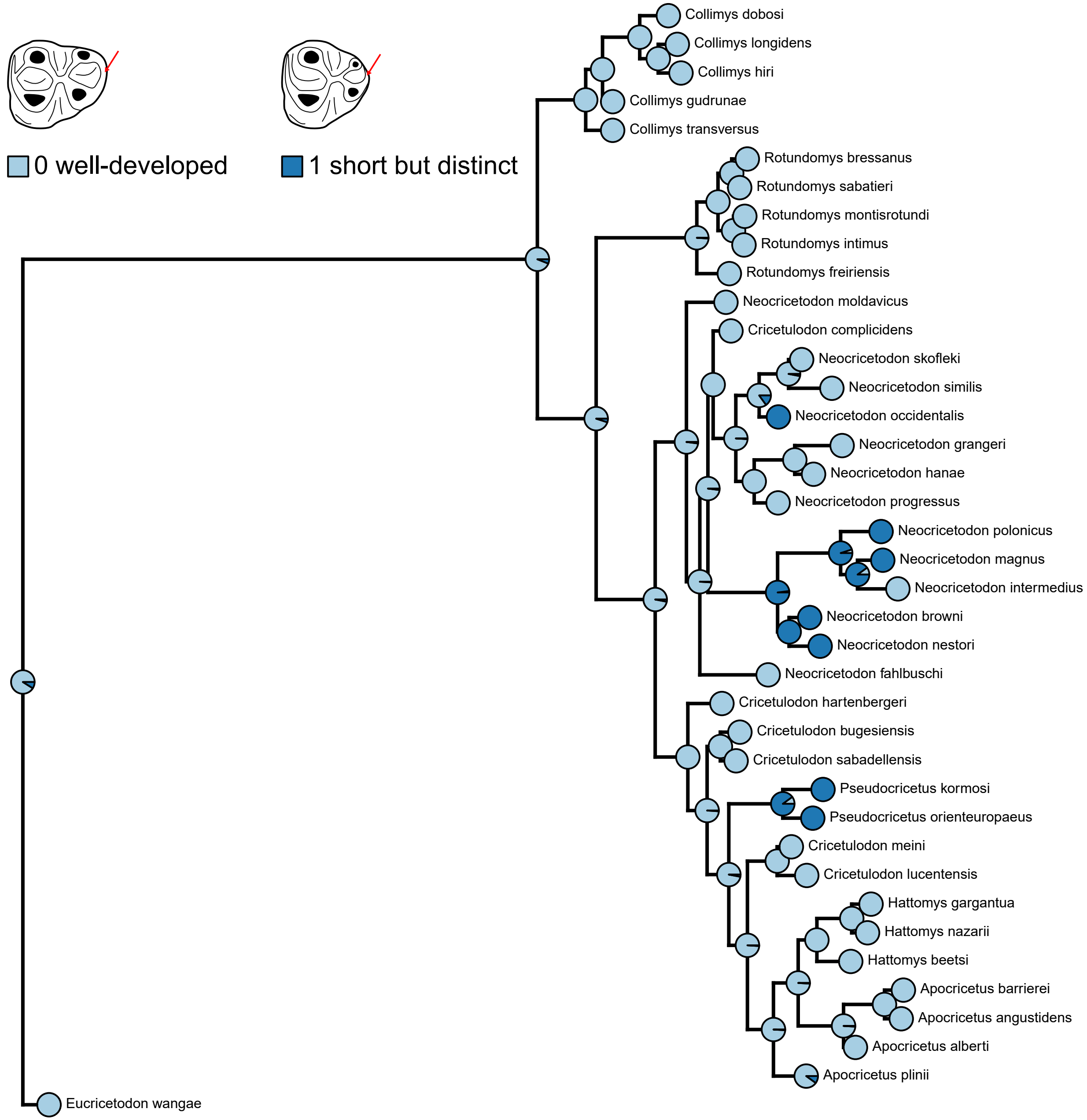

Ancestral state reconstruction of trait 57: m1 anteroconid

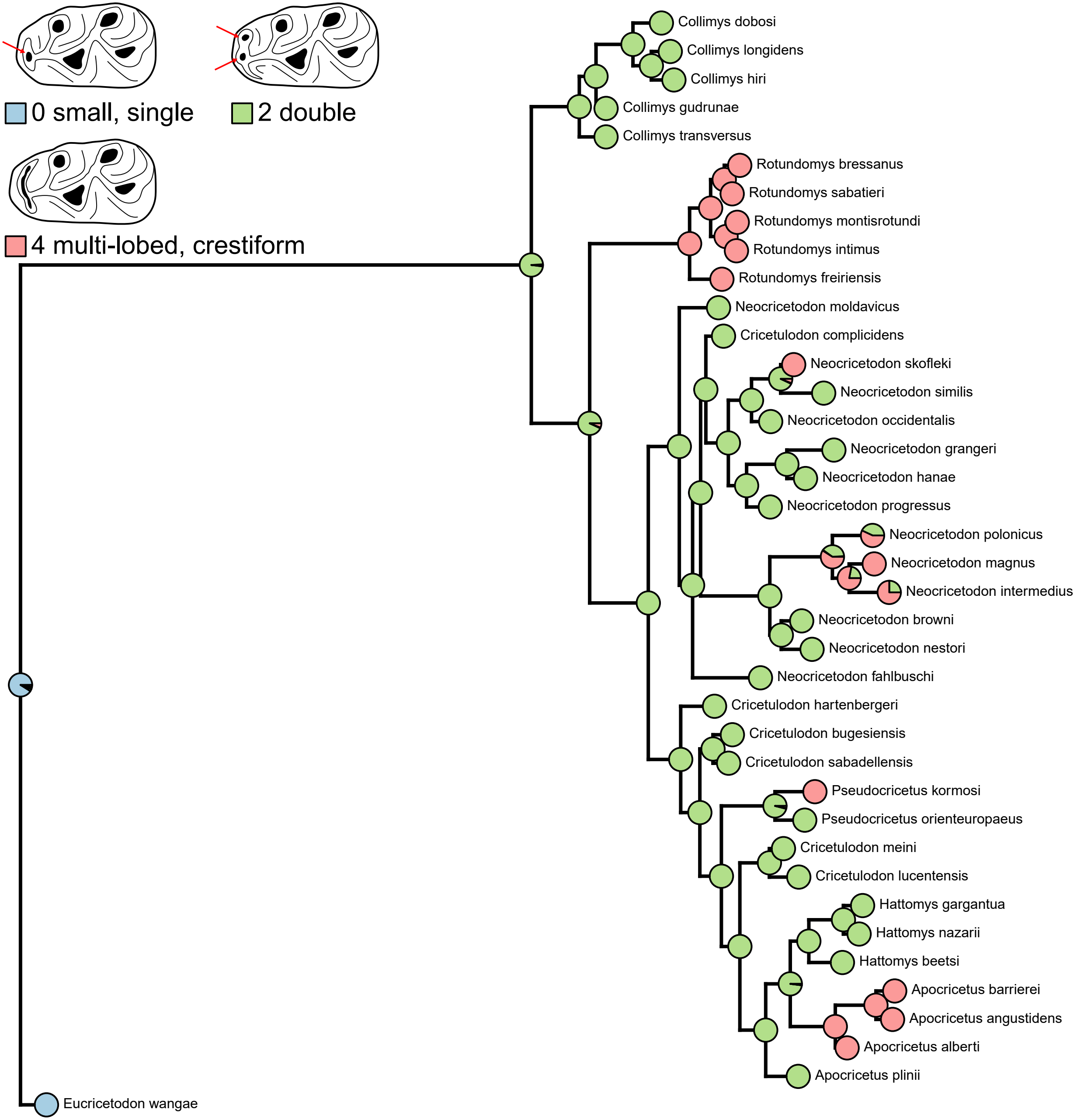

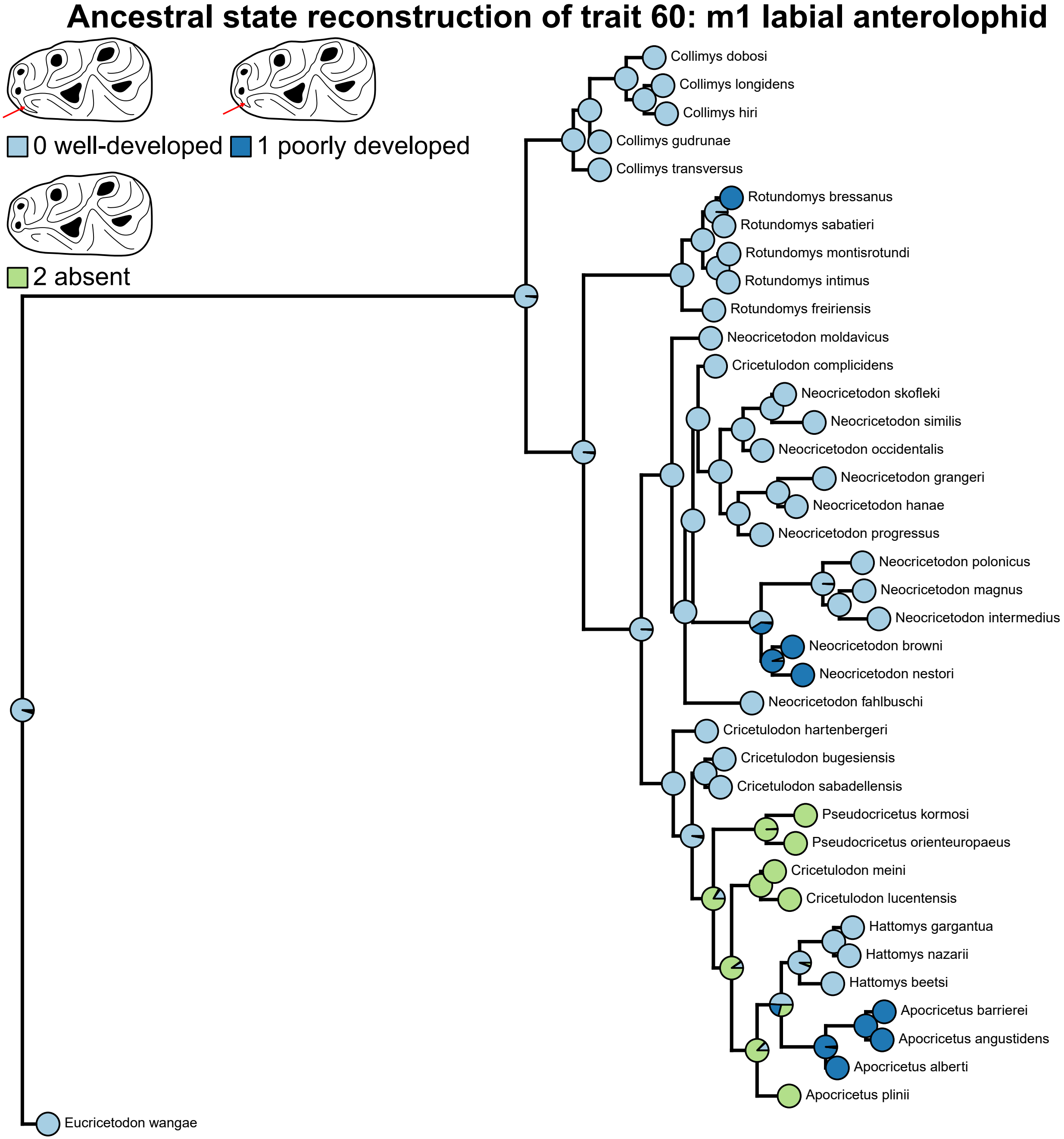
Ancestral state reconstruction of trait 60: m1 labial anterolophid
0 well-developed
1 poorly developed
2 absent
Collimys dobosi
Collimys longidens
Collimys hiri
Collimys gudrunae
Collimys transversus
Rotundomys bressanus
Rotundomys sabatieri
Rotundomys montisrotundi
Rotundomys intimus
Rotundomys freiriensis
Neocricetodon moldavicus
Cricetulodon complicidens
Neocricetodon skofleki
Neocricetodon similis
Neocricetodon occidentalis
Neocricetodon grangeri
Neocricetodon hanae
Neocricetodon progressus
Neocricetodon polonicus
Neocricetodon magnus
Neocricetodon intermedius
Neocricetodon browni
Neocricetodon nestori
Neocricetodon fahlbuschi
Cricetulodon hartenbergeri
Cricetulodon bugesiensis
Cricetulodon sabadellensis
Pseudocricetus kormosi
Pseudocricetus orienteuropaeus
Cricetulodon meini
Cricetulodon lucentensis
Hattomys gargantua
Hattomys nazarii
Hattomys beetsi
Apocricetus barrierei
Apocricetus angustidens
Apocricetus alberti
Apocricetus plinii
Eucricetodon wangae

Ancestral state reconstruction of trait 64: m1 labial spur of the anterolophulid

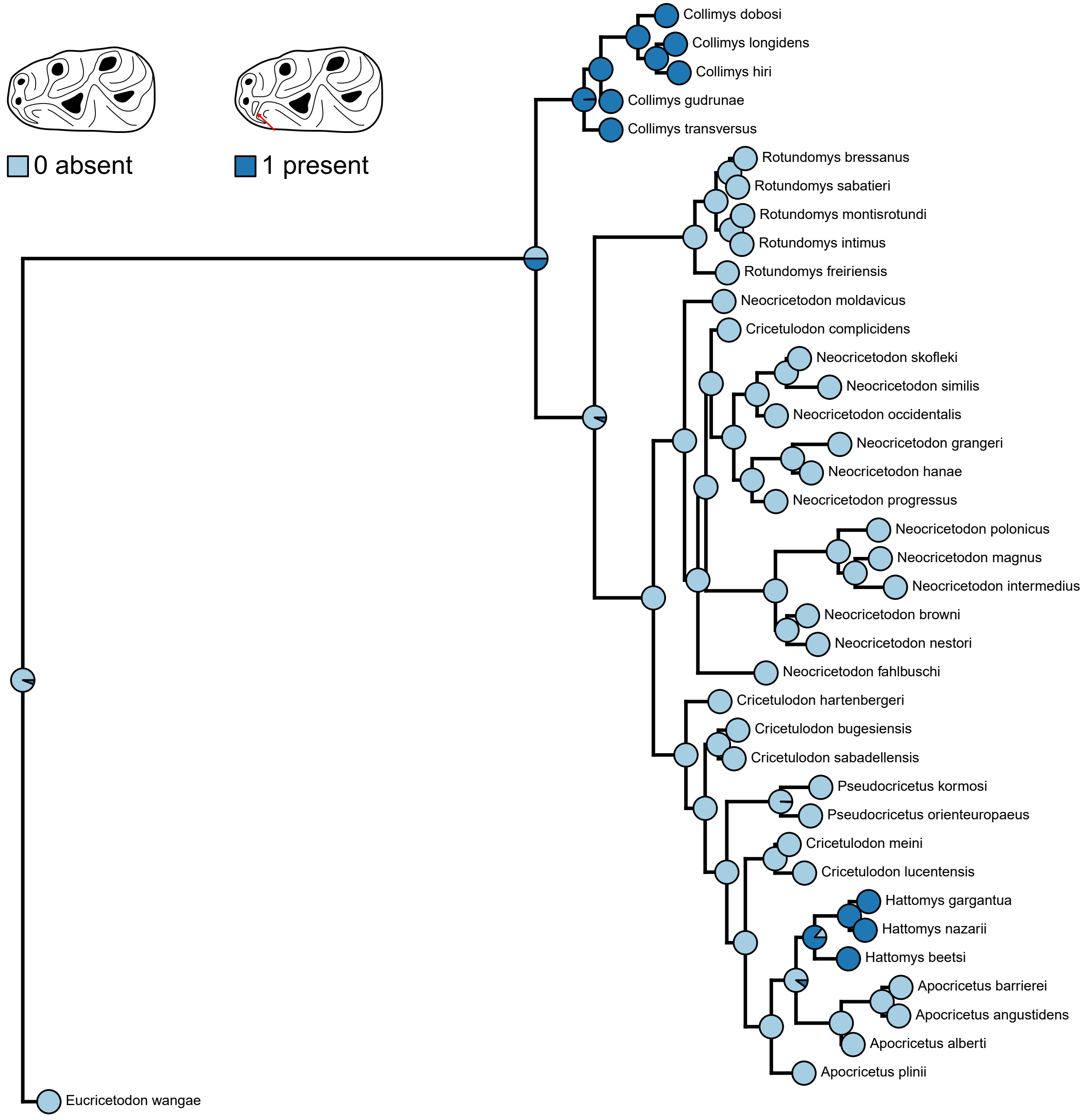

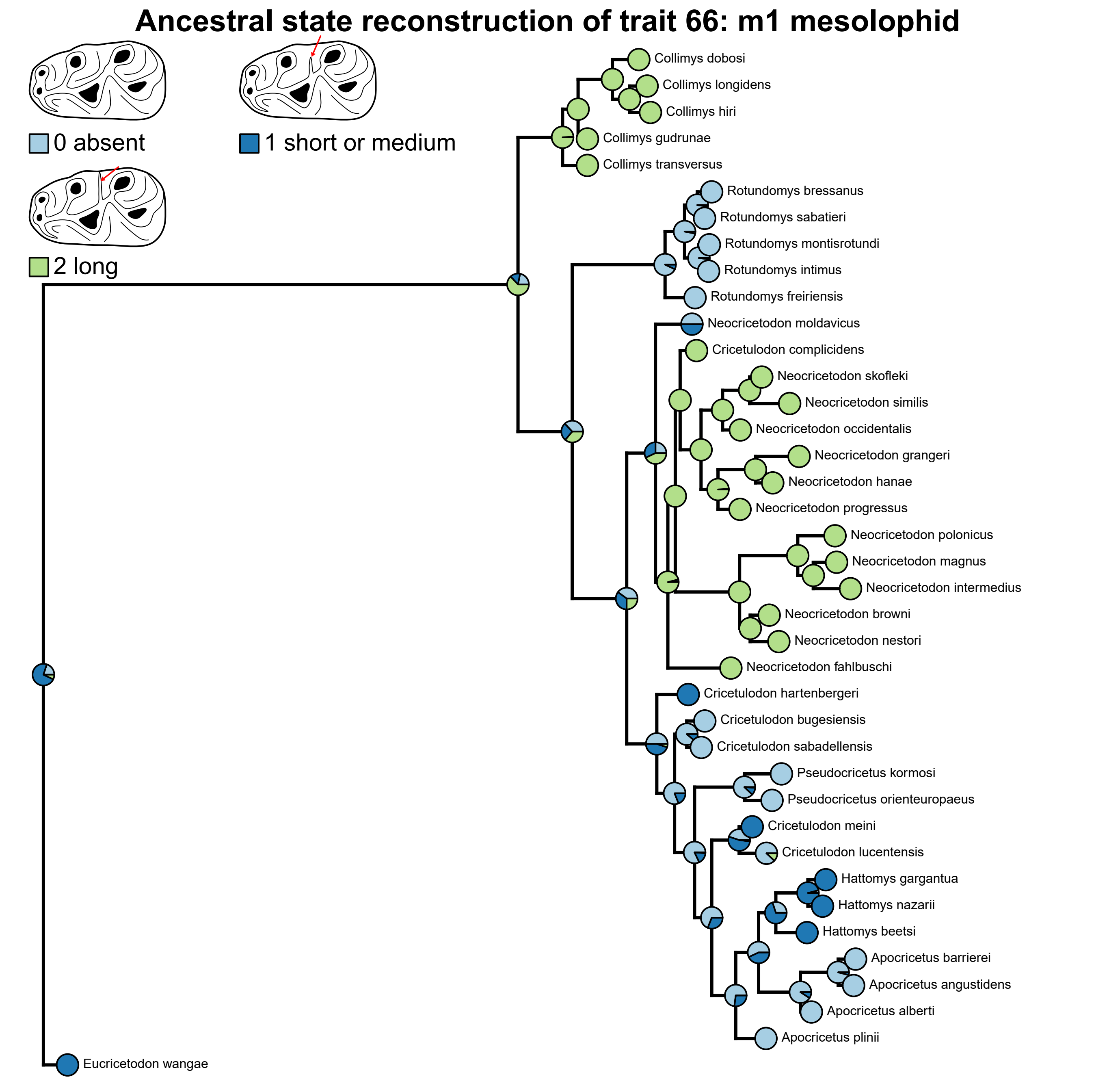
Ancestral state reconstruction of trait 66: m1 mesolophid
0 absent
1 short or medium
2 long
Collimys dobosi
Collimys longidens
Collimys hiri
Collimys gudrunae
Collimys transversus
Rotundomys bressanus
Rotundomys sabatieri
Rotundomys montisrotundi
Rotundomys intimus
Rotundomys freiriensis
Neocricetodon moldavicus
Cricetulodon complicidens
Neocricetodon skofleki
Neocricetodon similis
Neocricetodon occidentalis
Neocricetodon grangeri
Neocricetodon hanae
Neocricetodon progressus
Neocricetodon polonicus
Neocricetodon magnus
Neocricetodon intermedius
Neocricetodon browni
Neocricetodon nestori
Neocricetodon fahlbuschi
Cricetulodon hartenbergeri
Cricetulodon bugesiensis
Cricetulodon sabadellensis
Pseudocricetus kormosi
Pseudocricetus orienteuropaeus
Cricetulodon meini
Cricetulodon lucentensis
Hattomys gargantua
Hattomys nazarii
Hattomys beetsi
Apocricetus barrierei
Apocricetus angustidens
Apocricetus alberti
Apocricetus plinii
Eucricetodon wangae

Ancestral state reconstruction of trait 68: m1 ectomesolophid

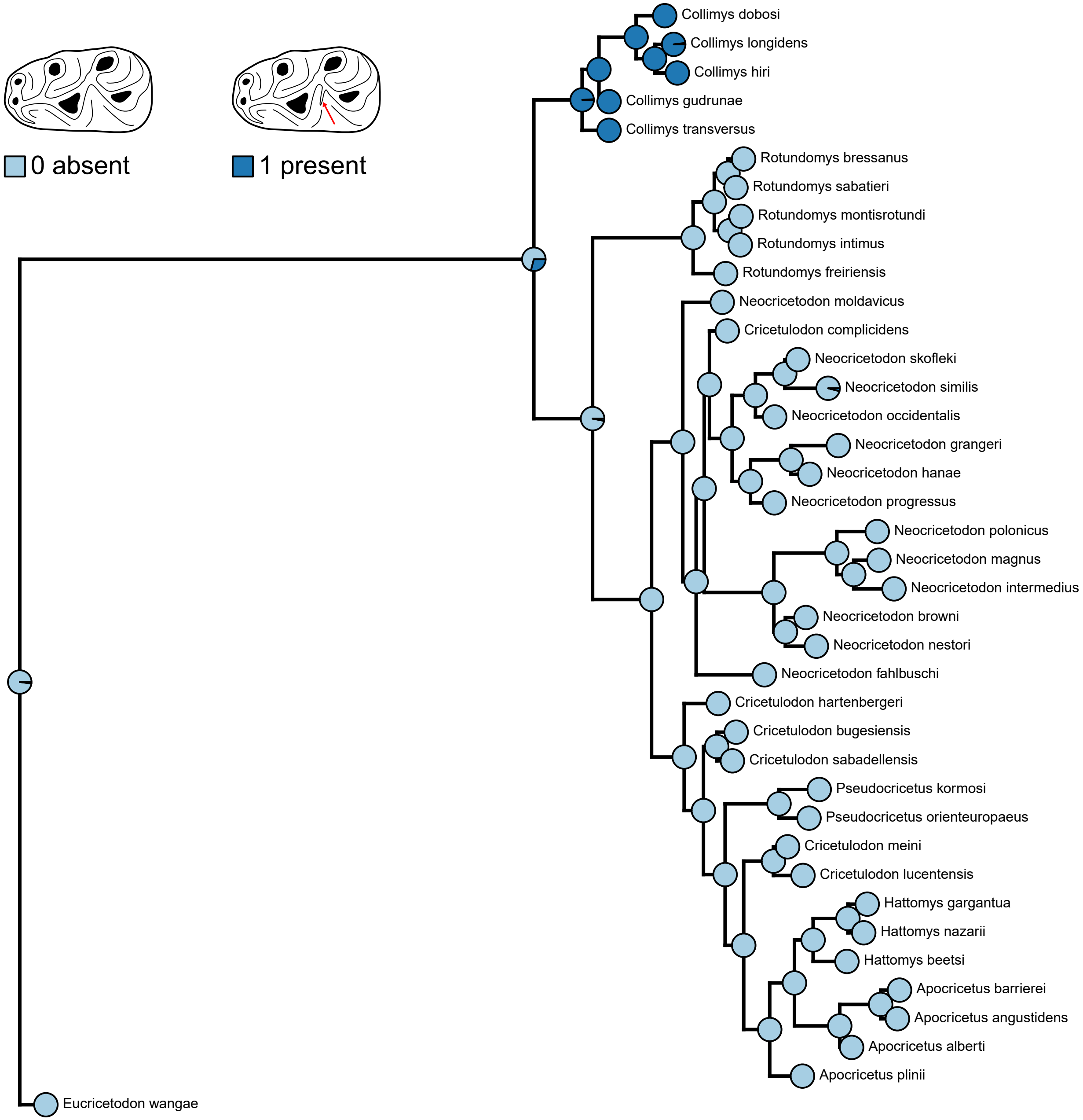

Ancestral state reconstruction of trait 73: m2 mesolophid

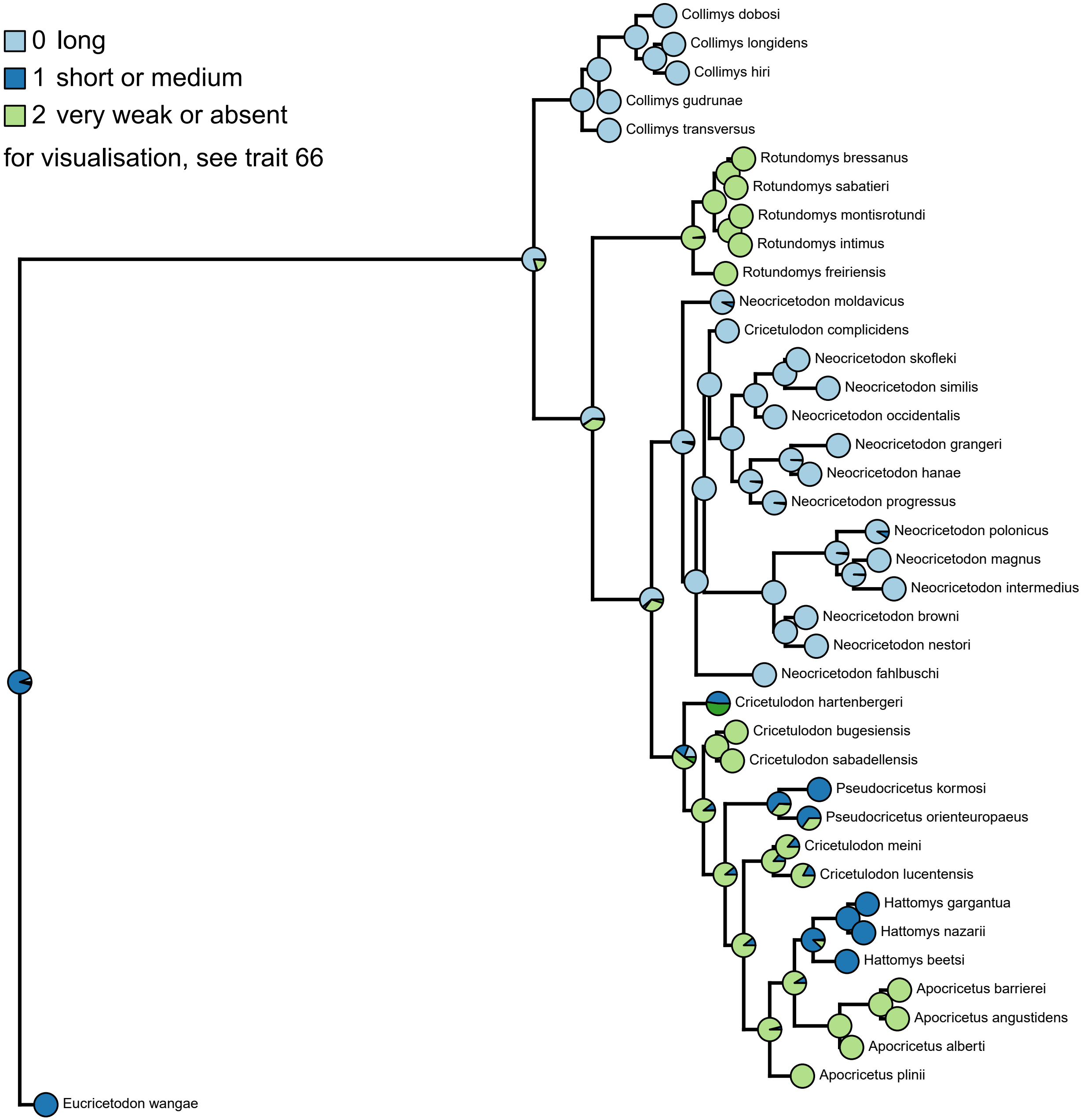

Ancestral state reconstruction of trait 73: m3 lingual anterolophid

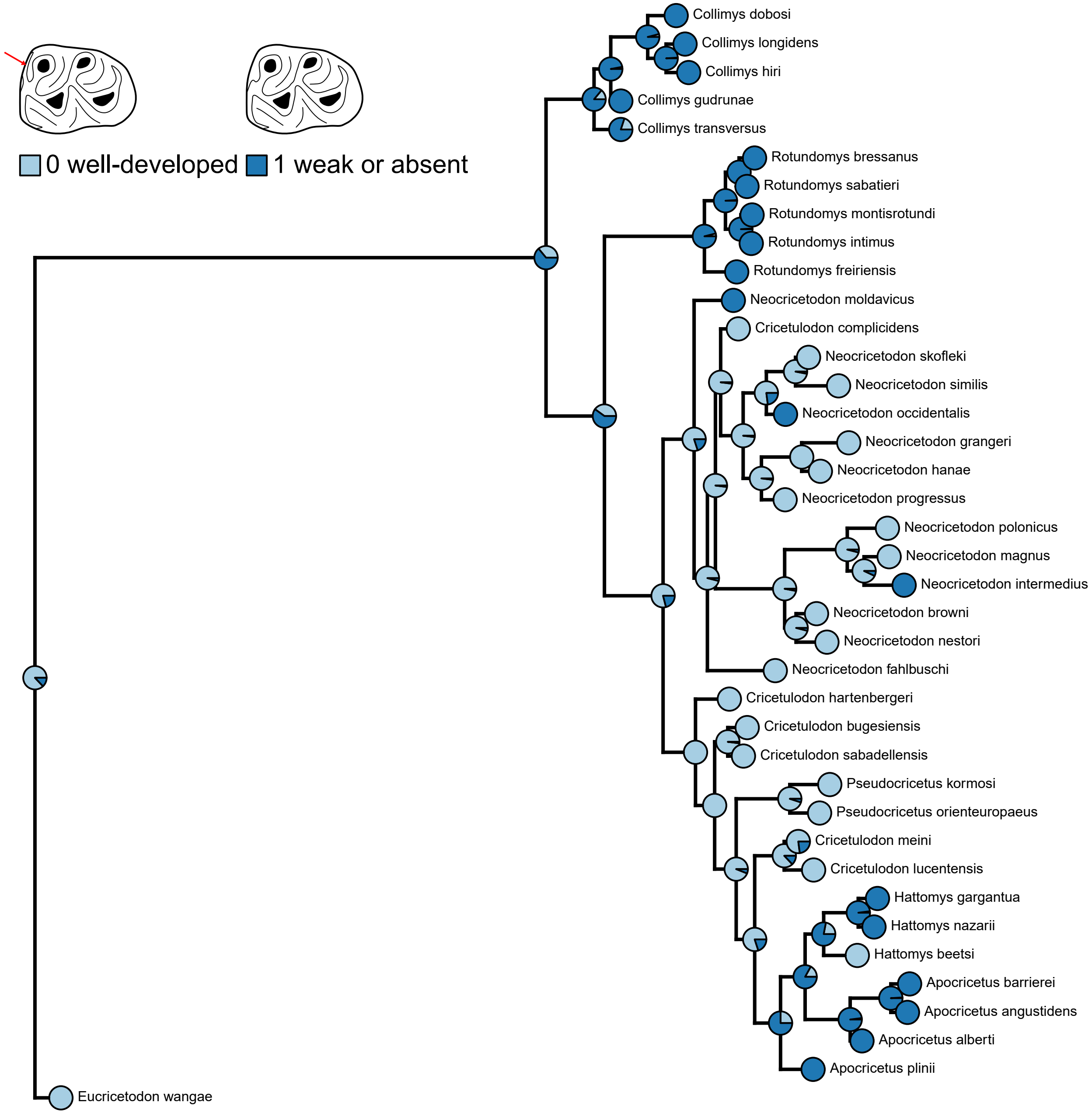

Ancestral state reconstruction of trait 88: m3 labial anterolophid

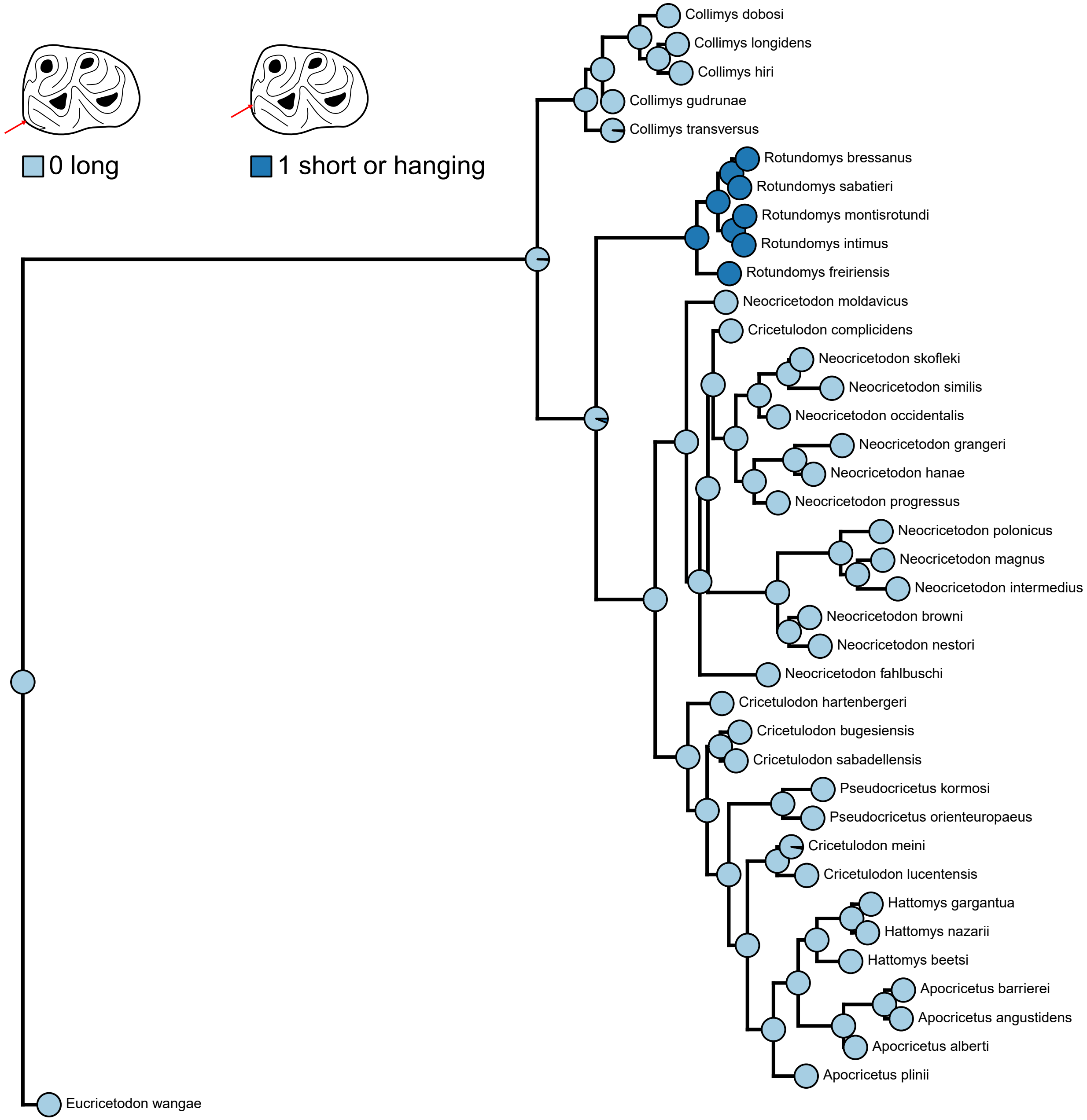

Ancestral state reconstruction of trait 103: M1 labial spur of the anterolophule

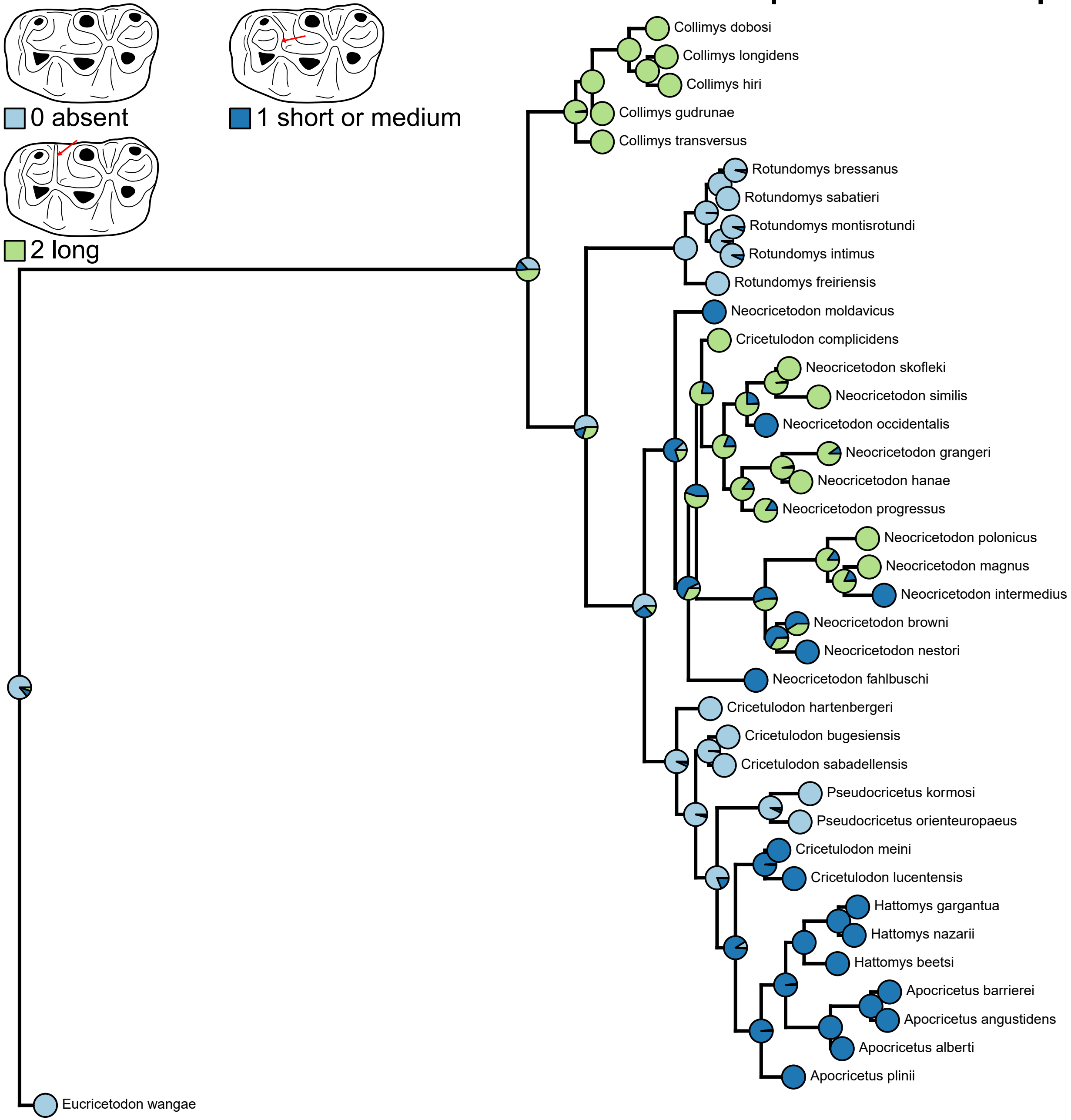

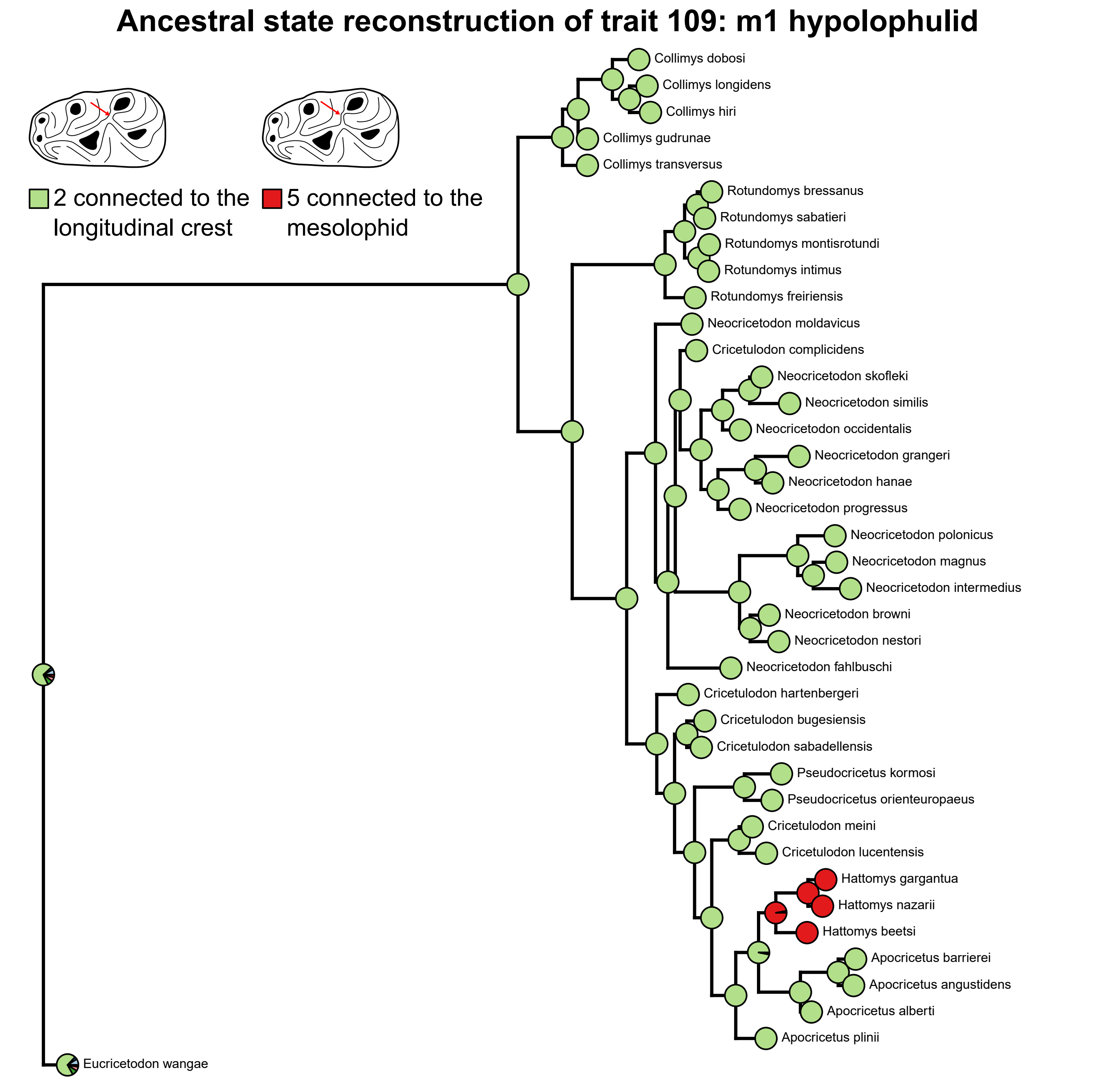
Ancestral state reconstruction of trait 109: m1 hypolophulid
2 connected to the longitudinal crest
5 connected to the mesolophid
Collimys dobosi
Collimys longidens
Collimys hiri
Collimys gudrunae
Collimys transversus
Rotundomys bressanus
Rotundomys sabatieri
Rotundomys montisrotundi
Rotundomys intimus
Rotundomys freiriensis
Neocricetodon moldavicus
Cricetulodon complicidens
Neocricetodon skofleki
Neocricetodon similis
Neocricetodon occidentalis
Neocricetodon grangeri
Neocricetodon hanae
Neocricetodon progressus
Neocricetodon polonicus
Neocricetodon magnus
Neocricetodon intermedius
Neocricetodon browni
Neocricetodon nestori
Neocricetodon fahlbuschi
Cricetulodon hartenbergeri
Cricetulodon bugesiensis
Cricetulodon sabadellensis
Pseudocricetus kormosi
Pseudocricetus orienteuropaeus
Cricetulodon meini
Cricetulodon lucentensis
Hattomys gargantua
Hattomys nazarii
Hattomys beetsi
Apocricetus barrierei
Apocricetus angustidens
Apocricetus alberti
Apocricetus plinii
Eucricetodon wangae
